# Exploring Synthetic Dihydrobenzofuran and Benzofuran Neolignans as Antiprotozoal Agents against *Trypanosoma cruzi*

**DOI:** 10.3390/pharmaceutics15030754

**Published:** 2023-02-24

**Authors:** Mariana C. Pagotti, Herbert J. Dias, Ana Carolina B. B. Candido, Thaís A. S. Oliveira, Alexandre Borges, Nicoli D. Oliveira, Carla D. Lopes, Renato P. Orenha, Renato L. T. Parreira, Antônio E. M. Crotti, Lizandra G. Magalhães

**Affiliations:** 1 Research Group on Natural Products, Center for Research in Sciences and Technology, University of Franca, Franca 14404-600, SP, Brazil; 2 Department of Chemistry, Faculty of Philosophy, Sciences and Letters, University of São Paulo, Ribeirão Preto 14040-901, SP, Brazil; 3 Goiano Federal Institute of Education, Science, and Technology, Campus Urutaí, Urutaí 75790-000, GO, Brazil; 4 Faculty of Medicine, University Center of Santa Fe do Sul, Santa Fé do Sul 15775-000, SP, Brazil; 5 Animal Science Post Graduation, University of Franca, Franca 14404-600, SP, Brazil; 6 Department of Biochemistry and Immunology, Medical School of Ribeirão Preto, University of São Paulo, Ribeirão Preto 14049-900, SP, Brazil

**Keywords:** antiparasitic, Chagas disease, dihydrobenzofuran-type neolignans, *Trypanosoma cruzi*

## Abstract

Chagas disease is a neglected tropical disease that affects more than 8 million people. Although there are therapies against this disease, the search for new drugs is important because the current treatments show limited effectiveness and high toxicity. In this work, eighteen dihydrobenzofuran-type neolignans (DBNs) and two benzofuran-type neolignans (BNs) were synthesized and evaluated against amastigote forms of two *Trypanosoma cruzi* strains. The in vitro cytotoxicity and hemolytic activity of the most active compounds were also evaluated and their relationships with *T. cruzi* tubulin DBNs were investigated by an in silico approach. Four DBNs demonstrated activity against the *T. cruzi* Tulahuen lac-Z strain (IC_50_ from 7.96 to 21.12 µM), and DBN **1** exhibited the highest activity against the amastigote forms of the *T. cruzi* Y strain (IC_50_ 3.26 μM). Compounds **1**–**4** showed CC_50_ values higher than antitrypanosomal activities, except for DBN **3**. All DBNs with antitrypanosomal activity demonstrated CH_50_ higher than 100 µM. The in silico results indicated that DBNs **1**, **2**, and **4** are capable of destabilizing the dynamics of the tubulin-microtubule from the vinca site. These compounds displayed promising in vitro activity against *T. cruzi*, especially compound **1**, and can be considered molecular prototypes for the development of new antiparasitic drugs.

## 1. Introduction

Chagas disease is a neglected tropical disease (NTD) caused by the parasite *Trypanosoma cruzi*, and its transmission can occur by contact with contaminated blood, congenital and oral routes, and by contamination with triatomine vector feces [1]. This disease affects about 6–7 million people in more than 20 countries in Latin America and is responsible for 1–2 million deaths every year [2].

The treatment is restricted to two nitroheterocyclic drugs, benznidazole (BZN) and nifurtimox. Although they are very efficient in treating acute infections, these drugs have frequent undesirable side effects and have limited efficacy in the chronic phase of the disease [3]. Furthermore, BZN and nifurtimox show high toxicity and require a prolonged treatment period [4], so they are not considered ideal [5,6].

According to molecular genetics, eco-epidemiological and pathogenicity features, *T. cruzi* populations have been classified into at least seven discrete typing units (DTUs) [7,8,9,10] and susceptibility to reference drugs can vary among *T. cruzi* strains [11,12]. In this worrisome scenario, the search for new drugs against Chagas disease is important, especially in developing countries.

In the research of new hit/lead compounds against trypanosomatids, many research groups have previously evaluated the activity of neolignans, which comprise an important class of phenolic structures derived from the oxidative coupling of allyl phenols and propylphenols [13]. Among neolignans, dihydrobenzofuran-type neolignans (DB**Ns**) and benzofuran-type neolignans (BNs) have attracted attention due to their structural diversity and their broad range of biological activities, including leishmanicidal and antitrypanosomal activities [14,15,16,17]. For instance, 2,3-dihydrobenzofuran neolignan (2,3-DBN), which is commonly found in propolis and other plants, inhibits the growth of promastigote and amastigote forms of *L*. (L.) *amazonensis* [17]. Besides, DBNs have also demonstrated activity against axenic amastigotes of *L. donovani* [14] and against trypomastigote forms of *T. cruzi* [15]. The biological activities of DBNs against trypanosomatids also can be attributed to the inhibition of tubulin polymerization, a heterodimeric protein involved in various cellular processes that is considered a target for antiprotozoal chemotherapy [14].

**Scheme 1:**
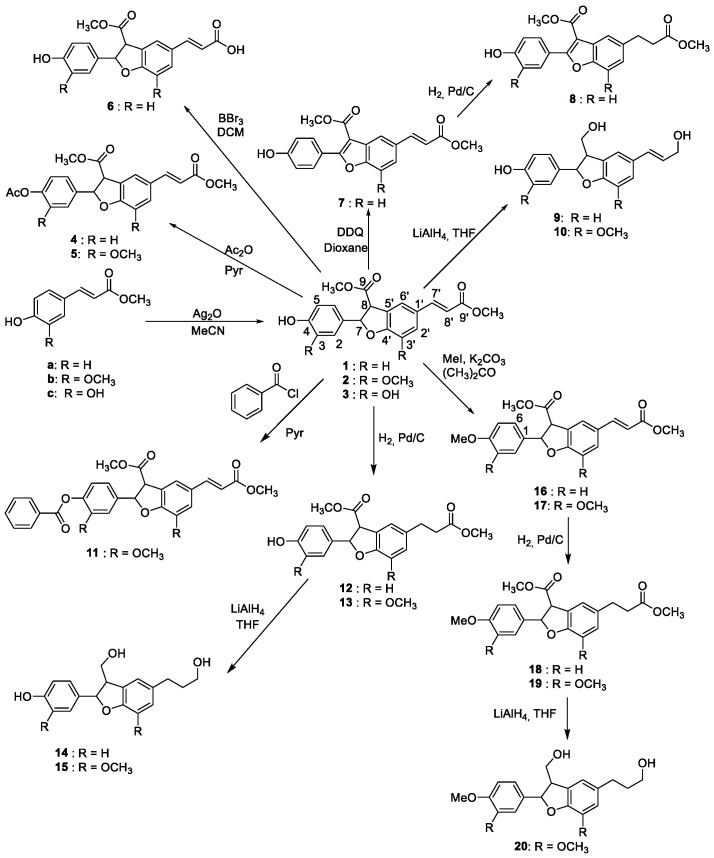
Synthesis of the DBNs **1**–**6**, **9**–**20,** and BNs **7**–**8**. MeCN: acetonitrile; Ac_2_O: acetic anhydride; Pyr: pyridine; DCM: dichloromethane; THF: tetrahydrofuran. Compounds **1**–**6** and **9**–**20** were obtained as a racemic mixture of *trans*-enantiomers (stereochemistry not shown).

Based on these previous reports in the literature, and as part of our ongoing research on the antiparasitic activity of synthetic compounds analogs to natural products [12,18], this work aimed to exploit the antitrypanosomal effects of eighteen synthetic DBNs and two synthetic BNs (Scheme 1) on the two *T. cruzi* strains and their cytotoxicity on mammalian cells under in vitro experimental conditions. The relationship between the most active compounds and their *T. cruzi* tubulin was also investigated by an in silico approach.

## 2. Materials and Methods

### 2.1. Synthesis of Compounds ***1**–**20***

DBNs **1**, **2**, and **3** were synthesized by oxidative coupling of methyl *p*-coumarate (**a**), methyl ferulate (**b**), and methyl caffeate (**c**), respectively (Scheme 1), as previously reported [19,20]. Compounds **4**–**20** were synthesized from **1** or **2** by the previously reported methodologies [21,22,23]. Briefly, the methyl esters **a**, **b**, and **c** (16.0 mmol) were dissolved in acetonitrile (40 mL) in a round-bottom flask covered by aluminum foil, then Ag_2_O (8.0 mmol) was added as the oxidizer. The reactions were stirred under an N_2_ atmosphere at room temperature for 4 h. Next, the oxidizer was filtered off and the resulting crude reaction mixtures were purified by column chromatography (2.2 × 100 cm, silica gel 60, 0.040–0.063 mm) using hexane and ethyl acetate (2:1 *v*/*v*) as eluent to give **1** (36% yield), **2** (43% yield), and **3** (12% yield) as racemic mixtures of *trans*-enantiomers. Neolignans **1** and **2** were converted into **4** and **5** by acetylation with acetic anhydride as previously reported by Kuo and co-workers with modifications [21]. To this end, compounds **1** (1.15 mmol) and **2** (1.30 mmol) were solubilized in pyridine (7 mL) in a three-necked flask equipped with a magnetic stirrer, then acetic anhydride (7 mL) was added. The reaction was kept under stirring and N*_2_* atmosphere at room temperature for 48 h. The solvent was evaporated under reduced pressure to afford products **4** (82% yield) and **5** (96% yield).

The synthesis of compound **6** was based on the work reported by Felix [24]. To this end, compound **1** (0.85 mmol) was dissolved in 20 mL dichloromethane (DCM) in a three-necked flask equipped with a magnetic stirrer at room temperature under an N_2_ atmosphere. Boron tribromide (BBr_3_, 8.5 mmol) was added, and the mixture was maintained for 7 h. The crude reaction mixture was washed with water (3 × 10 mL) and the organic phase was chromatographed over silica gel (0.8 × 40 cm, silica gel 60, 0.040–0.063 mm), using as eluent a mixture of hexane and ethyl acetate (5:1 *v*/*v*) to afford **6** (38% yield).

Compound **7** was obtained from **1** as previously reported [21]. To this end, in a round-bottomed flask equipped with a magnetic stirrer under an inert atmosphere (N_2_), DBN **1** (1.4 mmol) was dissolved in dioxane (10 mL) and added to 2,3-dichloro-5,6-dicyano-1,4-benzoquinone (DDQ, 1.8 mmol) as the oxidizer. The reaction mixture was maintained under reflux for 22 h, filtered, and evaporated under reduced pressure to give the crude reaction mixture. Purification of the crude reaction mixture by column chromatography (1.2 × 60 cm, silica gel 60, 0.040–0.063 mm) using hexane and ethyl acetate (1:2 *v*/*v*) as eluent gave compound **7** (74% yield).

Compounds **8**, **12**, and **13** were obtained following the methodology previously reported by Pieters and co-workers [23]. To this end, compounds **1**, **2**, and **7** (0.6 mmol) were dissolved in 40 mL dry acetone, and 0.3 g of Pd/C (5%) was added to the mixtures, which were put up in a high-pressure reactor equipped with a magnetic stirrer and H_2_ (60 psi) at room temperature for 2 h. The mixtures were filtered off to remove the catalyst, and the solvent was removed under reduced pressure to give **8** (94% yield), **12** (95% yield), and **13** (88% yield), respectively.

Compound **11** was obtained from **2** as described by Kuo and co-workers with modifications [21]. To this end, compound **2** (1.1 mmol) was dissolved in pyridine (10 mL) in a three-necked flask equipped with a magnetic stirrer. Next, benzoyl chloride (0.2 mL) was added. The mixture was kept under stirring at room temperature for 20 h. The crude mixture was evaporated under reduced pressure, washed with toluene and dichloromethane, and chromatographed over silica (silica gel 60, 0.040–0.063 mm, tube dimensions 1.2 × 60 cm) with hexane and ethyl acetate (1:1 *v*/*v*) to obtain **11** (95% yield).

DBNs **1** and **2** were methylated as described in previous reports [23]. These starting materials (1.3 mmol) were dissolved in dry acetone (20 mL) in a three-necked flask equipped with a magnetic stirrer. Next, K_2_CO_3_ (2.6 mmol) and CH_3_I (2.6 mmol) were added and kept under stirring and an N_2_ atmosphere for 24 h. The mixtures were filtered and purified by column chromatography (1.2 × 60 cm, silica gel 60, 0.040–0.063 mm) with hexane and ethyl acetate (3:2 *v*/*v*), followed by recrystallization with ether to give **16** (22% yield) and **17** (18% yield).

Compounds **16** and **17** were further hydrogenated by the same procedure described above. Briefly, compounds **16** and **17** (0.2 mmol) were dissolved in dry acetone (20 mL) and added to a high-pressure reactor containing Pd/C (5%, 0.15g) and kept for 2 h under magnetic stirring and H_2_ pressure (60 psi). The crude reaction mixtures were filtered and dried under reduced pressure to give **18** (90% yield) and **19** (91% yield), respectively.

Compound **19** was used to obtain 4-O-methylcedrusin (**20**) through reduction with LiAlH_4_, as described by Pieters and co-workers [23]. To this end, compound **19** (0.15 mmol) was dissolved in dry THF (10 mL) in a three-necked flask, then LiAlH_4_ (0.15 mmol) was dissolved in dry THF (5 mL) and slowly added using an addition funnel. The mixture was stirred under an N_2_ atmosphere for 2 h at 0 °C. The reaction mixture was worked up by the addition of H_2_O and then concentrated HCl. The mixture was transferred to a separation funnel, and the organic layer was collected, concentrated under reduced pressure, and further purified by column chromatography (0.8 × 40 cm, silica gel 60, 0.040–0.063 mm) using a mixture of methanol and dichloromethane (2:8 *v*/*v*) as eluent to give 4-O-methylcedrusin (**20**, 49% yield). A similar procedure was applied to reduce **1** (0.75 mmol), **2** (0.75 mmol), **12** (0.4 mmol), and **13** (0.4 mmol) into **9** (62% yield), **10** (60% yield), **14** (59% yield), and **15** (52% yield), respectively.

All the structures were confirmed by NMR analyses (Table S1, Figures S1–S40, Supplementary Material). Purity of compounds **1**–**20** was assessed by gas chromatography with flame ionization detection (GC-FID) (Figures S41–S60, Supplementary Material). A minimum purity of 90% was required for the biological assays.

### 2.2. Animals

Male Swiss mice (*Mus musculus*, 6 weeks old) were supplied by the animal house of the University of São Paulo, Ribeirão Preto, BR. The animals were housed in ventilated cage racks (2 or 5 animals per cage) at the animal house facility of the University of Franca (UNIFRAN) under controlled conditions and received water and food ad libitum (Labina, São Paulo, BR).

### 2.3. Parasites and Mammalian Cell Maintenance

Monkey (*Macaca mulata*) epithelial kidney cell line LLC-MK2 (ATCC^®^ CCL-7™) was cultivated in RPMI 1640 medium (Roswell Park Memorial Institute; Gibco-Life Technology, Grand Island, NY, USA) supplemented with 10% fetal bovine serum (SFB) and antibiotics (penicillin: 10,000 UI/mL and streptomycin: 10,000 mg/mL) and incubated at 37 °C in a 5% CO_2_ humidified incubator (Sanyo, Osaka, Japan) [25]. The total spleen cell suspension was prepared using organs obtained from Swiss mice and macerated with RPMI 1640 medium (Gibco-Life Technology, Grand Island, NY, USA) in a Petri dish using a nylon sieve and a rubber stopper. The cell suspension was centrifuged at 250× *g* for 10 min, and then the pellet was resuspended in RPMI 1640 medium (Gibco-Life Technology, Grand Island, NY, USA) and cultivated and maintained as described for LLC-MK2.

Two strains of *T. cruzi* were used in this study: a *T. cruzi* Tulahuen strain stably expressing the β-galactosidase gene from *Escherichia coli* (Tulahuen lac-Z) [26], which is classified as drug-sensitive [11,27]; and a *T. cruzi* Y strain isolated from an acute human case [11,27,28] and classified as drug-moderately resistant [27].

Trypomastigotes of *T. cruzi* (Tulahuen lac-Z) were maintained weekly in LLC-MK2 cells, cultivated in RPMI 1640 medium (Gibco-Life Technology, Grand Island, NY, USA) without phenol red supplemented with antibiotics (penicillin—10,000 UI/mL and streptomycin—10,000 mg/mL) and 10% fetal bovine serum (FBS; Cultilab, Campinas, Brazil), and incubated at 37 °C in 5% CO_2_ humidified incubator (Sanyo, Osaka, Japan). Bloodstream trypomastigotes of the Y strain were obtained from infected mice at the parasitemia peak and maintained in cultivating as described above for LLC-MK2 cells.

Murine spleen cells were obtained by maceration of Swiss mice spleens in RPMI 1640 medium (Gibco-Life Technology, Grand Island, NY, USA), and then cultivated in RPMI 1640 medium (Gibco-Life Technology, Grand Island, NY, USA), supplemented with antibiotics (penicillin—10,000 UI/mL and streptomycin—10,000 mg/mL) and 10% FBS (Cultilab, Campinas, SP, Brazil), and incubated at 37 °C in a 5% CO_2_ humidified incubator (Sanyo) [25]. LLC-MK2 cells also were cultivated and maintained as described for murine spleen cells.

### 2.4. Evaluation In Vitro of Antitrypanosomal Activity

#### 2.4.1. Antitrypanosomal Activity against Drug-Sensitive *T. cruzi* Tulahuen Lac-Z Strain

An initial screening against amastigote forms of the *T. cruzi* Tulahuen lac-Z strain was performed as described by Maia and co-workers [25]. Briefly, LLC-MK2 cells were sown at 2 × 10^4^ cells per mL in 96-well plates (TPP, Trasadingen, Switzerland) for 24 h and then infected with the trypomastigote forms of the *T. cruzi* Tulahuen strain, which were obtained from the supernatants of LLC-MK2 infected cultures harvested between days 5 and 8 of infection [29], for 48 h. After the infection period, the plates were washed to remove the trypomastigote forms not internalized, and the cells infected with the intracellular form of *T. cruzi* were incubated with compounds **1**–**20** dissolved in dimethylsulphoxide (DMSO; Sigma-Aldrich, St. Louis, MO, USA) added at concentrations from 0.24 to 500 µM. After 72 h of culture, a 50 µL phosphate-saline buffer (PBS) containing 0.5% of Triton X-100 (Sigma-Aldrich, St. Louis, MO, USA) and 100 µM chlorophenol red-β-d-galactosidase (CPRG, Sigma-Aldrich, St. Louis, MO, USA) was added. After 4 h of incubation at 37 °C, the absorbance was measured at 570 nm with a spectrophotometer (Biochrom Corp, Miami, FL, USA), and the absorbance values were used to obtain the 50% inhibitory concentration (IC_50_) values, which corresponds to the concentration that leads to a 50% reduction in the parasitemia inside the host cell. BZN (0.24-500 µM; Sigma-Aldrich, St. Louis, MO, USA) was used as the positive control, and RPMI 1640 medium (Gibco, Grand Island, NY, USA) with 0.1% DMSO was employed as the negative control. The results of parasite viability were determined at the base of the catalysis of CPRG by β-galactosidase.

#### 2.4.2. Antitrypanosomal Activity against Moderately Drug-Resistant *T. cruzi* Y Strain

The activity against amastigotes forms of the *T. cruzi* Y strain was determined as described by Maia and co-workers [25]. LLC-MK2 cells were sown at 2 × 10^5^ cells per well in 24-well plates on glass coverslips (13 mm; Global Glass, São Paulo, BR) and incubated at 37 °C in a 5% CO_2_ humidified incubator (Sanyo) for 24 h. These cells were then infected with 1 × 10^6^ trypomastigote forms of the *T. cruzi* Y strain, and after 48 h of infection, the plates were washed to remove the trypomastigotes not internalized. The cells infected with the intracellular form of *T. cruzi* were incubated with DBNs **1**, **2**, **3**, and **4** (which were the most active in the initial screening) previously dissolved in DMSO (Sigma-Aldrich, St. Louis, MO, USA) at concentrations ranging from 1.56 to 25 µM for 72 h (noncytotoxic concentrations). The coverslip slides were stained with Giemsa (Synth, São Paulo, Brazil) and analyzed using an optical microscope (Nikon, New York, NY, USA). The parasite load was defined by the number of infected macrophages X the number of intracellular amastigotes/the number of total macrophages, and the IC_50_ values were calculated. BZN (1.56-25 µM; Sigma-Aldrich, St. Louis, MO, USA) was used as the positive control, and RPMI 1640 medium (Gibco, Grand Island, NY, USA) with 0.1% DMSO was employed as the negative control. The results were expressed as mean infected cell percentage and amastigote reduction percentage relative to the negative control (0.1% DMSO).

### 2.5. Determination of In Vitro Cytotoxicity and Hemolytic Activities

The cytotoxicity of DBNs **1**–**4** was determined using the propidium iodide [25] and trypan blue methodologies [30]. To assess the cytotoxic activity using propidium iodide, mice spleen cells were sown at a concentration of 6.5 × 10^6^ cells/mL in a 24-well plate. DBNs **1**, **2**, **3**, **4**, and BZN were added at concentrations ranging from 1.95 to 250 µM and incubated for 24 h at 37 °C in a 5% CO_2_ humidified incubator (Sanyo). After the incubation period, the cells were harvested and incubated with a 10 µg/mL propidium iodide solution (Sigma-Aldrich, St. Louis, MO, USA). The cell acquisition was performed within 15 min using a flow cytometer (BD-FACSCanto™II; BD Biosciences, BR), and cell counting was used to obtain the CC_50_ (50% cytotoxic concentration). Tween 20 (Sigma-Aldrich, St. Louis, MO, USA) at 0.5% was used as a cell death positive control, and RPMI 1640 medium (Gibco, Grand Island, NY, USA) with 0.1% DMSO was used as the negative control.

To assess the cytotoxic activity of DBNs **1**–**4** using trypan blue, LLC-MK2 cells were sown at a concentration of 2 × 10^5^ cells per well in 96-well plates. DBNs **1**, **2**, **3**, **4**, and BZN previously dissolved with DMSO were added to achieve the concentration range of 6.25-100 µM and incubated at 37 °C in a 5% CO_2_ humidified incubator (Sanyo) for 72 h. Next, the cells were trypsinized for 30 s, and then 10 μL of the supernatant was stained with 10 μL of a 0.4% trypan blue solution (Synth, São Paulo, Brazil). Cell counting was performed using transmitted light microscopy (Nikon) with 40X magnification using a Neubauer’s chamber (Global Glass), with a total of 100 cells being counted, where cellsstained blue were considered non-viable [31,32]. Cell counting was used to obtain the CC_50_. DMSO (Sigma-Aldrich, St. Louis, MO, USA) at 25% was used as a cell death positive control, and RPMI 1640 medium (Gibco, Grand Island, NY, USA) with 0.1% DMSO was used as the negative control.

The hemolytic activity was determined as described by Kaplum and co-workers [33] with adaptations. Defibrinated sheep blood was diluted in 0.9% saline solution. Neolignans **1**, **2**, **3**, **4**, and BZN were solubilized in DMSO (Sigma-Aldrich, St. Louis, MO, USA) and added to microtubes containing red blood cells in concentrations from 6.25 to 100 µM. The plates were incubated for 30 min at 37 °C, the absorbance of the supernatant was read at 415 nm using a spectrophotometer (Biochrom Corp), and the absorbance values were used to obtain the CH_50_ (hemolytic concentration necessary to cause lysis of 50% of erythrocytes). Distilled water was used as a positive control, and a 0.9% saline solution with 0.1% DMSO was used as the negative control.

### 2.6. Computational Analysis

Tubulin protein belonging to *T. cruzi* was used as the target in the molecular docking performed in this study. However, because there are no crystallographic structures, the 3D structure of this protein was built through homology modeling [34]. For the α and β structures of *T. cruzi* tubulin, their respective amino acid sequences were used (UniprotKB’s access codes Q27352 for α-tubulin and P08562 for β-tubulin), and the 3D crystallographic model used was from *Rattus norvegicus* (RCSB PDB ID 402B). To correct the alignment of the *T. cruzi* α- and β-tubulin sequences, they were performed with the aid of the *T. brucei brucei*, *T. brucei rhodeniense*, and *L*. (V.) *braziliensis* sequences (with the respective UniprotKB access codes Q57XX3, P04106, and A4HE59). In addition, to obtain complete binding sites located at the interfaces between the α- and β-tubulin subunits and also between the β- and α-tubulin, an α1-β-α2-heterodimer was constructed, with the subunits modeled from the 3D crystallographic model (RCSB PDB ID 402B, *Rattus norvegicus*). The results of the homology modeling were based on the values of the Q-Mean scoring functions (Qualitative Model Energy Analysis) and the Ramachandran graph [35,36]. The simulation was performed in the GOLD 5.3 program [37], with the centroid defined from the 3D structures PDB ID 4O2B (colchicine) and PDB ID 4EB6 (vinblastine). As for tubulin, studies were carried out in four 3D structures: PDB ID 402B (for colchicine), PDB ID 4EB6 (for vinblastine), and two other sites from the amino acid residues of colchicine and vinblastine. For tubulin, a radius of 10Å was established from their respective centroids, and the scoring function chosen for both was GoldScore. The re-docking process required to validate the simulation used colchicine and vinblastine for tubulin, which were obtained from the crystallographic structures PDB ID 4O2B and 4EB6, respectively. The in silico study was carried out using the *trans*-enantiomers 7*R*,8*R* of DBNs **1**, **2**, and **4**, which were randomly chosen. Molecular docking calculations were performed using the GoldScore function, and analyses of the best poses and their corresponding intermolecular interactions for the two targets were performed at Discovery Studio 2016.

### 2.7. Statistical Analysis

In vitro experiments were performed in triplicate and repeated three times. The IC_50_ (inhibitory concentration necessary to cause lysis of 50% of parasites), CC_50_ (cytotoxic concentration necessary to cause lysis of 50% of cells), and CH_50_ (hemolytic concentration necessary to cause lysis of 50% of erythrocytes) values were calculated using sigmoid dose-response curves. The selective index (SI), which indicates the parasite toxicity as compared to the host, was calculated as the ratio between CC_50_ and IC_50_. The analyses were performed by using GraphPad Prism 8 (GraphPad Software, San Diego, CA, USA).

## 3. Results and Discussion

### 3.1. Synthesis of Compounds ***1**–**20***

The oxidative coupling of phenylpropanoids has been reported as the method of choice for the synthesis of DBNs [38]. This methodology allows for obtaining the structure core of DBNs in one single synthetic step in moderate yields (40–60%) [38]. Recently, Dias and co-workers achieved a considerable reduction in the reaction time (from 24 h to 4 h) by using acetonitrile as the reaction solvent, thus introducing an additional advantage to this methodology [20].

The synthesis of compounds **1***–***20** was designed based on the literature [14,23]. Pieters and co-workers also synthesized a series of DBNs using the Ag_2_O-promoted oxidative coupling of methyl esters of p-coumaric, ferulic, and caffeic acid. Further structure modifications (catalytic hydrogenation, reduction with LiAlH_4_, oxidation with DDQ, and alkylation with CH_3_I) resulted in a total of 19 DBNs and BNs, including compounds **1***–***3**, **12***–***15, 17**, **19**, and **20**, which were evaluated for their cytotoxicity to 60 humor tumor cell lines. Compound **3** displayed an average GI_50_ value (i.e., the molar drug concentration required for 50% growth inhibition) of 0.3 µM and inhibited mitosis at micromolar concentrations in cell culture through a relatively weak interaction at the colchicine binding site of tubulin purified from the bovine brain [23]. A few years later, Miert and co-workers carried out the same structure modifications reported by Pieters and co-workers, adding to acetylation with Ac_2_O, to a series of DBNs obtained from the oxidative coupling of different alkyl esters derived from p-coumaric, ferulic, and caffeic acid. Among the compounds tested for their antiparasitic activity, the DBN obtained from the oxidative coupling of caffeic acid and n-butyl ester displayed high activity against chloroquine-resistant *Plasmodium falciparum* (strain K1) (IC_50_ = 0.43 µg/mL), *Leishmania donovani* (IC_50_ values of 0.12 µg/mL and 0.19 µg/mL against axenic amastigotes and infected macrophage, respectively), and trypomastigote forms of *Trypanosoma brucei rhodiense* (IC_50_ = 0.29 µg/mL) [14]. The authors also evaluated the antitrypanosomal activity against trypomastigote forms of the *Trypanosoma cruzi* Tulahuen strain C2C4 containing a β-galactose (Lac-Z gene); however, none of the tested compounds displayed promising activity [14].

### 3.2. Antitrypanosomal Activity of Neolignans ***1**–**20*** against T. cruzi Amastigotes

Previous works have reported the in vitro antitrypanosomal activity of natural and synthetic DBNs and BNs against trypomastigote and epimastigote forms of *T. cruzi* [15,16,39]. Abe and co-workers (2006) described in vitro antitrypanosomal activity of the neolignans eupomatenoid-7 (a BN) and licarin A (a DBN) isolated from the root methanol extract of *Aristolochia taliscana* against epimastigote forms of the *T. cruzi* H6 strain (IC_50_ of 77 and 123 µM, respectively) [39]. Eupatemoid-3 (a BN), eupomatenoid-5 (a BN), eupomatenoid-6 (a BN), and conocarpan (a DBN), isolated from the leaf ethyl acetate of *Piper regnellii* var. *pallescens* (C. DC.) Yunck by Luize and co-workers, were active against epimastigote forms of the *T. cruzi* Y strain, displaying IC_50_ values of 26.3 µg/mL (90.9 µM), 7.0 µg/mL (23.8 µM), 7.5 µg/mL (28.4 µM), and 8.0 µg/mL (30.0 µM), respectively [40]. Pereira and co-workers (2011) synthesized (±)-licarin by oxidative coupling, isolated (+)-licarin and (-)-licarin, and tested their in vitro antitrypanosomal activity against trypomastigote forms of the *T. cruzi* Y strain. The authors demonstrated that (-)-licarin (IC_50_ = 23.46 µM) is more active than (+)-licarin (87.73 µM) and (±)-licarin (127.17 µM) [16]. However, data on the in vitro activity of these compounds against amastigote forms of *T. cruzi*, which are the intracellular forms found in the tissues of the vertebrate host [41], are still scarce [42].

The antitrypanosomal activity of the DBNs **1**–**6**, **9**–**20**, and BNs 7 and **8** against the amastigote forms of the *T. cruzi* Tulahuen lac-Z and Y strains is shown in Table 1.

First, the antitrypanosomal activity of compounds **1**–**20** was evaluated against Tulahuen lac-Z after 72 h. DBN **2** displayed the highest activity against amastigotes of the *T. cruzi* Tulahuen lac-Z strain (IC_50_ = 7.96 µM), followed by compounds **1** (IC_50_ = 11.87 µM), **3** (IC_50_ = 16.16 µM), and **4** (IC_50_ = 21.42 µM). On the other hand, compounds **5**–**20** showed IC_50_ values ranging from 31.58 to 300.7 µM. BZN (benznidazole), which was used as the reference compound (positive control), showed high activity (IC_50_ = 2.15 µM). Next, compounds **1**–**4**, which were the most active against amastigotes of *T. cruzi* Tulahuen lac-Z were evaluated against amastigotes of the *T cruzi* Y strain. DBN **1** displayed the highest activity (IC_50_ = 3.26 μM), with activity similar to BZN (IC_50_ = 3.56 μM), whereas DBNs **2**, **3**, and **4** exhibited IC_50_ values higher than 25 µM against the amastigotes of the *T. cruzi* Y strain (Table 1).

According to the literature, hit and lead compounds against intracellular amastigotes must display IC_50_ values lower than 10 µM [43]. In this context, compound **1** can be considered a hit compound in vitro, whereas compounds **2**, **3**, and **4** displayed promising activity against amastigote forms of *T. cruzi* Tulahuen lac-Z. The IC_50_ values obtained for **1**–**4** are lower as compared to those of eupomatenoid-5 (IC_50_ = 7 µg/mL or 23.78 µM), a benzofuran neolignan isolated from the leaves of *Piper regnelli* var. *pallescens* by Luize and co-workers [42].

Compounds **1**–**3** were synthesized directly from phenylpropanoids by oxidative coupling mediated by Ag_2_O, whereas compounds **4**–**20** were obtained from **1**–**3** (Scheme 1). The results shown in Table 2 evidenced that none of the derivatizations (reduction, oxidation, alkylation, acylation, and hydrolysis) performed in the structures of **1**–**3** to obtain **4**–**20** potentialized the in vitro antitrypanosomal activity. However, some preliminary structure-activity relationships can be suggested from these results. Reduction of the ester functionalities at C9 and C9′ to convert **1** and **2** into the primary alcohols **9** and **10** caused a 20-fold decrease in the activity. Hydrogenation of the double bond between C7′ and C8′ in **1** and **2** to produce **12** and **13** led to a 24-fold and 10-fold loss of the activity, respectively. Methylations (**1**→**16** and **2**→**17**) and acylations (at the hydroxy group at C4) also decreased the activity, but the decrease caused by these derivatizations is more discrete as compared to the ester and double-bond reductions. Apparently, the activity is also decreased by the presence of a double bond between C7 and C8 (**1**→**7**) and a carboxyl functionality at C9′ (**1**→**6**), as well as by the nature of the acyl group at C4 (conversion **1**→**4** led to a two-fold loss of the activity, whereas **1**→**11** cause a 6-fold loss of the activity). These results suggest that the ester function at C9 and C9′ and the double bond between C7′ and C8′ play a key role in the in vitro antitrypanosomal activity of DBNs against *T. cruzi*.

### 3.3. Cytotoxic and Hemolytic Activities of Neolignans ***1**–**4***

The cytotoxic activity of DBNs **1**–**4** and BZN in two non-infected mammalian cell lines (murine spleen and LLC-MK2) is shown in Table 2. All the CC_50_ values were found to be higher than the IC_50_ values in both cell lines. Compound **3** (CC_50_ = 30.76 µM) was the most cytotoxic in LLC-MK2 cells, followed by **4** (CC_50_ = 67.50 µM), **2** (CC_50_ = 67.59 µM), and **1** (CC_50_ = 70.86 µM). Compound **3** was also the most cytotoxic (CC_50_ < 1.95 µM) in murine spleen cells, followed by 2 (CC_50_ = 34.20 µM), 1 (CC_50_ = 49.87 µM), and 4 (CC_50_ = 51.09 µM).

According to the literature criteria, a hit compound should show selectivity index (SI) values higher than 10 to be considered safe for use in mammals [43,44]. Here, the SI values were calculated by dividing the CC_50_ values for the LLC-MK2 cell lines by the IC_50_ values for each *T. cruzi* strain at 72 h. Neolignan **1** displayed a low selectivity to *T. cruzi* Tulahuen lac-Z (SI = 5.96). On the other hand, this compound showed an interesting selectivity to *T. cruzi* Y strains (SI = 21.73). This selectivity to the *T. cruzi* Y strain was higher as compared to BZN (SI = 12.13), which was used as the positive control.

The hemolytic activity of compounds with antitrypanosomal activity is also a matter of concern due to the elevation of plasma hemoglobin with erythrocyte lysis, inducing deleterious effects mainly in the kidneys and cardiovascular system [45]. In this study, the hemolytic activity of DBNs **1**–**4**, which displayed the most potent in vitro antitrypanosomal activity, was evaluated after 30 min (Table 2). All the neolignans tested and BZN displayed CH_50_ higher than 100 µM.

### 3.4. Molecular Docking

The mechanism of action through which DBNs and BNs cause antitrypanosomal activity remains unknown. Recently, Maia and co-workers performed a virtual screening of 47 neolignans for their in vitro antitrypanosomal potential against three targets, the enzymes cruzain, trypanothione reductase, and sterol 14-alpha demethylase [46]. In the present study, the target of interest was tubulin, since it is a target of great importance for inhibiting the growth and death of the parasite [47]. Moreover, synthetic dihydrobenzofuran neolignans and their related benzofurans, which contain the well-known pharmacophore for inhibitors of tubulin polymerization binding at the colchicine site have been demonstrated to have antiprotozoal properties [14].

**Figure 1:**
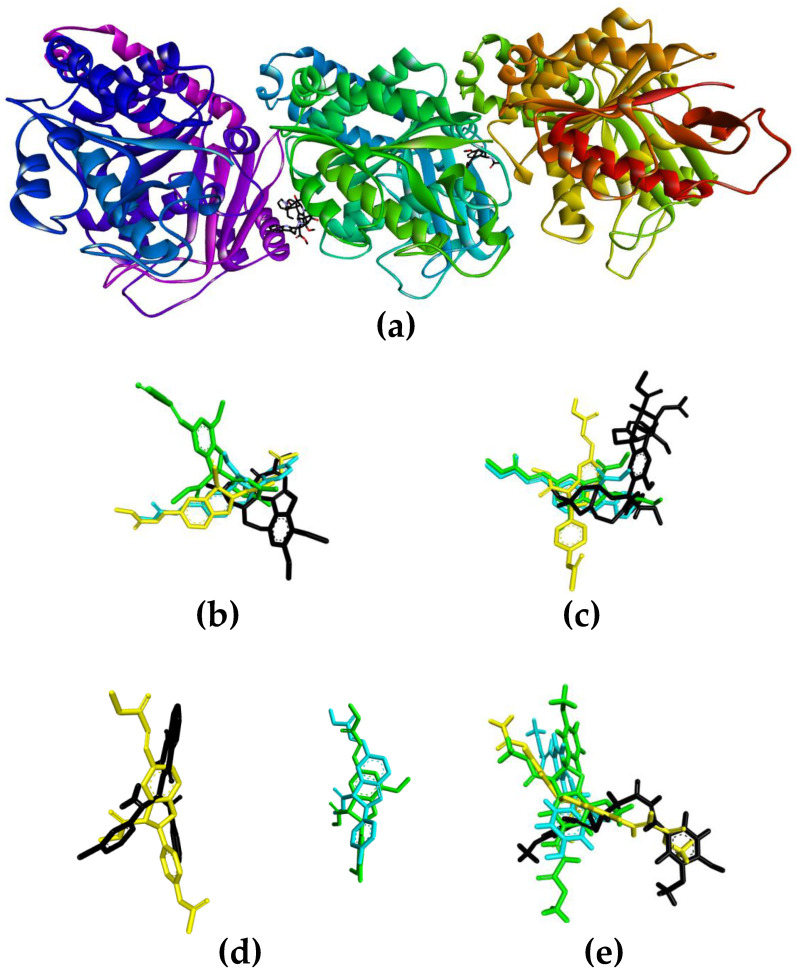
(**a**) α-β-α-tubulin structure, built by homology modeling. Overlays of DBNs **1** (blue), **2** (green), and **4** (yellow) with colchicine (**b**), vinblastine (**c**), imido-1,4-naphthoquinone derivative (**d**), and curcumin (**e**).

According to the literature, four binding sites capable of disturbing the dynamics of the tubulin-microtubule are described: the colchicine site, vinca (vinblastine), curcumin, and a fourth site where naphthoquinones and their derivatives bind to the protein [47,48,49]. Thus, in this work, the interactions between DBNs **1, 2**, and **4** and these four binding sites in *T. cruzi* tubulin were studied. To this end, an heterodimeral structure α-β-α for tubulin was constructed through homology modeling (Figure 1a) [34]. The negative Q-Mean score values for the α and β units were, respectively, −2.67 and −1.14, which are lower than their respective experimental structures and, therefore, were considered appropriate for docking [35]. Additionally, validation studies of the α1-β-α2 model showed that 97.8% of the protein’s amino acid residues are found in the most energetically favorable regions of the Rachamandran diagram and 2.2% in the energetically allowed regions. The re-docking values using colchicine and vinblastine presented, respectively, RMSD values of 0.56 Å and 0.94 Å, which indicated the good quality of the predictions made by molecular docking.

For more detailed analyses, the poses of compounds **1**, **2**, and **4** were compared to the poses of colchicine, vinblastine, imido-1,4-naphthoquinone derivative, and curcumin. In addition, the results were also compared with those previously reported in the literature [47,48,50,51,52]. The results indicated that, initially, there was an overlap between compounds **1**, **2**, and **4** and colchicine, vinblastine, imido-1,4-naphthoquinone derivative, and curcumin (Figure 1b–e), with the best overlap occurring with vinblastine through its vindoline group. This was confirmed by analyzing the interaction of the neolignan poses with the active protein site, in which the best results were observed at the vinca site (vinblastine). Hence, only the results regarding the poses on the vinca site will be described here.

Regarding **1** and **2**, hydrophobic interactions with β-Val175 and α-Cys353 residues are observed, as in the vinblastine pose (Figure 1b–e), in addition to the agreement with the literature regarding the hydrogen bonding with β- Val179 and hydrophobic interaction with α-Cys353 [52]. This indicates that these two neolignans behave similarly concerning the vinca site (vinblastine). For neolignan **4**, the interactions are similar to the vinblastine pose only in regards to the β fraction of the protein, with hydrophobic interactions between the neolignan and the β-Leu248 and β-Lys352 residues and hydrogen bonding with β-Asn329, which highlights that interactions with residues β-Lys352 and β-Asn329 are important at this site, as previously reported [52].

The results obtained for the docking of these neolignans suggest that the inhibition of tubulin occurs through interaction with the vinca site. These data also indicate that both **1** and **2** interact more strongly at the vinca site than **4**, as they interact with both residues important for protein inhibition located in the α and β subunits. However, the score values for the three structures are close (57.61, 61.98, and 56.63 for **1**, **2**, and **4**, respectively).

## 4. Conclusions

The neolignans evaluated in this study demonstrated promising in vitro activity on amastigote forms of *T. cruzi*, with emphasis on compounds **1**, **2**, and **4**. The results obtained from molecular docking indicated that **1**, **2**, and **4** are capable of destabilizing the dynamics of the tubulin-microtubule from the vinca site. The results indicated that the presence of the ester carbonyl at C9 and C9′ and the double bond between C7′ and C8′ play a key role in the antitrypanosomal activity. However, these preliminary structure-activity relationships must be further confirmed by QSAR (Quantitative Structure-Activity Relationships) studies, which are underway.

Compound **1** displayed an IC_50_ agaisnt the *T. cruzi* Y strain similar to that of BZN but with a two-fold increased selectivity to *T. cruzi* as compared to LLC-MK2 cells. These results demonstrated that DBN **1** can be considered a molecular prototype that could be used for the development of new antitrypanosomal drugs. However, further studies are needed to assess and elucidate the in vitro activity of this compound.

## Figures and Tables

**Table 1:** In vitro antitrypanosomal activity (IC_50_ values, in µM) of neolignans **1**–**20** against amastigotes of the *T. cruzi* Tulahuen lac-Z and Y strains after 72 h. 95% confidence limits are given in parentheses.

Compound	Tulahuen Lac-Z Strain	Y Strain
**1**	11.87 (9.50–14.83)	3.26 (1.56–6.80)
**2**	7.96 (6.36–9.97)	>25
**3**	16.16 (11.55–22.63)	>25
**4**	21.42 (17.55–26.14)	>25
**5**	31.58 (25.5–39.12)	ND
**6**	229 (219.7–238.8)	ND
**7**	75.0 (58.51–95.15)	ND
**8**	119.0 (112.2–126.2)	ND
**9**	246.6 (196.9–308.7)	ND
**10**	176.9 (117.8–265.8)	ND
**11**	70.05 (65.74–74.64)	ND
**12**	288.7 (282.8–292.7)	ND
**13**	79.26 (73.74–85.2)	ND
**14**	134.1 (92.65–194.2)	ND
**15**	148.8 (100.8–219.6)	ND
**16**	71.46 (67.58–75.57)	ND
**17**	72.27 (66.14–78.97)	ND
**18**	288.8 (279.3–298.6)	ND
**19**	300.7 (296.5–304.9)	ND
**20**	291.6 (286.4–297.0)	ND
BZN	2.15 (0.04–9.082)	3.56 (1.20–5.48)

IC_50_: the 50% Inhibitory Concentration; ND: not determined; BZN: benznidazole (positive control).

**Table 2:** In vitro cytotoxic (CC_50_) and hemolytic (CH_50_) activities of DBNs **1**–**4**, given in µM. 95% confidence limits are given in parentheses.

DBN	CC_50_		SI		CH_50_
	Murine Spleen (24 h)	LLC-MK2 (72 h)	Tulahuen Lac-Z Strain	Y Strain	Erythrocytes
**1**	49.87 (37.21–66.84)	70.86 (43.03–116.7)	5.96	21.73	>100
**2**	34.20 (21.74–53.79)	67.59 (46.11–99.09)	8.49	ND	>100
**3**	<1.95	30.76 (22.0–43.01)	1.90	ND	>100
**4**	51.09 (40.22–64.91)	67.50 (50.34–90.51)	3.15	ND	>100
BZN	1.81 (0.82–4.01)	43.21 (41.87–44.55)	20.05	12.13	>100

CC_50_: the 50% cytotoxic concentration in different line cells (Murine spleen cells and LLC-MK2 *Macaca mulatta* monkey epithelial kidney cells) after 24 and 72 h, respectively. Selectivity indexes (SI) were determined by dividing the CC_50_ of the LLC-MK2 by IC_50_ at 72 h of each *T. cruzi* strain; CH_50_: the 50% hemolytic concentration; ND: not determined; BZN: benznidazole (positive control).

## Data Availability

The data presented in this study are available on request from the corresponding author.

## Supplementary Materials

The following supporting information can be downloaded at: https://www.mdpi.com/article/10.3390/pharmaceutics15030754/s1, Table S1: NMR data of compounds **1**–**20**; Figures S1–S40: NMR spectra of compounds **1**–**20**; Figures S41–S60: GC-FID chromatograms of compounds **1**–**20**.
