# Supplementary material for: Exploring Synthetic Dihydrobenzofuran and Benzofuran Neolignans as Antiprotozoal Agents against Trypanosoma cruzi
Source: Pharmaceutics. 2023 Feb 24;15(3):754. doi: 10.3390/pharmaceutics15030754 (PMC10052957; doi:10.3390/pharmaceutics15030754)
Supplement: Supplementary file 1 [file pharmaceutics-15-00754-s001.zip › pharmaceutics-2190287-supplementary.pdf]

Supplementary Material

Exploring synthetic dihydrobenzofuran and benzofuran neolignans as antiprotozoal agents against *Trypanosoma cruzi*

Mariana C. Pagotti [1], Herbert J. Dias [2,3], Ana Carolina B. B. Candido [1], Thaís A. S. Oliveira, [2] Alexandre Borges [1,4], Nicoli D. Oliveira [5], Carla D. Lopes [6], Renato P. Orenha [1], Renato L. T. Parreira [1], Antônio E. M. Crotti [2,*] and Lizandra G. Magalhães [1,5*]

[1] Research Group on Natural Products, Center for Research in Sciences and Technology, University of Franca, Franca, São Paulo
[2] Department of Chemistry, Faculty of Philosophy, Sciences and Letters, University of São Paulo, Ribeirão Preto, São Paulo, Brazil
[3] Goiano Federal Institute of Education, Science, and Technology, Campus Urutaí, Urutaí, Goiás, Brazil
[4] Faculty of Medicine, University Center of Santa Fe do Sul, Santa Fé do Sul, São Paulo, Brazil
[5] Animal Science Post Graduation, University of Franca, Franca, São Paulo, Brazil
[6] Department of Biochemistry and Immunology, Medical School of Ribeirão Preto, University of São Paulo, Ribeirão Preto, São Paulo, Brazil

* Correspondence: lizandra.magalhaes@unifran.edu.br (L.G.M.) and millercrotti@ffclrp.usp.br (A.E.M.C.)

Table S1. ^{1}H (400 MHz) and ^{13}C NMR (100 MHz) data of compounds **1-20**.

**Compound 1**
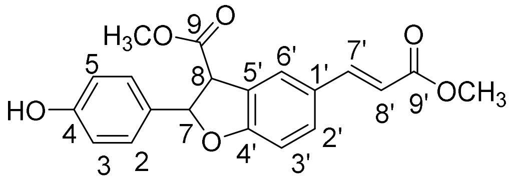
**(±)-*trans*-dehydrodicoumarate dimethyl ester** (**1**). Yellow powder, m.p. 105-107 °C, 36% yield. NMR ^{1}H (400 MHz, *Acetone-d$_6$*, **S1**): δ 3.73 (3H, s, H10'), 3.81 (3H, s, H10), 4.40 (1H, d, $J_{8,7}$ = 7.3 Hz, H8), 6.03 (1H, d, $J_{7,8}$ = 7.3 Hz, H7), 6.41 (1H, d, $J_{8',7'}$ = 16.0 Hz, H8'), 6,87 (2H, dd, $J_{3,5}$ = 1,8 Hz, $J_{3,2}$ = 6.8 Hz, H3=H5), 6,91 (1H, d, $J_{3',2'}$ = 8.2 Hz, H3'), 7,28 (2H, dd, $J_{2,6}$ = 1.8 Hz, $J_{2',3'}$ = 6.8 Hz, H2=H6), 7.60 (1H, dd, $J_{2',6'}$ = 1.5 Hz, $J_{2',3'}$ = 8.1 Hz, H2'), 7.65 (1H, d, $J_{7',8'}$ = 16.0 Hz, H7'), 7.72 (1H, bs, H6'). ^{13}C (100 MHz, *Acetone-d$_6$*, **S2**): δ 52.0 , 53.4 , 56.0 , 88.2 , 111.2 , 116.5 , 116.9 , 126.5 , 126.9 , 128.9 , 128.9 , 128.9 , 131.3 , 132.1, 145.5 , 159.1 , 162.6 , 168.2 , 172.0.
**Compound 2**

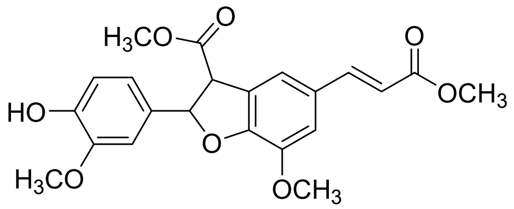

(±)-*trans*-dehydrodiferulate dimethyl ester (**2**). Yellow oil, 43% yield. NMR ^{1}H (400 MHz, *Acetone-d$_6$,* **S3**): δ 3.73 (3H, s, H10) , 3.81 (3H, s, H10′) , 3.84 (3H, s, H11) , 3.92 (3H, s, H11′) , 4.47 (1H, d, $J_{3,2}$ = 8.1 Hz, H8) , 6.04 (1H, d, $J_{2,3}$ = 8.1 Hz, H7) , 6.44 (1H, d, $J_{9,8}$ = 16.1 Hz, H8′) , 6.84 (1H, d, $J_{5',6'}$=8.1 Hz, H5) , 6.92 (1H, dd, $J_{6',5'}$=8.1 Hz, $J_{6',2'}$=1.7 Hz, H6) , 7.10 (1H, d, $J_{2',6'}$=1.7 Hz, H2) , 7.29 (1H, sl , H7′) , 7.33 (1H, sl , H6′) , 7.63 (1H, d, $J_{8,9}$ = 16.1 Hz, H7′). ^{13}C (100 MHz, *Acetone-d$_6$,* **S4**): δ 52.0, 53.5; 56.4, 56.8; 56.9 ; 88.8 ; 111.2, 113,9 , 116.3 , 116.7 , 119.4 , 120.7 , 127.8 , 129.9 , 132.5 , 145.9 , 146.3 , 148.5 , 149.0 , 151.5 , 168.2 , 172.1.

Compound 3

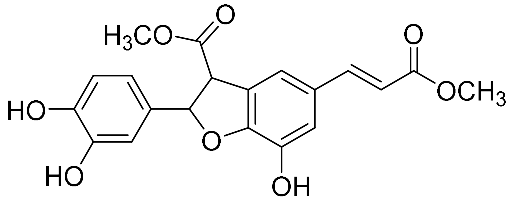

(±)-*trans*-dehydrodicafeoate dimethyl ester (3). Yellow oil, 12% yield. NMR ^{1}H (400 MHz, *Acetone-d$_6$*, **S5**): δ 3.73 (3H, s, H10′); 3.81 (3H, s, H10); 4.37 (1H, d, *J*= 7.3 Hz, H8); 5.98 (1H, d, *J*= 7.3 Hz, H7); 6.35 (1H, d, *J*= 16.1 Hz; H8′); 6.80 (1H, dd, *J*= 1.6 Hz; 8.2 Hz; H6); 6.85 (1H, d, *J*= 8.2 Hz; H5); 6.90 (1H, d, *J*= 1.6 Hz; H2); 7.15 (1H, s, H6′); 7.21 (1H, s, H2′); 7.58 (1H, d, *J*= 16.1 Hz; H7′); 8.04 (3-OH); 8.07 (3′-OH); 8.50 (4-OH). ^{13}C (100 MHz, *Acetone-d$_6$*, **S6**): δ 51.7; 53.1; 56.5; 88.1; 114.1; 116.2; 116.3; 117.6; 117.9; 118.9; 127.6; 129.6; 132.9; 142.8; 145.6; 146.3; 146.6; 150.3; 167.9; 171.8.

Compound 4

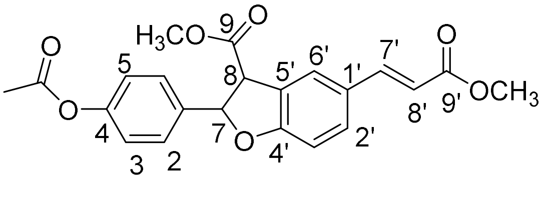

(±)-4-O-acetyl-*trans*-dehydrodicoumarate dimethyl ester (**4**). Yellow powder, m.p. 103-105 °C, 82% yield. NMR ^{1}H (400 MHz, *Acetone-d$_6$*, **S7**): δ 2.26 (3H, s, H12) , 3.73 (3H, s, H10′) , 3.83 (3H, s, H10) , 4.44 (1H, d, $J_{8,7}$ = 7.2 Hz, H8) , 6.15 (1H, d, $J_{7,8}$ = 7.2 Hz, H7) , 6.42 (1H, d, $J_{8',7'}$ = 15.9 Hz, H8′), 6.97 (1H, d, $J_{3',2'}$ = 8.4 Hz) , 7.16 (2H, dd, $J_{3,5}$ = 1.9 Hz; $J_{3,2}$ = 6.7 Hz, H3=H5) , 7.49 (2H, dd, $J_{2,6}$ = 1.7 Hz; $J_{2,3}$ = 6.7 Hz, H2=H6) , 7.63 (1H, dd, $J_{2',6'}$ = 1.7 Hz; $J_{2',3'}$ =8.3 Hz, H2′) , 7.65 (1H,

d, $J_{7',8'}$ = 15.9 Hz) , 7.74 (1H, sl). ^{13}C (100 MHz, *Acetone-d$_6$*, **S8**): δ 21.5 , 52.2 , 53.7 , 56.4 , 87.6 , 111.5 , 116.9 , 123.6 , 126.9 , 127.1 , 128.5 , 128.5 , 128.5 , 129.6 , 132.3 , 139.2 , 145.6 , 152.7 , 162.7 , 168.3 , 170.2 , 172.1.

Compound 5

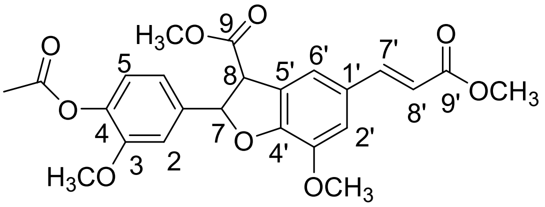

(±)-4-O-acetyl-*trans*-dehydrodiferulate dimethyl ester (5). Brown oil, 96% yield. NMR ^{1}H (400 MHz, *Acetone-d$_6$*, **S9**): δ 2.24 (3H, s, H12) , 3.73 (3H, s, H10′) , 3.82 (3H, s, H10) , 3.83 (3H, s, H12) , 3.94 (3H, s, H11′) , 4.50 (1H, d, $J_{8,7}$ = 7.5 Hz, H8) , 6.14 (1H, d, $J_{7,8}$ = 7.5 Hz, H7) , 6.45 (1H, d, $J_{8',7'}$ = 16.0 Hz, H8′, 7.03 (1H, d, $J_{5,6}$ = 8.2, H5) , 7.09 (1H, dd, $J_{6,5}$ = 8.2 Hz; $J_{6,2}$ = 1.7, H6) , 7.25 (1H, d, $J_{2,6}$ = 1.7 Hz, H2) , 7.31 (1H, sl, H6′) , 7.35 (1H, sl, H2′) , 7.63 (1H, d, $J_{7',8'}$ = 16,0 Hz, H7′). ^{13}C (100 MHz, *Acetone-d$_6$*, **S10**): δ 21.0 , 52.1 , 53.7 , 56.7 , 56.9 , 57.2 , 88.2 , 111.9 , 114.3 , 117.1 , 119.6 , 124.5 , 127.7 , 130.3 , 140.3 , 141.7 , 145.9 , 146.5 , 151.5 , 153.2 , 168.3 , 169.5 , 172.1.

Compound 6

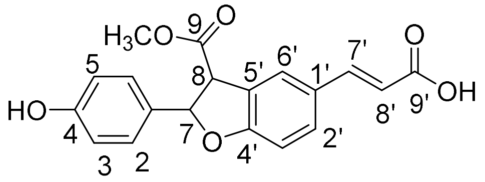

(±)-*trans*-dehydrodicoumarate methyl acid (**6**). Yellow oil, 38% yield. NMR ^{1}H (400 MHz, *Acetone-d$_6$*, **S11**): δ 3.81 (3H, s, H10, 4.40 (1H, d, $J_{8,7}$ = 7.3 Hz, H8), 6.03 (1H, d, $J_{7,8}$ = 7.3 Hz, H7), 6.39 (1H, d, $J_{8',7'}$ = 16.0 Hz, H8′), 6,87 (2H, dd, $J_{3,5}$ = 2.0 Hz, $J_{3,2}$ = 6.6 Hz, H3=H5), 6,92 (1H, d, $J_{3',2'}$ = 8.3 Hz, H3′), 7,29 (2H, dd, $J_{2,6}$ = 1.8 Hz, $J_{2',3'}$ =6.6 Hz, H2=H6), 7.60 (1H, dd, $J_{2',6'}$ = 1.8 Hz, $J_{2',3'}$ = 8.3 Hz, H2′), 7.65 (1H, d, $J_{7',8'}$ = 16.0 Hz, H7′), 7.72 (1H, d, $J_{6',2'}$ = 1.8 Hz, H6′). ^{13}C (100 MHz, *Acetone-d$_6$*, **S12**): δ 53.0, 55.7 , 87.8 , 110.8 , 116.3 , 116.7 , 126.2, 126.2, 126.9 , 128.6 , 128.9 , 128.9 , 131.7 , 132.10 , 145.3 , 158.8 , 162.2 , 168.1 , 171.8.

Compound 7

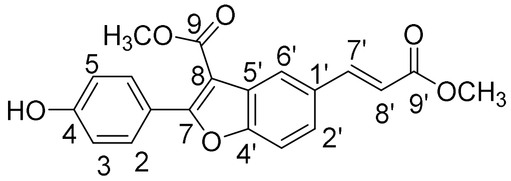

(±)-7,8-dehydro-*trans*-dehydrodicoumarate dimethyl ester (7). Orange powder, m. p. 204-206 ºC, 74% yield. NMR ^{1}H (400 MHz, *Acetone-d$_6$*, **S13**): δ 3.77 (3H, s, H10′), 3.96 (3H, s, H10),

6.57 (1H, d, J = 15.9 Hz, H8′), 7.00 (2H, d, J = 8.8 Hz, H2=H6), 7.62 (1H, d, $J_{3',2'}$ = 8.6 Hz), 7.74 (1H, dd, $J_{2',3'}$ = 8.6 Hz. $J_{2',6'}$ = 1.6) Hz, 7.82 (1H, d, J = 15.9 Hz, H7′), 8.03 (2H, d, J = 8.8 Hz, H3=H5), 8.29 (1H, d, J = 1.6 Hz, H6′), 9.14 (1H, s, OH). ^{13}C (100 MHz, *Acetone-d₆*, **S14**): δ 51.8 , 52.0 , 112.5 , 116.0 , 118.1 , 121.3 , 123.8 , 125.9 , 129.0 , 131.0 , 131.7 , 132.3 , 133.4 , 145.6 , 155.3 , 160.8 , 166.6 , 167.6.

Compound 8

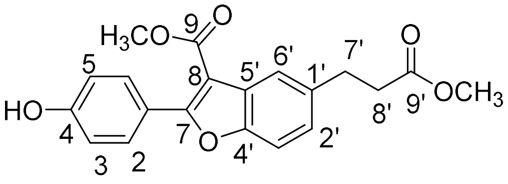

(±)-7,8-dehydro-7′,8′-dihydro-*trans*-dehydrodicoumarate dimethyl ester (8). Yellow oil, 94% yield. NMR ^{1}H (400 MHz, *Acetone-d_6*, **S15**): δ 2.69 (2H, t, J = 7.7 Hz, H8′), 3.05 (2H, t, J = 7.7 Hz, H7′), 3.63 (3H, s, H10′), 3.91 (3H, s, H10), 6.99 (2H, d, J = 8.8 Hz, H2 = H6), 7.27 (1H, dd, J = 1.6 and 8.3 Hz, H2′), 7.49 (1H, d, J = 8.3 Hz, H3′), 7.90 (1H, d, J = 1.6 Hz, H6′), 7.98 (2H, d, J = 8.8 Hz, H2 = H6). ^{13}C (100 MHz, *Acetone-d_6*, **S16**): δ 31.7 , 36.8 , 51.7 , 51.8 , 107.9 , 111.6 , 115.9 , 121.8 , 122.7 , 126.5 , 128.4 , 132.1 , 137.7 , 153.1 , 160.5 , 162.2 , 165.0 , 173.5.

Compound 9

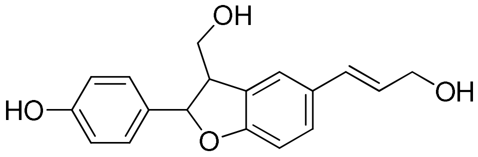

(±)-*trans*-dehydrodicoumarate-diol (9). White powder, m.p. 139-140°C, 62% yield. NMR ^{1}H (400 MHz, *Acetone-d_6*, **S17**: δ 3.49 (1H, q, J=6.0, H8); 3.85 (2H, m, H9); 3.85 (OH, m, H9); 4.20 (2H, m, H9′); 4.20 (OH, M, H9′); 5.55 (1H, d, J=6.0 Hz, H7); 6.22 (1H, dt, J=15.8 and 5.6 Hz, H7′); 6.55 (1H, d, J= 15.8 Hz, H7′); 6.74 (1H, d, J=8.3 Hz, H3′); 6.82 (2H, d, J=8.5 Hz, H2=H6); 7.22 (1H, d, J=8.3 Hz, H2′); 7.24 (2H, d, J=8.5 Hz, H2=H6); 7.39 (1H, s, H6′); 8.40 (1H, s, OH). ^{13}C (100 MHz, *Acetone-d_6*, **S18**): δ 54.5; 63.5; 64.9; 88.1; 109.7; 116.0; 116.1; 123.5;128.1; 128.1; 128.1; 128.2; 129.6; 130.4; 131.1; 134.1;158.1; 160.6.

Compound 10

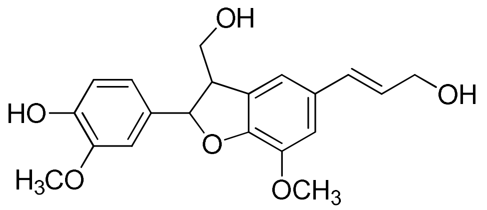

(±)-*trans*-dehydrodiferuloate-diol (10). Brown oil, 60% yield. NMR ^{1}H (400 MHz, *Acetone-d_6*, **S19**): δ 3.54 (1H, ddd, *J*=6.8, 5.5, and 3.9 Hz, H8); 3.82 (3H, s, H10); 3.84 (1H, t, *J*=5.5 Hz, H9b); 3.86(3H, s, H10'); 3.88(1H, t, *J*=5.5 Hz, H9a); 4.20 (2H, d, *J*=5.5 Hz, H9'); 5.57 (1H, d, *J*=6.8 Hz, H7); 6.24 (1H, dt, *J*=15.8 and 5.5; H8'); 6.53 (1H, d, *J*=15.8 Hz, H7'); 6.81 (1H, d, *J*=8.1 Hz, H5); 6.89 (1H, dd, *J*=2.1 and 8.1 Hz; H5); 6.95 (1H, s, H2'); 6.98 (1H, s, H6'); 7.04 (1H, d, *J*=2.1 Hz, H2). ^{13}C (100 MHz, *Acetone-d_6*, **S20**): δ 54.9; 56.5; 56.6; 63.5; 64.7; 88.7; 110.8; 112.1; 115.9; 116.3; 119.7; 128.6; 130.6; 130.7; 132.1; 134.6; 145.3; 147.5; 148.6; 149.2.

Compound 11

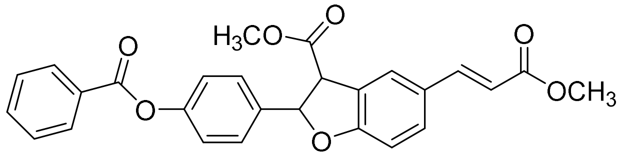

(±)-4-O-benzoyl-*trans*-dehydrodicoumarate dimethyl ester (11) White powder, m.p. 109-111 °C, 95% yield. NMR ^{1}H (400 MHz, CDCl$_3$, **S21**): δ 3.82 (3H, s, H10') , 3.82, (3H, s, H10) , 3.87 (3H, s, H11), 3.95 (3H, s, H11'), 4.38 (1H, d, $J_{8,7}$ = 8.0 Hz, H8) , 6.23 (1H, d, $J_{7,8}$ = 8.0 Hz, H7) , 6.34 (1H, d, $J_{8',7'}$ = 15.9 Hz, H8'), 7.05 (1H, d, $J_{5,6}$ = 8.1 Hz, H5), 7.07 (2H, m , H2=H2'), 7.16 (1H, d, $J_{6,5}$ = 8.1 Hz, H6), 7.22 (1H, sl, H6'), 7.51 (3H, m, H15, H16, H17), 7.62 (2H, m, H14, H18), 7.67 (1H, d, $J_{7',8'}$ = 15.9 Hz, H7'). ^{13}C (100 MHz, CDCl$_3$, **S22**): 51.5, 52.7, 54.8, 54.9, 86.6, 110.1, 115.2, 115.7, 115.9, 116.1, 124.9, 125.2, 127.1, 127.3, 127.7, 128.3, 129.9, 130.0, 130.6, 130.8, 133.1, 141.5, 144.5, 145.5, 157.2, 161.1, 167.7, 169.5, 170.8.

Compound 12

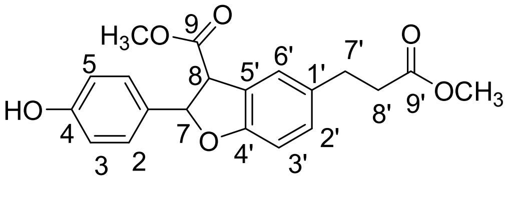

(±)-7',8'-dihydro-*trans*-dehydrodicoumarate dimethyl ester (12). White powder, m. p. 108-110 °C, 95% yield. NMR ^{1}H (400 MHz, *Acetone-d_6*, **S23**): δ 2.59 (2H, dt, *J* = 7.8 and 2.9 Hz, H7'), 2.86 (2H, d, *J* = 7.8 Hz, H8'), 3.61 (3H, s, H10'), 3.79 (3H, s, H10), 4.29 (1H, d, *J* = 7.8 Hz, H8), 5.95 (1H, d, *J* = 7.8 Hz, H7), 6.75 (1H, d, *J* = 8.0 Hz, H3'), 6.85 (2H, d, *J* = 8.6, H2 = H6), 7.12 (1H, dd, *J*

= 8.0 and 2.9 Hz, H2′), 7.23 (1H, s, H6′), 7.27 (2H, d, *J* = 8.6 Hz, H3 = H5), 8.52 (1H, s, OH). ^{13}C (100 MHz, *Acetone-d$_6$*, **S24**): *δ* 31.4 , 36.7 , 51.7 , 52.9 , 56.4 , 87.0 , 110.2 , 116.4, 125.7 , 126.0 , 128.4 , 130.4, 132.6, 134.4 , 158.6 , 158.9 , 172.1 , 173.5

Compound 13

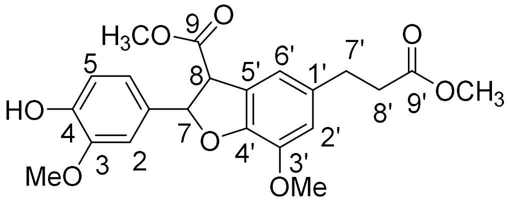

(±)-7′,8′-dihydro-*trans*-dehydrodiferulate dimethyl ester (13). m.p. 118-120 °C, white powder, 88% yield. NMR ^{1}H (400 MHz, *Acetone-d$_6$*, **S25**): *δ* 2.59 (2H, dt, *J* = 7.8 and 2.6 hz, H7′), 2.85 (2H, d, *J* = 7.8 Hz, H8′), 3.62 (3H, s, H10′), 3.78 (3H, s, H10), 3.83 (6H, s, H11 = H11′), 4.35 (1H, d, *J* = 8.3 Hz, H8), 5.94 (1H, d, *J* = 8.3 Hz), 6.83 (1H, d, *J* = 8.3 Hz, H5), 6.84 (2H, m, H2′ = H6′), 6.89 (1H, dd, J_6 = 8.3 and 1.8 Hz, H6), 7.07 (1H, d, $J_{2,6}$ = 1.8 Hz, H2). ^{13}C (100 MHz, *Acetone-d$_6$*, **S26**): *δ* 31.5 , 36.7 , 51.7 , 52.9 , 56.5 , 56.6 , 56.7 , 87.6 , 110.8 , 114.8 , 115.9 , 117.5 , 120.1 , 126.8 , 132.7 , 134.0 , 135.4 , 145.4 , 147.5 , 147.9 , 148.7, 172.1, 173.6.

Compound 14

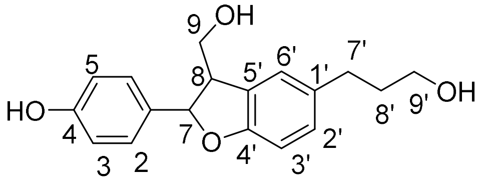

(±)-7′,8′-dihydro-*trans*-dehydrocoumarate-9,9′-diol (14). White powder, m.p. 120-121°C, 59% yield. IR NMR ^{1}H (400 MHz, *Acetone-d$_6$*, **S51**): *δ* 1.78 (2H, dddd, *J*= 5.5, 6.6, 7.3, and 9.3 Hz, H8′); 2.62 (2H, dd, *J*= 6.6 and 9.3 Hz, H7′); 3.46 (1H, dt, *J*= 6.0 and 6.5 Hz, H8); 3.57 (2H, t, *J* = 6.5 Hz, H9); 3.79 (1H, dd, *J*= 10.7 and 7.3 Hz, H9a′); 3.86 (1H, dd, *J*= 10.7 and 7.3 Hz, H9b′); 5.51 (1H, d, *J*= 6.0 Hz, H7); 6.70 (1H, d, *J*= 8.1 Hz, H3′); 6.82 (2H, d, *J*= 8.6 Hz, H2=H6); 7.00 (1H, dd, *J*= 1.1 Hz; 8.1, H2′); 7.13 (1H, d, *J*= 1.1 Hz, H6′); 7.23 (2H, d, *J*= 8.6 Hz, H3=H5). ^{13}C (100 MHz, *Acetone-d$_6$*, **S52**): *δ* 32.7; 36.3; 55.1; 62.2; 65.3, 88.0, 109.7, 116.4, 116.4, 126.0, 128.3, 128.3, 129.2, 129.5, 134.7, 135.5, 158.3, 159.4.

Compound 15

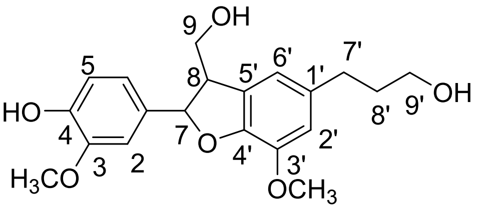

(±)-7′,8′-dihydro-*trans*-dehydrodiferulate-9,9′-diol (15). Colorless oil, 52% yield. NMR ^{1}H (400 MHz, *Acetone-d$_6$*, **S29**): δ 1.79 (2H, dt, *J* = 6.5 and 6.0 Hz, H8′); 2.62 (2H, t, *J*=6.5 Hz, H7′); 3.51(1H, t, *J*= 6.5 Hz, H8); 3.65 (2H; t, *J*=6.5 Hz, H9′); 3.80 (2H, dd, *J*= 10.8 and 6.0 Hz; H9b); 3.82 (3H, s, H10′); 3.84 (3H, s, H10); 3.87 (2H, dd, *J*=10.8 and 6.0 Hz; H9a); 5.53 (7H, d, *J*=6.7 Hz, H7); 6.72(1H, bs, H2′); 6.75 (1H; bs, H6′); 6.81 (1H, d, *J*=8.0 Hz, H5); 6.89 (1H, dd, *J*=8.0 and 1.8 Hz, H6); 7.04 (1H, d, *J*=1.8 Hz, H2). ^{13}C (100 MHz, *Acetone-d$_6$*, **S30**): δ 33.3; 36.5; 54.2; 56.9; 57.1; 62.4; 65.3; 88.8; 111.1; 114.64; 116.2; 118.2; 120.1; 130.6; 136.9; 145.5; 147.6; 148.9.

Compound 16

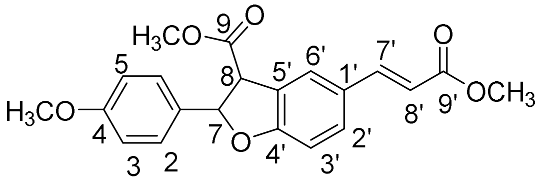

(±)-4-O-methyl-*trans*-dehydrodicoumarate dimethyl ester (**16**). Yellow oil, 22% yield. NMR ^{1}H (400 MHz, *Acetone-d$_6$*, **S31**): δ 3.73 (3H, s, H10′), 3.80 (3H, s, H10), 3.83 (3H, s, H11), 4.40 (1H, d, $J_{8,7}$ = 7.8 Hz, H8), 6.07 (1H, d, $J_{7,8}$ = 7.2 Hz, H7), 6.42 (1H, d, $J_{8',7'}$ = 16.0 Hz, H8′), 6.92 (1H, d, $J_{3',2'}$ = 8.3 Hz, H3′), 6.96 (2H, dd, $J_{3,5}$ = 2.0 Hz, $J_{3,2}$ = 6.7 Hz, H3=H5), 7.38 (2H, dd, $J_{2,6}$ = 2.0 Hz, $J_{2',3'}$ =.6.7 Hz, H2=H6), 7.61 (1H, dd, $J_{2',6'}$ = 1.8 Hz, $J_{2',3'}$ = 8.3 Hz, H2′), 7.66 (1H, d, $J_{7',8'}$ = 16.0 Hz, H7′), 7.72 (1H, d, $J_{6',2'}$= 1.8 Hz, H6′). ^{13}C (100 MHz, *Acetone-d$_6$*, **S32**): δ 51.7 , 53.1 , 57.7 , 60.6 , 87.6 , 110.9 , 115.0 , 115.0 , 116.2 , 126.2 , 126.8 , 128.5, 128.5 , 128.9 , 131.8 , 133.1 , 145.2 , 161.1 , 162.2 , 167.8 , 171.7.

Compound 17

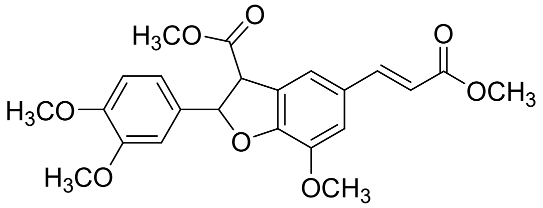

(±)-4-O-methyl-*trans*-dehydrodiferulate dimethyl ester (**17**). White powder, m.p. 96-98 ºC, 18% yield. NMR ^{1}H (400 MHz, *Acetone-d$_6$*, **S33**): δ 3.73 (3H, s, H10′) , 3.80, (3H, s, H10) , 3.81 (3H, s, H11), 3.81 (3H, s, H12), 3.93 (3H, s, H11′), 4.47 (1H, d, $J_{8,7}$ = 7.8 Hz, H8) , 6.06 (1H, d, $J_{7,8}$ = 7.8 Hz, H7) , 6,44 (1H, d, $J_{8',7'}$ = 15.9 Hz, H8′), 6.95 (1H, d, $J_{5,6}$ = 8.3 Hz, H5) , 6.99 (1H, dd, $J_{6,5}$ = 8.3 Hz, $J_{6,2}$ = 1.8 Hz, H6), 7.09 (1H, d, $J_{2,6}$ = 1.8 Hz, H2), 7.29 (1H, sl, H6′), 7.34 (1H, sl, H2′) , 7.63 (1H, d, $J_{7',8'}$ = 15.9 Hz, H7′). ^{13}C (100 MHz, *Acetone-d$_6$*, **S34**): 51.6, 53.0, 56.0, 56.1, 56.2, 56.5, 88.2, 110.9, 112.6, 113.5, 116.3, 119.0, 119.7, 127.3, 129.5, 133.1, 145.4, 145.9, 150.6, 150.8, 151.0, 167.8, 171.6.

Compound 18

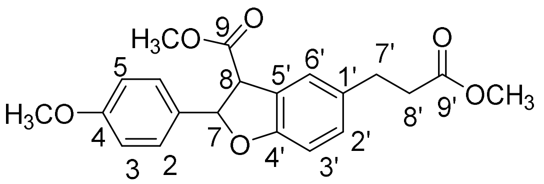

(±)-4-O-methyl-*trans*-dihydrodicoumarate dimethyl ester (**18**). Yellow oil, 90% yield. NMR ^{1}H (400 MHz, $CDCl_3$, **S35**): δ 2.61 (2H, t, $J_{7',8'}$ = 7.6 Hz, H7′) , 2.92 (2H, t, $J_{8',7'}$ = 7.6 Hz, H8′) , 3.69 (3H, s, H10′) , 3.81 (3H, s, H10) , 3.83 (3H, s, H11) , 4.26 (1H, d, $J_{8,7}$ = 7.8 Hz, H7) , 6.03 (1H, d, $J_{8,7}$ = 7.8 Hz, H8) , 6.82 (1H, d, $J_{3',2'}$ = 8.3 Hz, H3′) , 6.90 (2H, d, $J_{3=5,\ 2=6}$ = 8.6 Hz, H2=H6) , 7.07 (1H, dd, $J_{2',3'}$ = 8.1 Hz Hz, $J_{6',2'}$ = 1.6, H2′) , 7.18 (1H, d, $J_{6',2'}$ = 1.6 Hz, H6′) , 7.33 (2H, d, $J_{2=6,\ 3=5}$ = 8.6 Hz, H3=H5). ^{13}C (100 MHz, $CDCl_3$, **S36**): δ 30.8, 36.5, 51.9, 55.6, 56.0, 86.1, 110.0, 114.5, 124.5, 125.2, 127.6, 129.8, 132.9, 133.5, 158.2, 160.1, 171.7, 173.7.

Compound 19

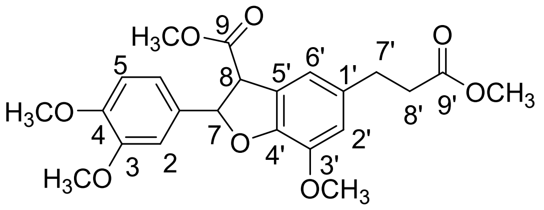

(±)-7′,8′-dihydro-4-O-methyl-*trans*-dehydrodiferulate dimethyl ester (**19**). White powder, m.p. 108-110 °C, 91% yield. NMR ^{1}H (400 MHz, *Acetone-d*$_6$, **S37**): δ 2.59 (2H, t, $J_{7',8'}$ = 7.8 Hz, H7′) , 2.85 (2H, d, $J_{8',7'}$ = 7.8 Hz, H8′) , 3.62 (3H, s, H10′) , 3.78 (3H, s, H10), 3.80 (3H, s, H11′), 3.80 (3H, s, H11), 3.84 (1H, s, H8), 4.35 (1H, d, $J_{7,8}$ = 8.3 Hz), 5.94 (1H, d, $J_{8,7}$ = 8.3 Hz), 6.84 (1H, s, H6′), 6.84, (1H, s, H2′), 6.93 (1H, dd, $J_{5,6}$ = 8.3 Hz, $J_{5,2}$ = 1.8 Hz), 6.99 (1H, d, $J_{8,7}$ = 1.8 Hz). ^{13}C (100 MHz, *Acetone-d*$_6$, **S38**): δ 31.4 , 36.6 , 52.8 , 56.1 , 56.4 , 56.6 , 87.3 , 110.8 , 112.6 , 114.4 , 117.4 , 119.5 , 126.6 , 133.7 , 135.4 , 145.3 , 147.3 , 150.6 , 150.6 , 172.0 , 173.5.

Compound 20

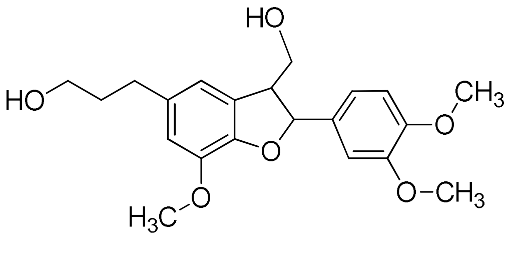

3-[2-(3,4-Dimethoxyphenyl)-3-hydroxymethyl-7-methoxy-2,3-dihydro-1-benzofuran-5-yl]propan-1-ol (**18**). Colorless oil, 49% yield. NMR ^{1}H (500 MHz, $CDCl_3$, **S39**): δ 1.89 (2H, dt, $J_{8',7'}$=6.5 Hz, $J_{8',9'}$=6.4 Hz, H8′), 2.68 (2H, t, $J_{7',8'}$=6.5 Hz, H7′), 3.62, (1H, m, H8), 3.70 (2H, t, $J_{9',8'}$=6.4 Hz, H9′), 3.86 (3H, s, H10), 3.87 (3H, s, H11), 3.89 (3H, s, H10′), 3.95 (2H, m, H9), 5.57 (1H, d, $J_{7,8}$ = 7.4 Hz, H7), 6.69 (2H, bs, H2′=H6′), 6.85 (1H, d, $J_{6,5}$ = 8.8 Hz, H6), 6.96 (1H, bs, H2), 6.97 (1H,

d, $J_{5,6}$ = 8.8 Hz, H5). ^{13}C (100 MHz, $CDCl_3$, **S40**): δ 32.0, 34.6, 53.8, 56.0, 56.0, 56.1, 62.3, 64.1, 87.8, 109.6, 111.3, 112.8, 116.1, 118.7, 127.8, 133.9, 144.3, 146.7, 149.3, 194.1.

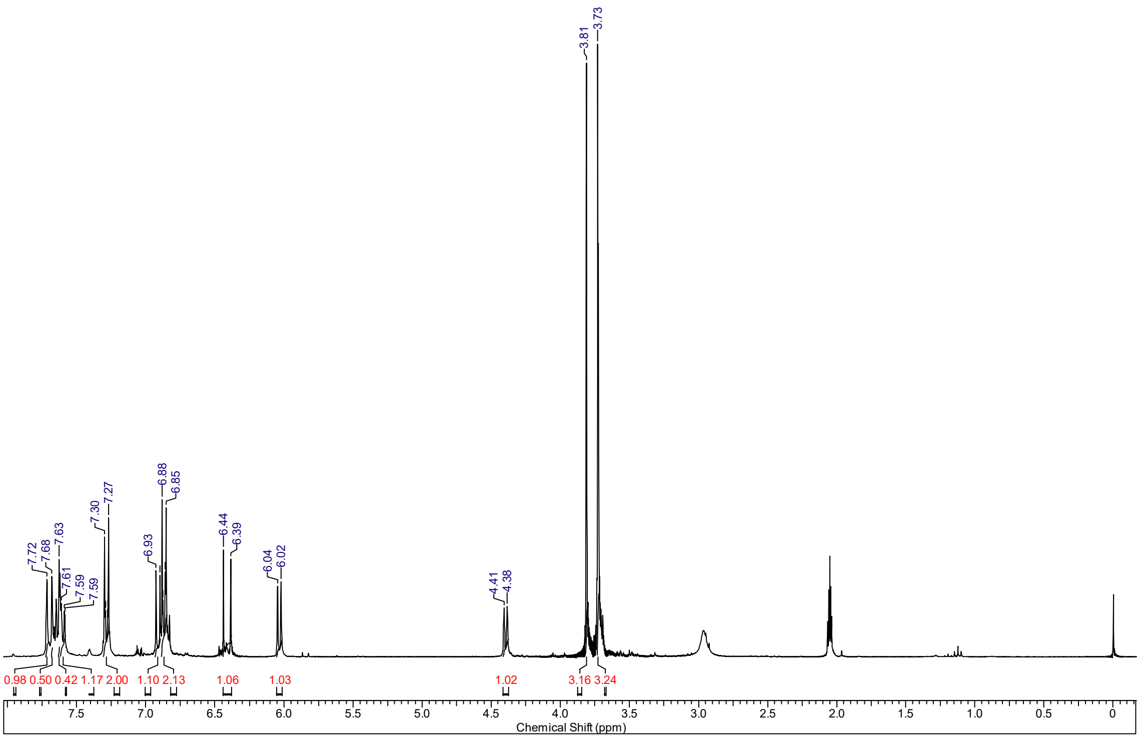

Figure S1. ^{1}H NMR spectra of compound **1** (*Acetone-d_6*, 400 MHz, TMS).

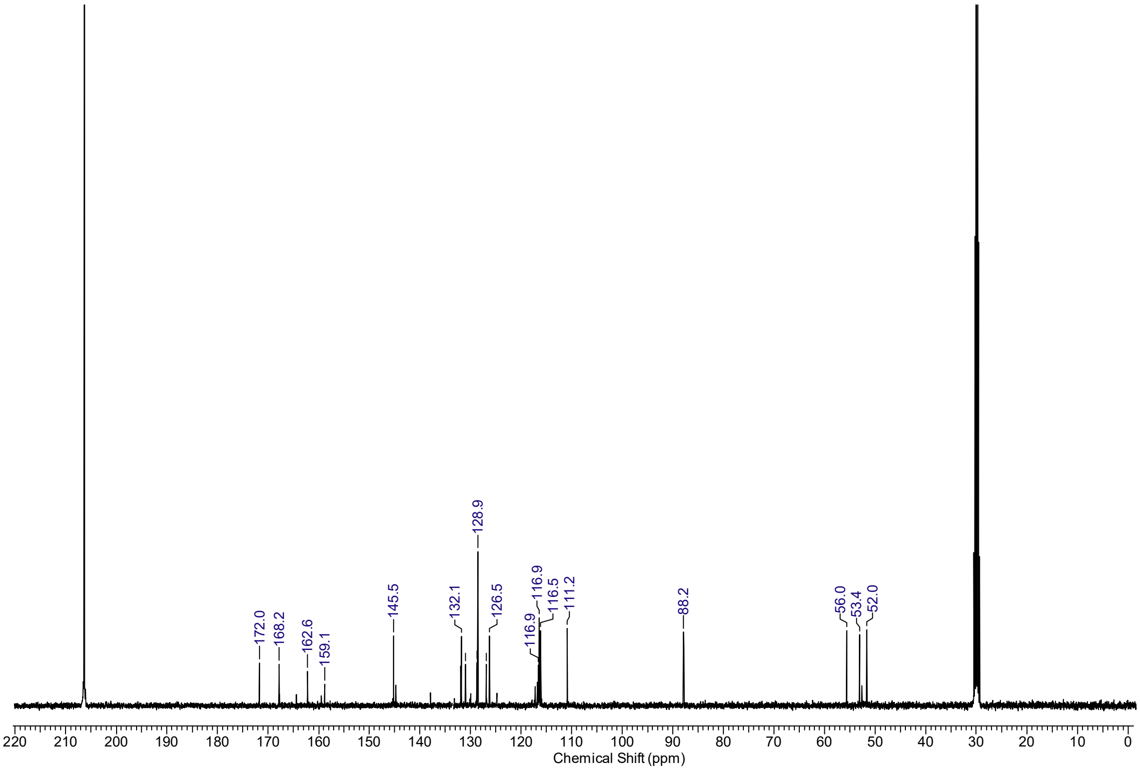

Figure S2. ^{13}C NMR spectra of compound **1** (*Acetone-d_6*, 400 MHz, TMS).

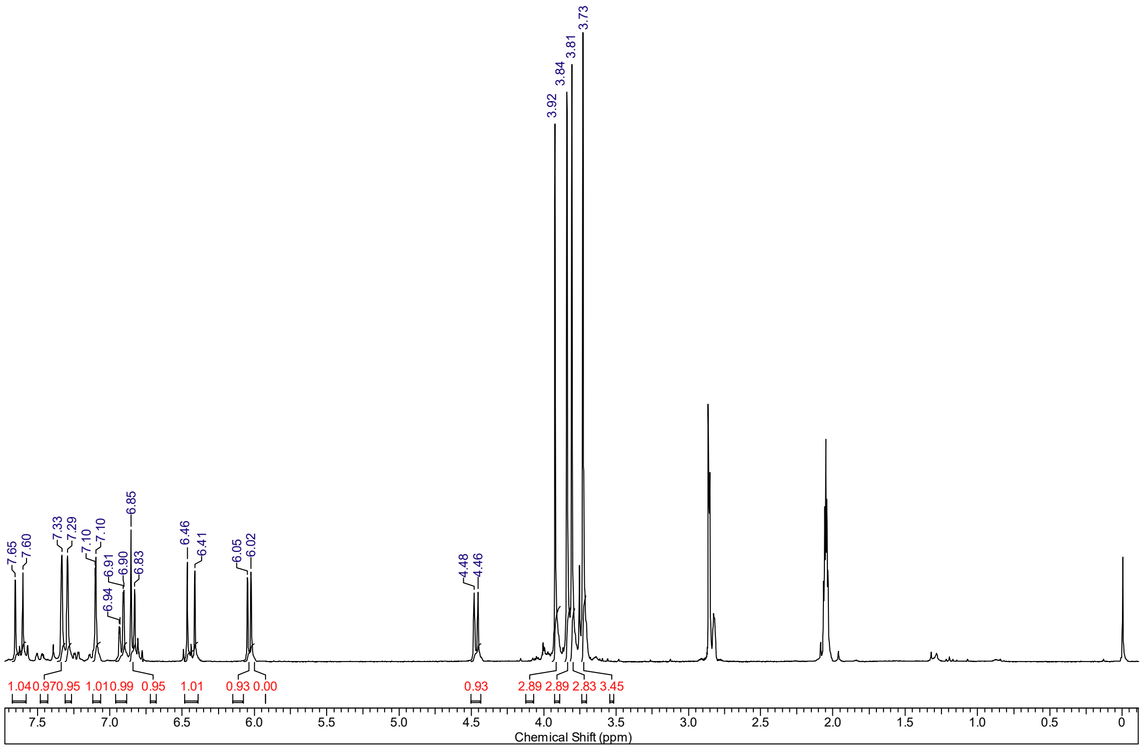

Figure S3. ^{1}H NMR spectra of compound **2** (*Acetone-d$_6$*, 400 MHz, TMS).

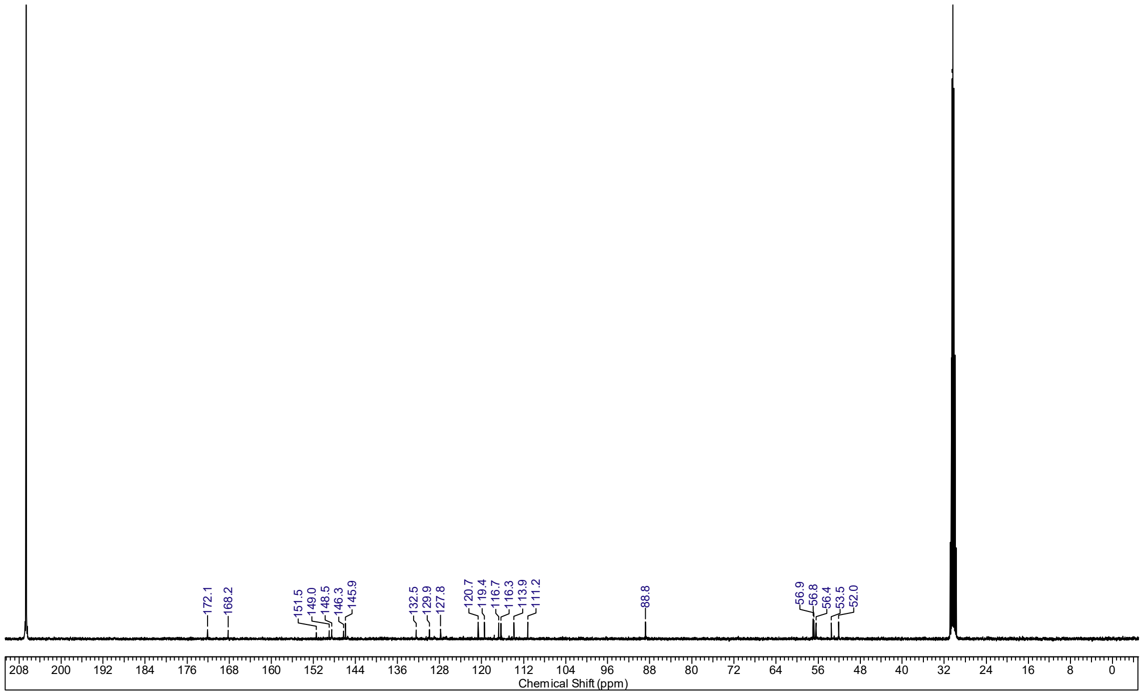

Figure S4. ^{13}C NMR spectra of compound **2** (*Acetone-d$_6$*, 400 MHz, TMS).

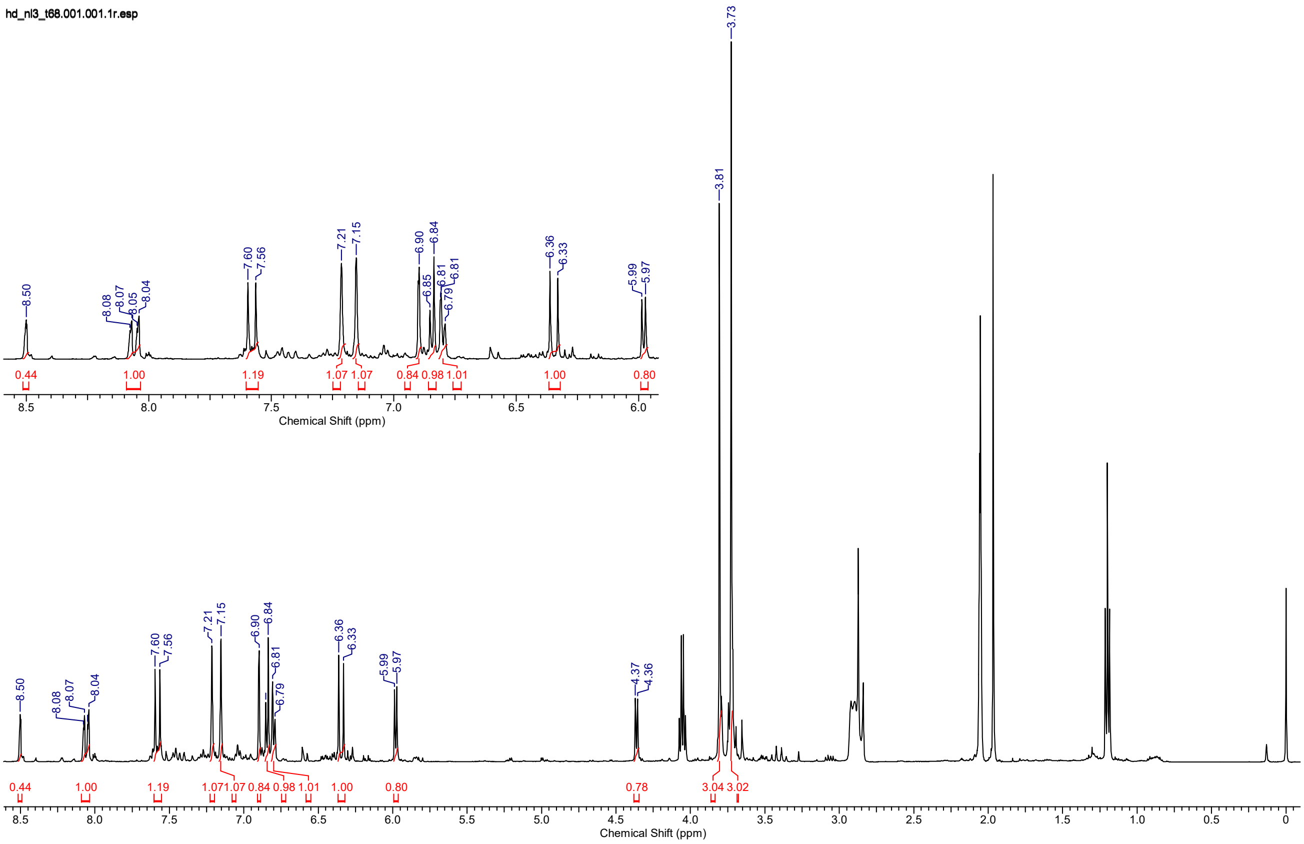

Figure S5. ^{1}H NMR spectra of compound **3** (*Acetone-d$_6$*, 400 MHz, TMS).

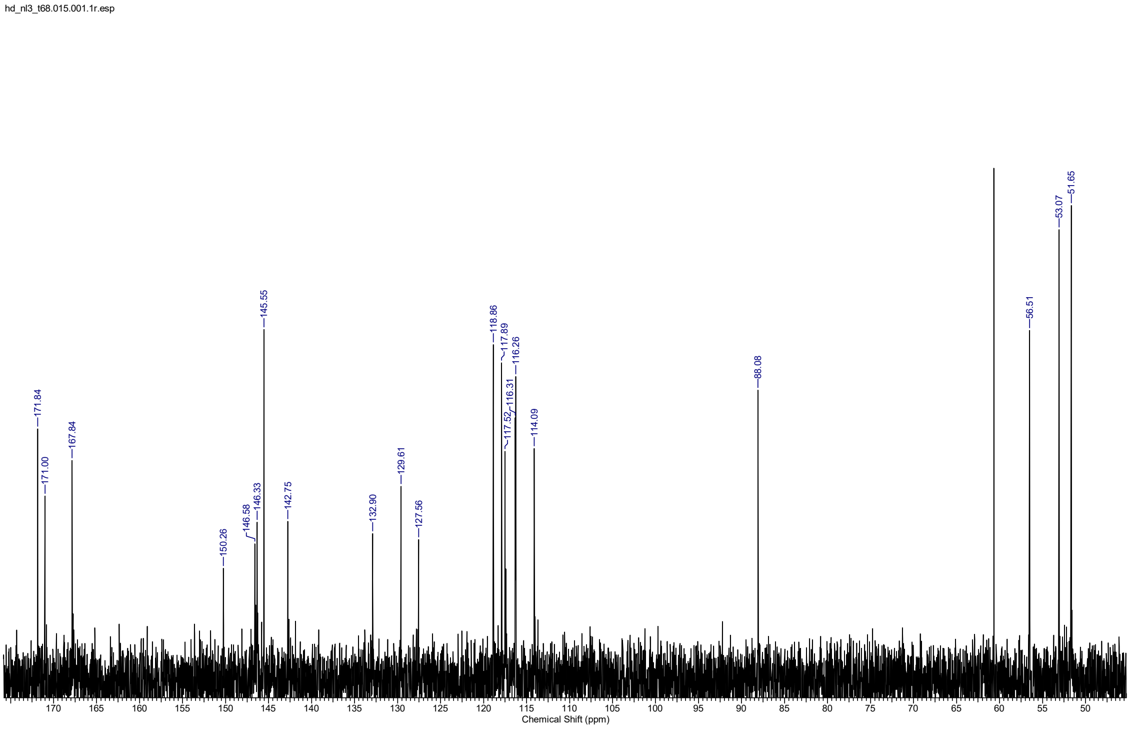

Figure S6. ^{13}C NMR spectra of compound **3** (*Acetone-d$_6$*, 400 MHz, TMS).

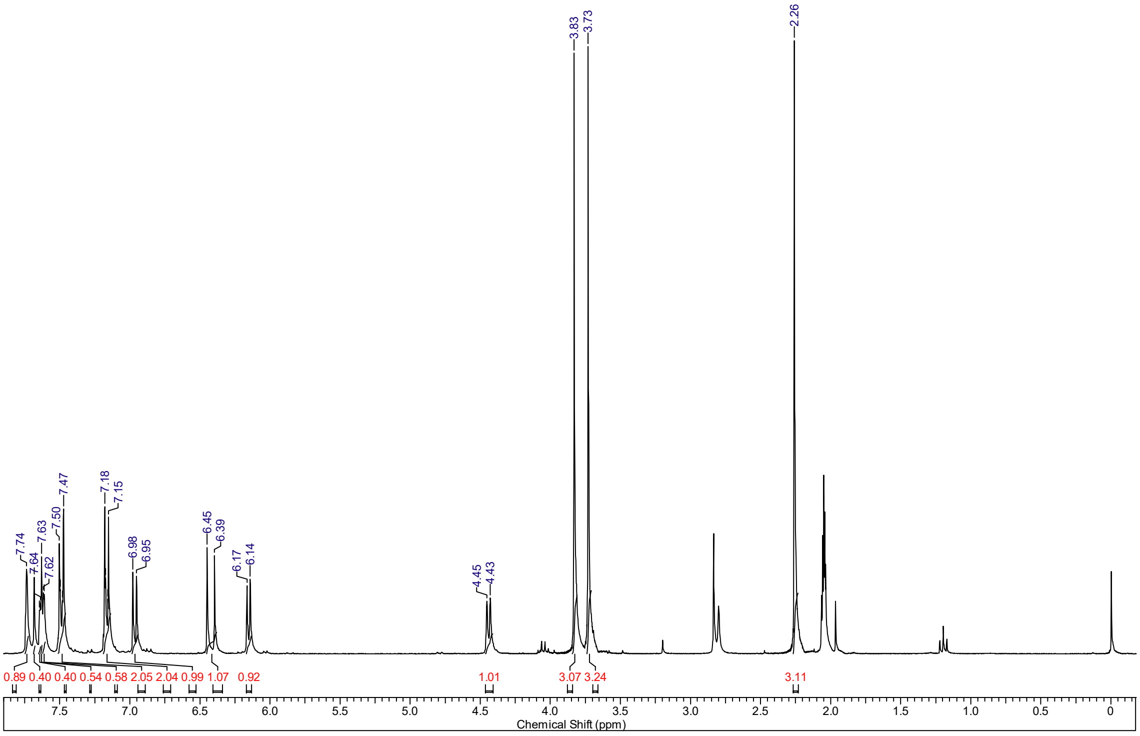

Figure S7. ^{1}H NMR spectra of compound **4** (*Acetone-d_6*, 400 MHz, TMS).

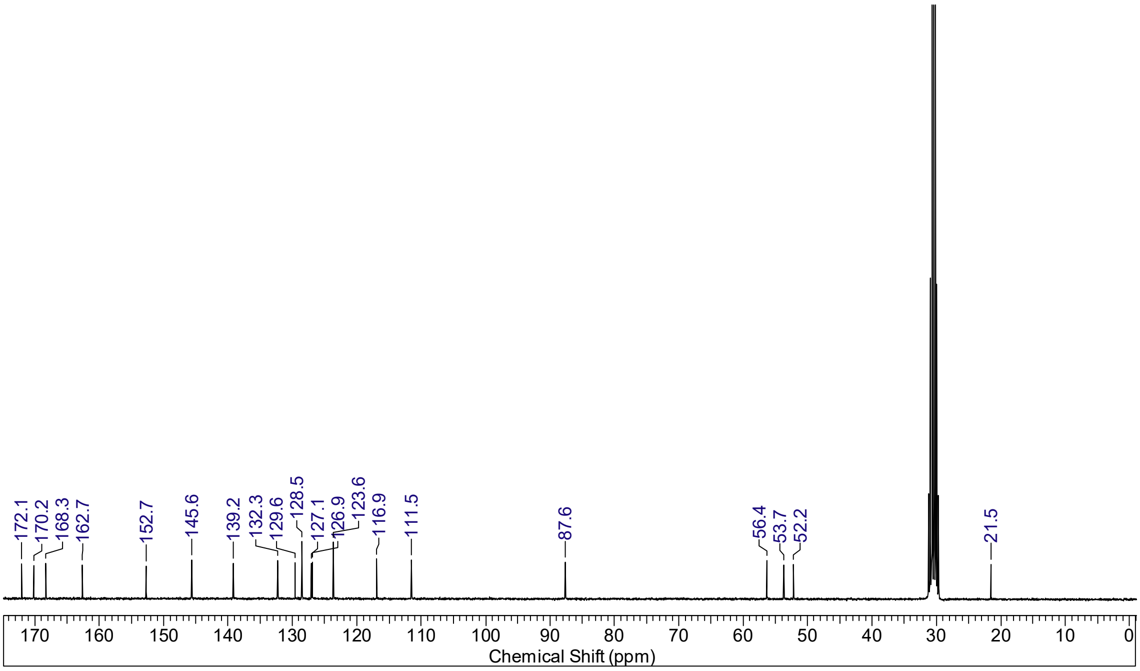

Figure S8. ^{13}C NMR spectra of compound **4** (*Acetone-d_6*, 400 MHz, TMS).

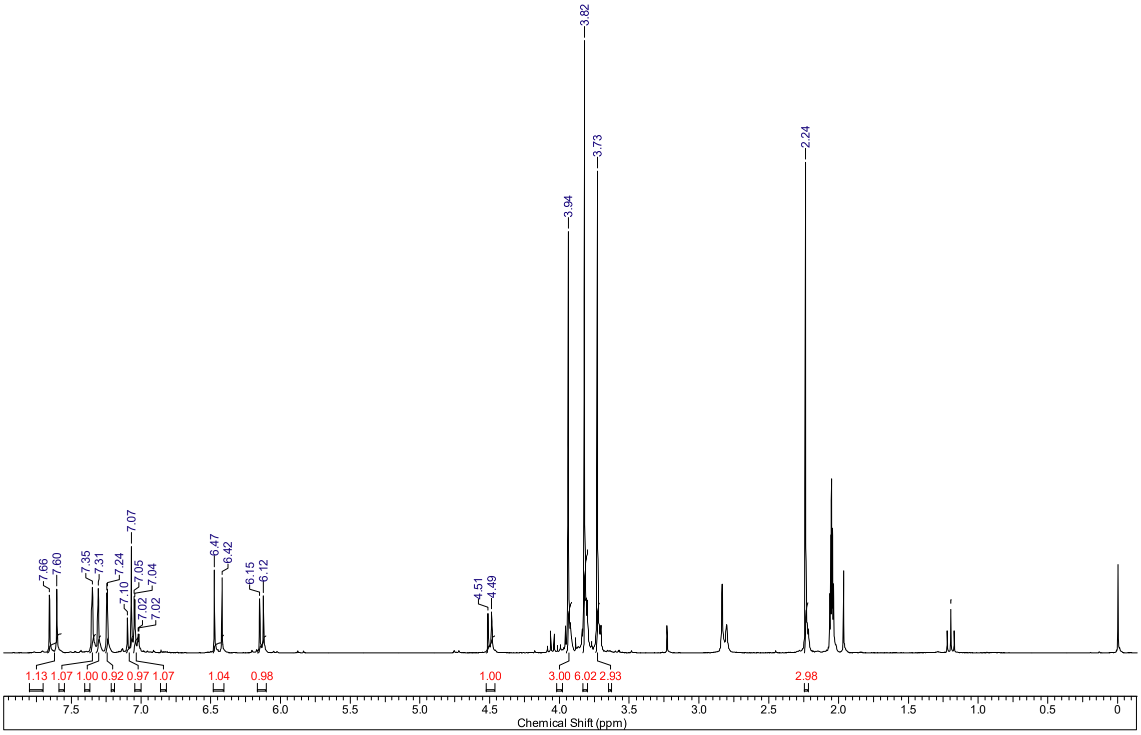

Figure S9. ^{1}H NMR spectra of compound **5** (*Acetone-d$_6$*, 400 MHz, TMS).

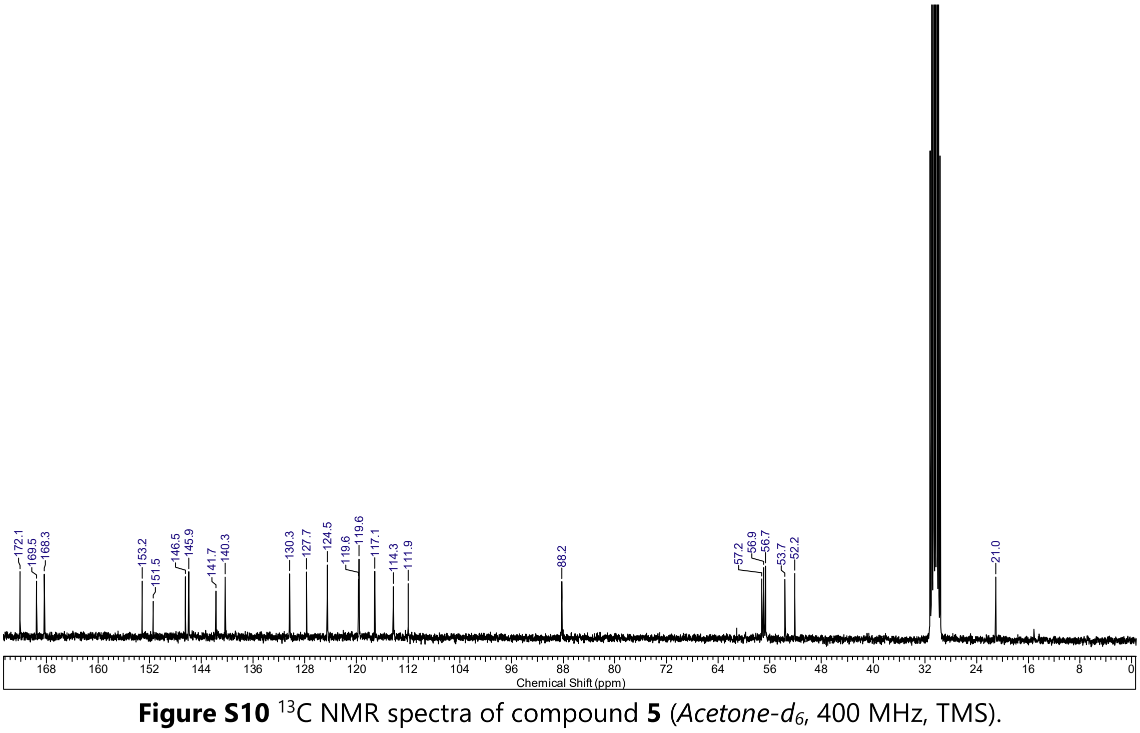

Figure S10 ^{13}C NMR spectra of compound **5** (*Acetone-d$_6$*, 400 MHz, TMS).

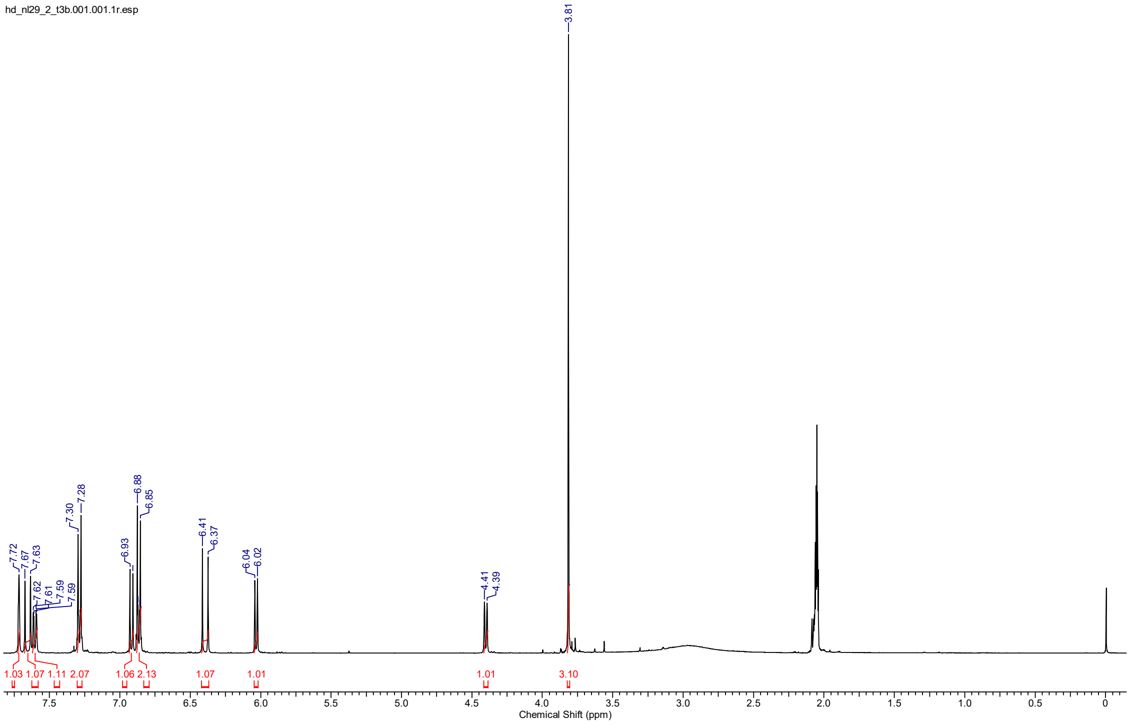

Figure S11. ^{1}H NMR spectra of compound **6** (*Acetone-d$_6$*, 400 MHz, TMS).

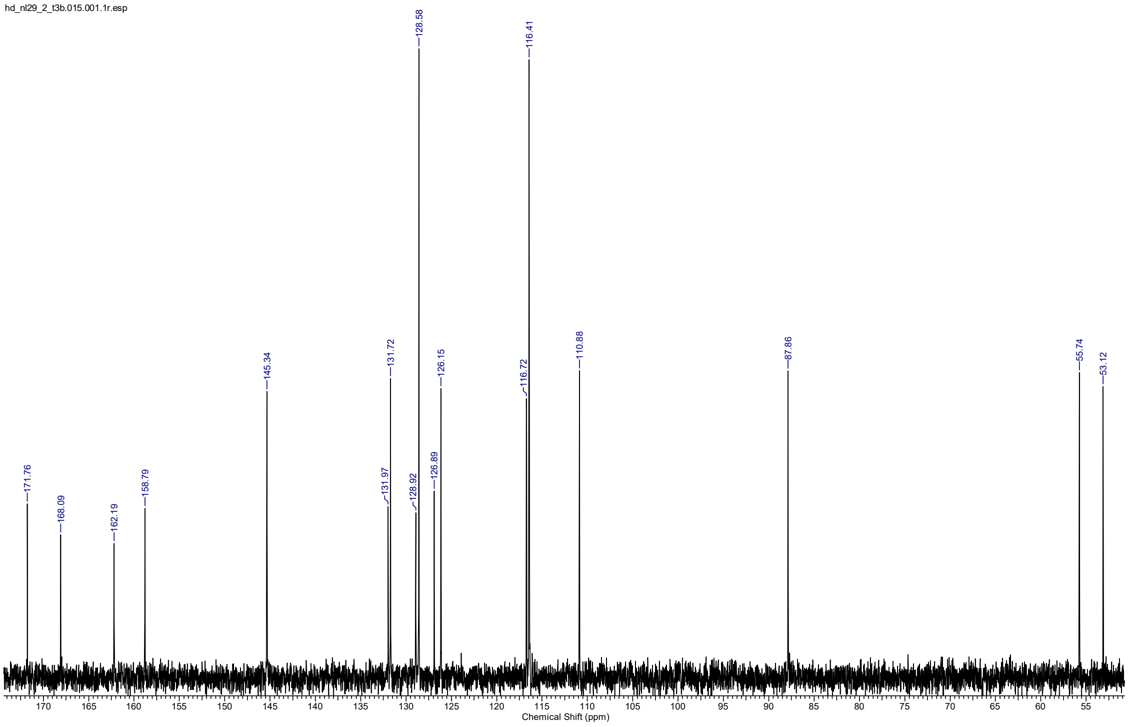

Figure

Figure S12. ^{13}C NMR spectra of compound **6** (*Acetone-d$_6$*, 400 MHz, TMS).

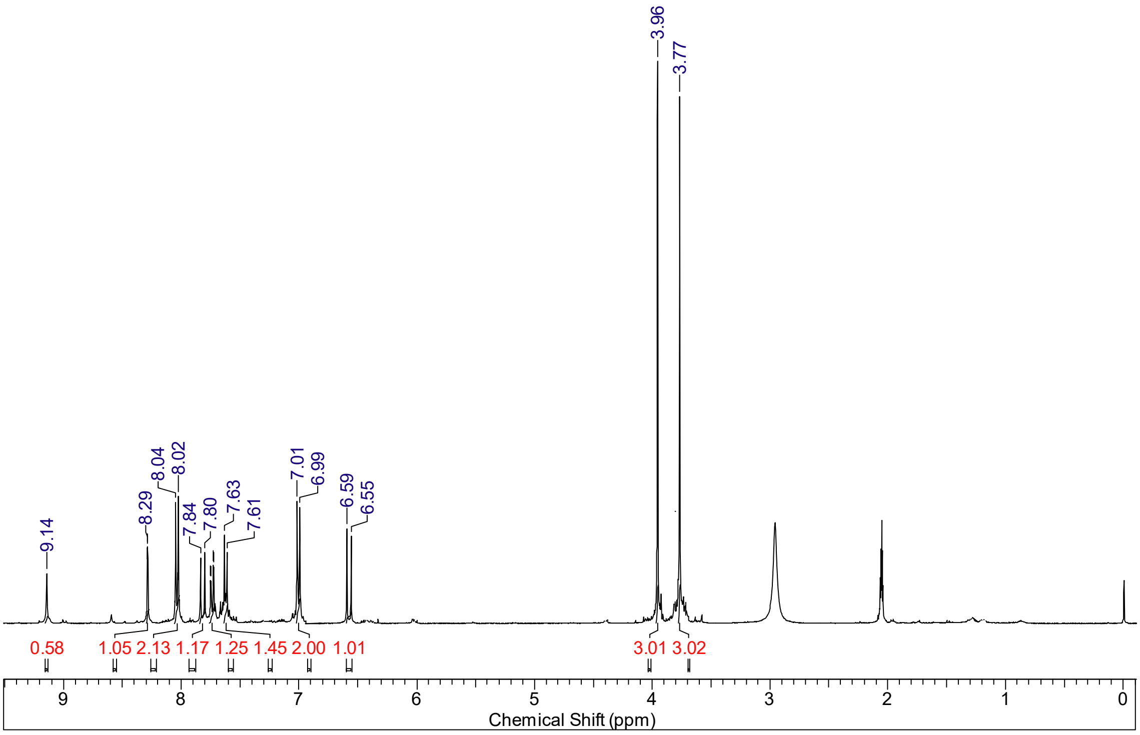

Figure S13. ^{1}H NMR spectra of compound **7** (*Acetone-d_6*, 400 MHz, TMS).

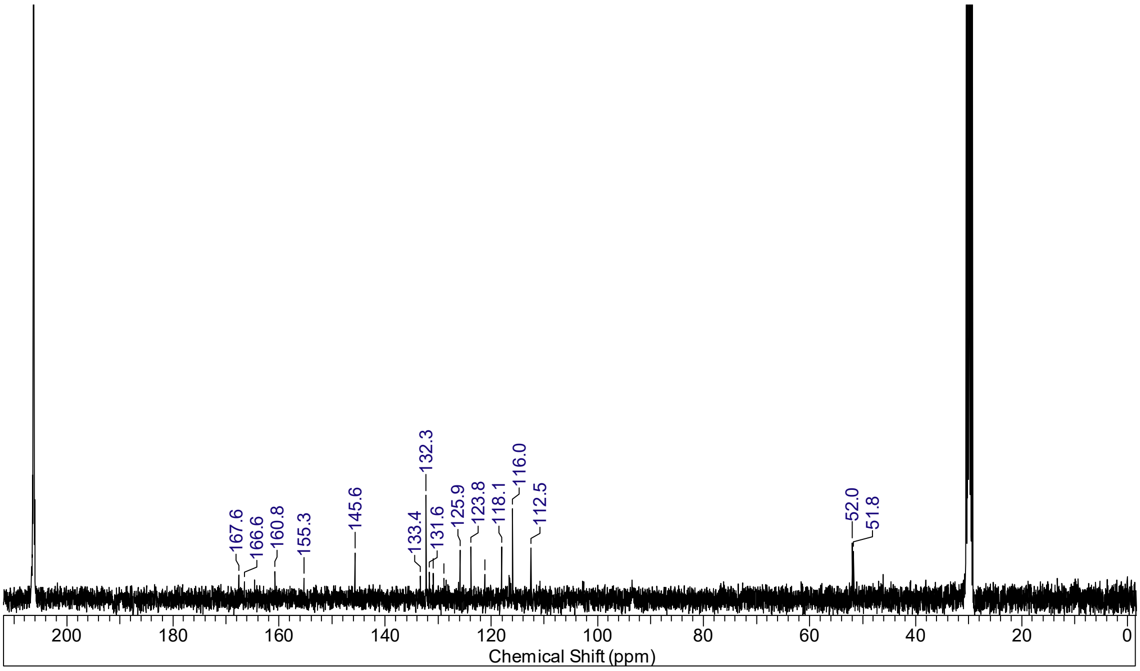

Figure S14. ^{13}C NMR spectra of compound **7** (*Acetone-d_6*, 400 MHz, TMS).

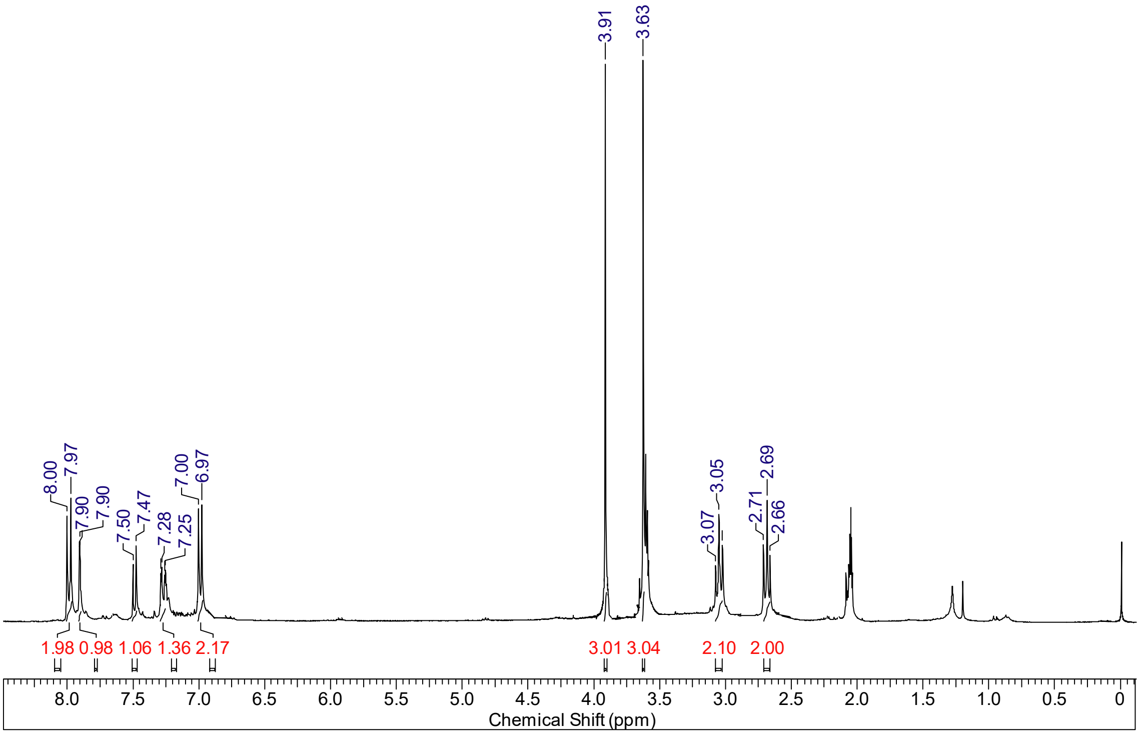

Figure S15 ^{1}H NMR spectra of compound **8** (*Acetone-d_6*, 400 MHz, TMS).

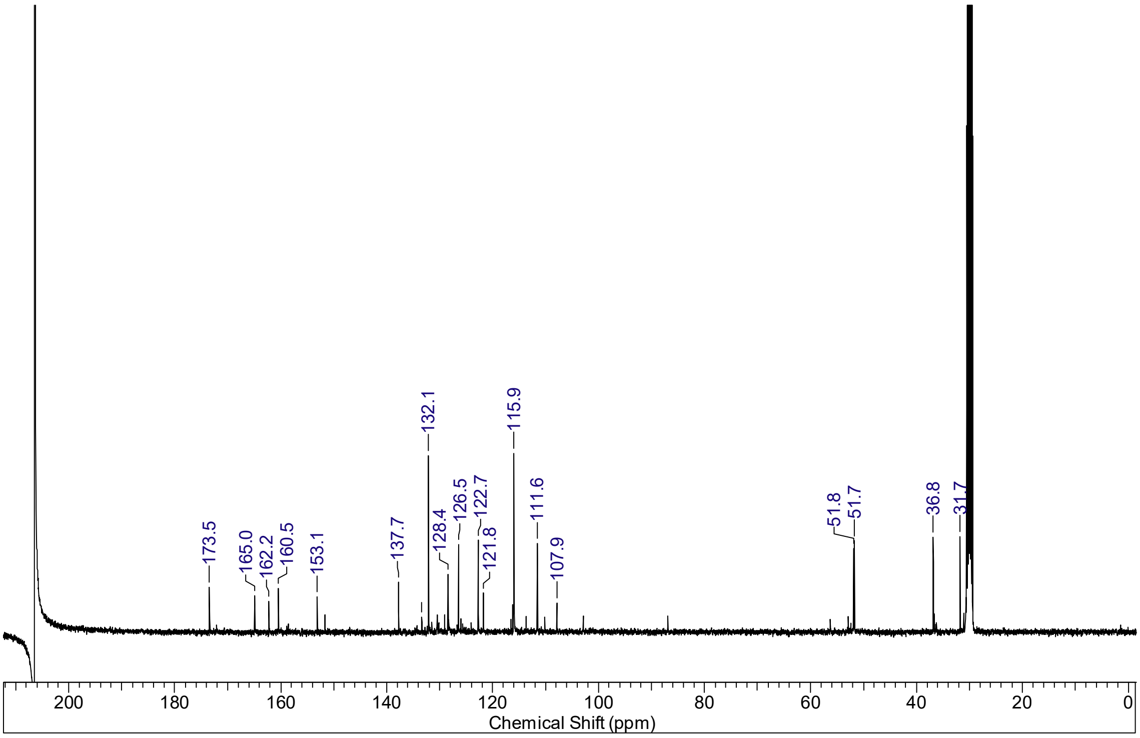

Figure S16. ^{13}C NMR spectra of compound **8** (*Acetone-d_6*, 400 MHz, TMS).

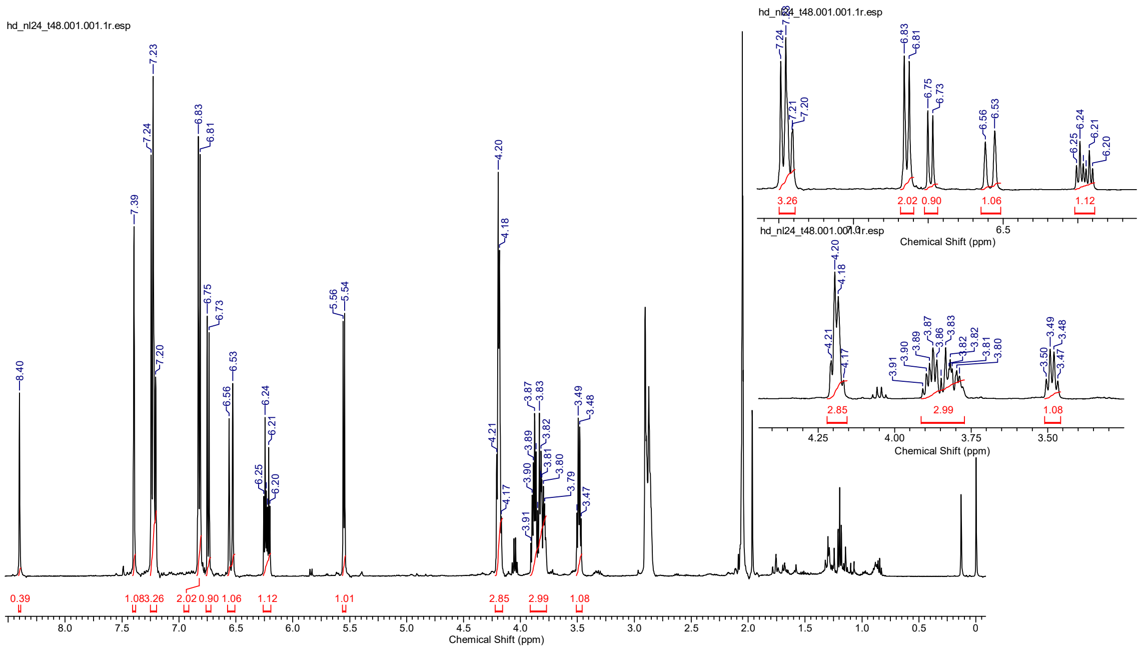

Figure S17. ^{1}H NMR spectra of compound **9** (*Acetone-d$_6$*, 400 MHz, TMS).

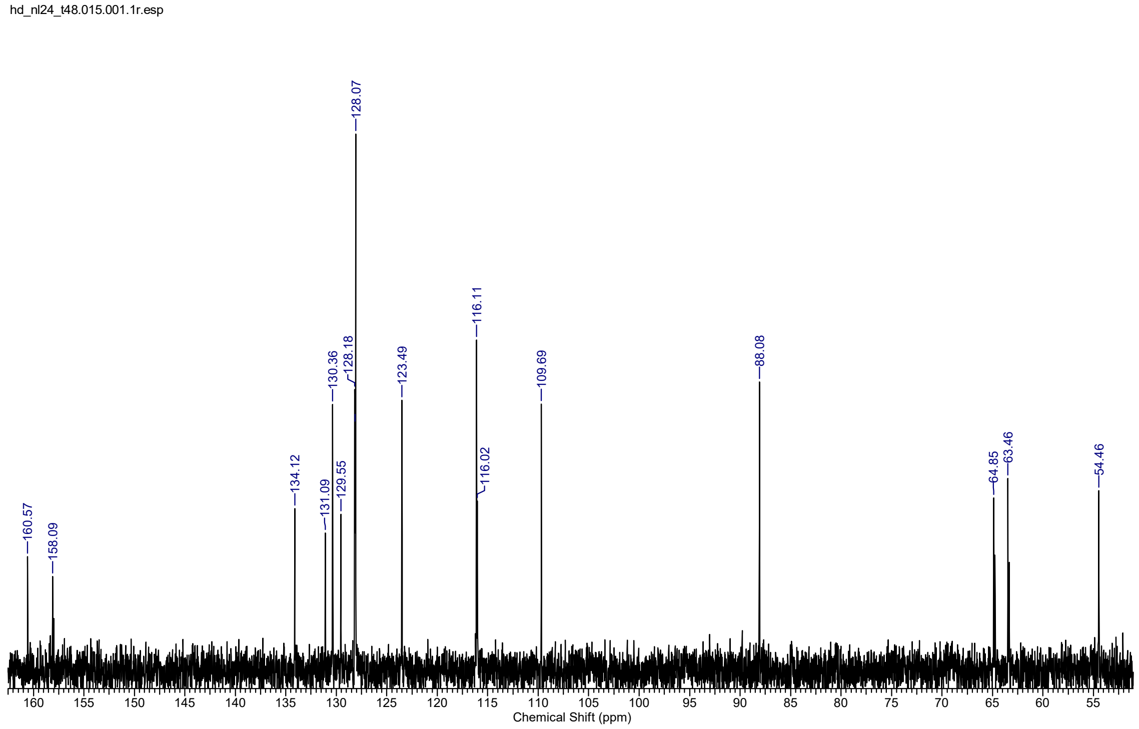

Figure S18. ^{13}C NMR spectra of compound **9** (*Acetone-d$_6$*, 400 MHz, TMS).

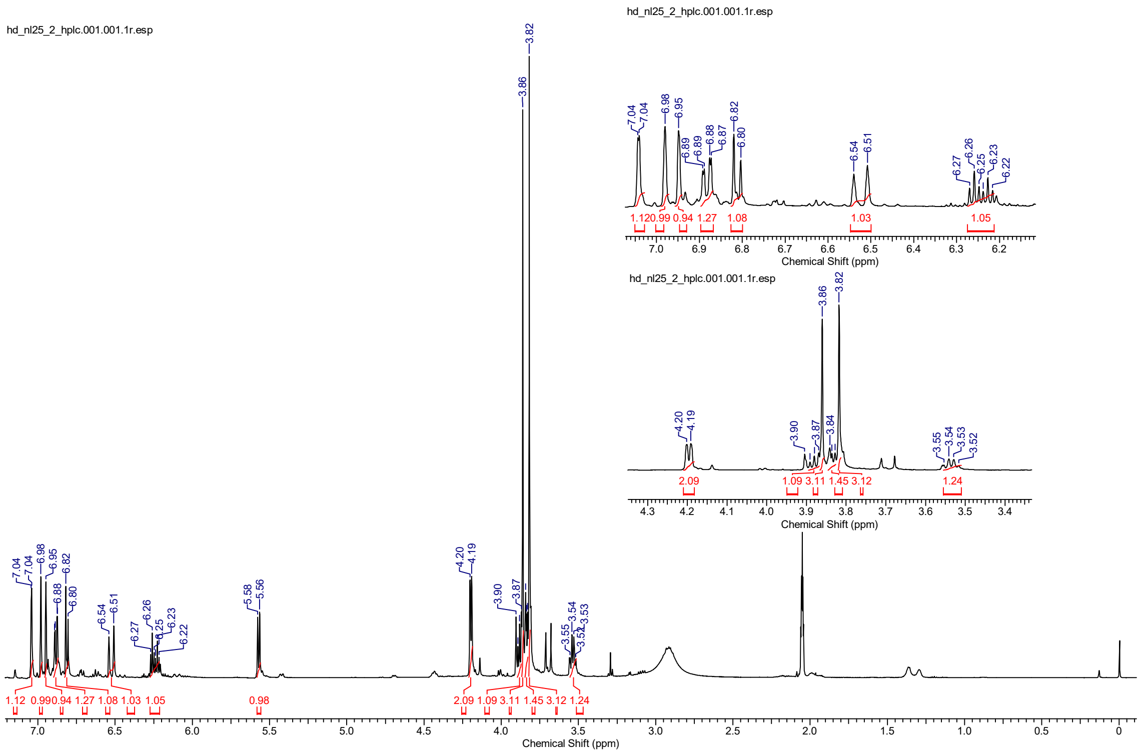

Figure S19. ^{1}H NMR spectra of compound **10** (*Acetone-d$_6$*, 400 MHz, TMS).

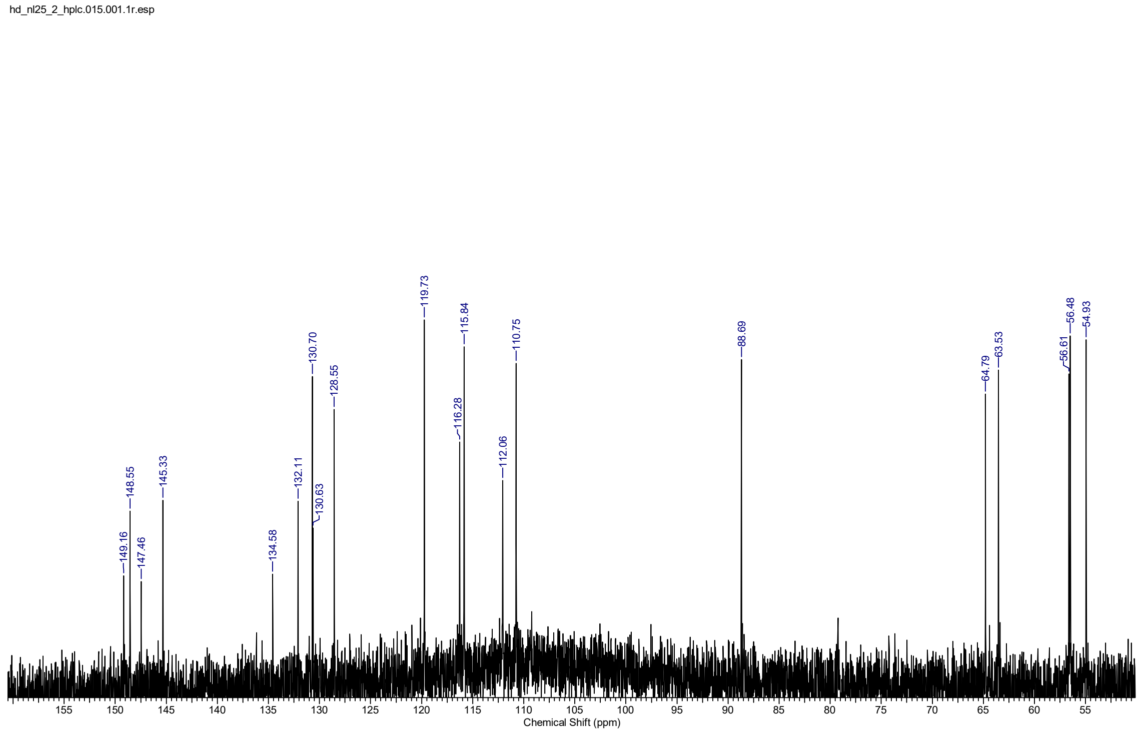

Figure S20. ^{13}C NMR spectra of compound **10** (*Acetone-d$_6$*, 400 MHz, TMS).

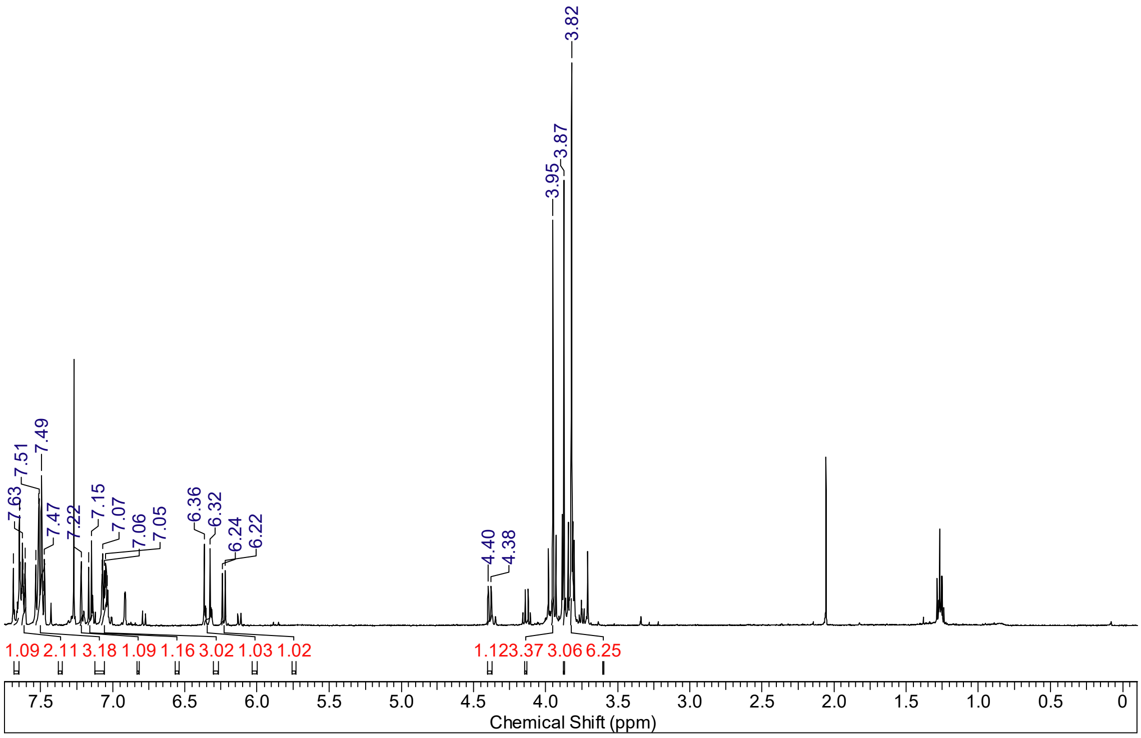

Figure S21. ^{1}H NMR spectra of compound **11** ($CDCl_3$, 400 MHz, TMS).

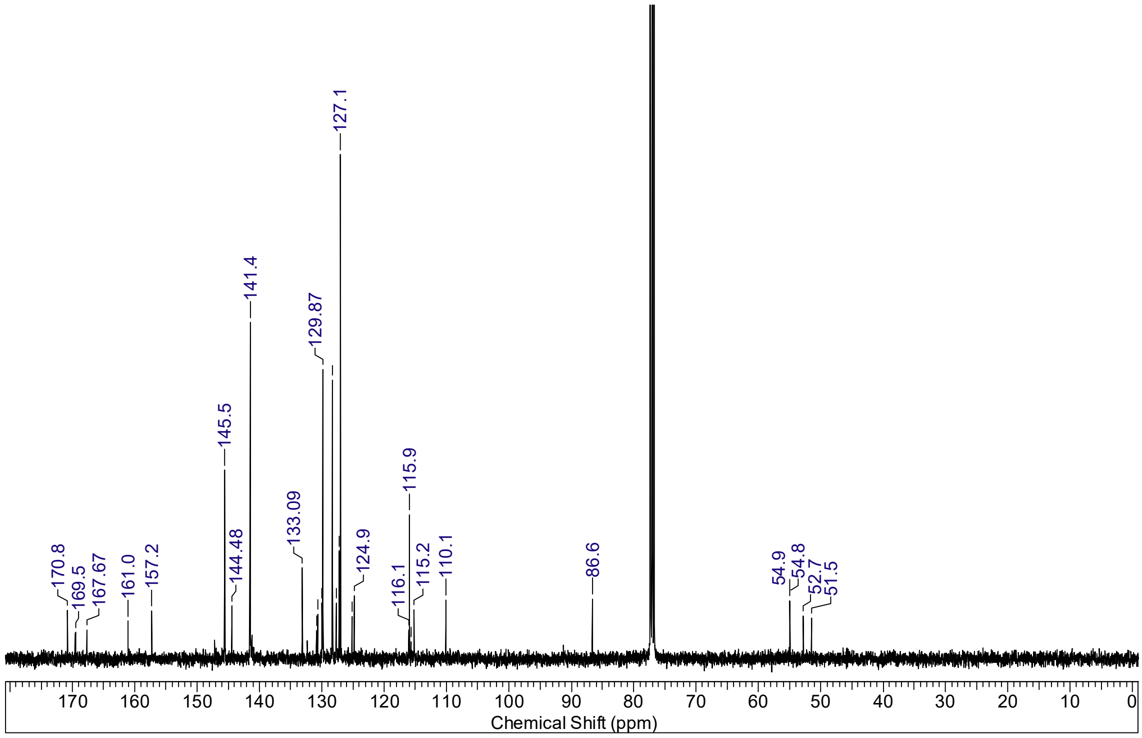

Figure S22. ^{13}C NMR spectra of compound **11** ($CDCl_3$, 400 MHz, TMS).

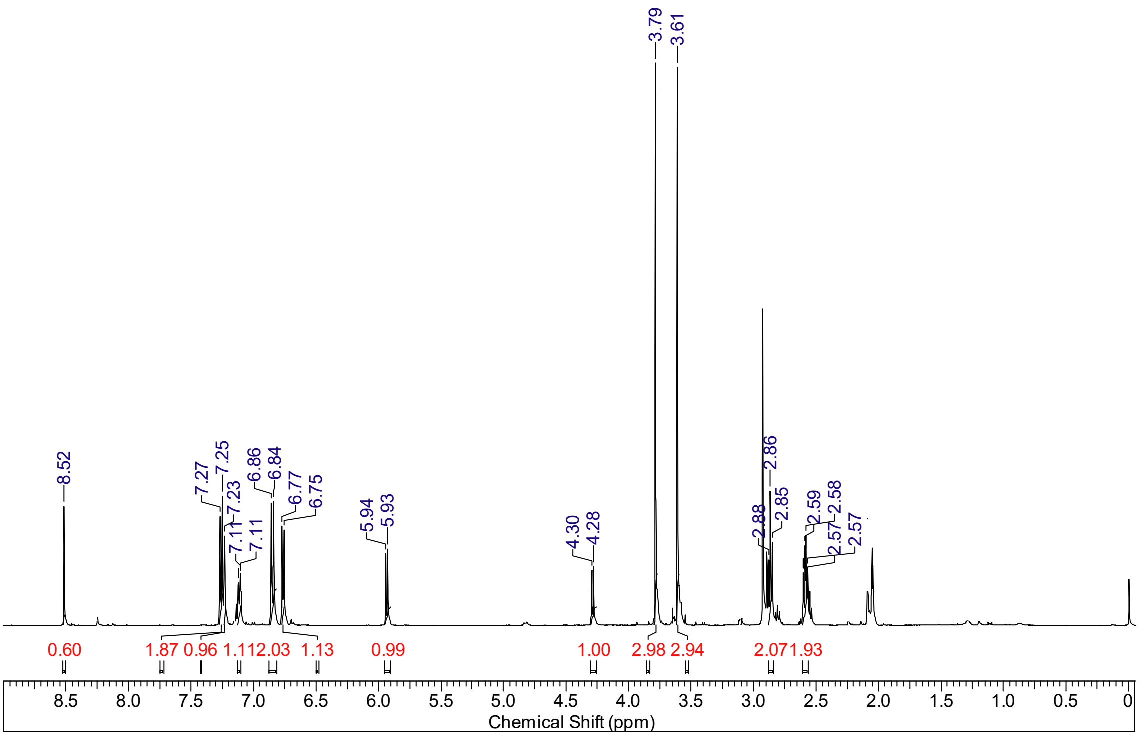

Figure S23. ^{1}H NMR spectra of compound **12** (*Acetone-d_6*, 400 MHz, TMS).

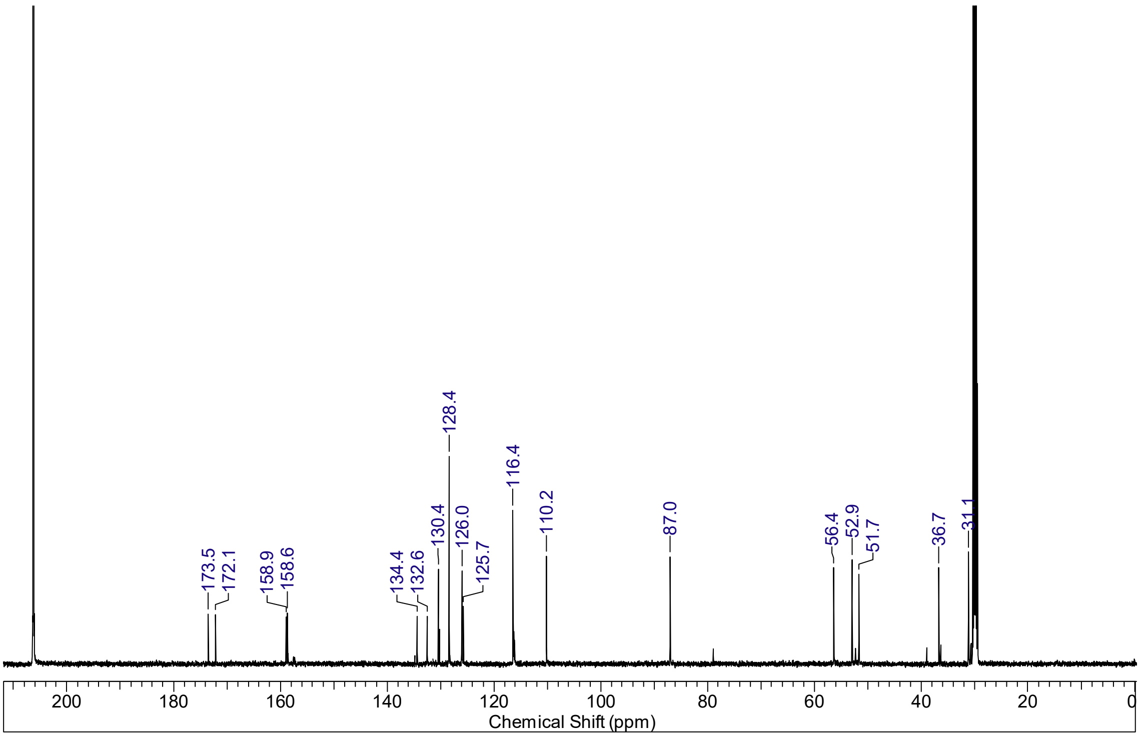

Figure S24. ^{13}C NMR spectra of compound **12** (*Acetone-d_6*, 400 MHz, TMS).

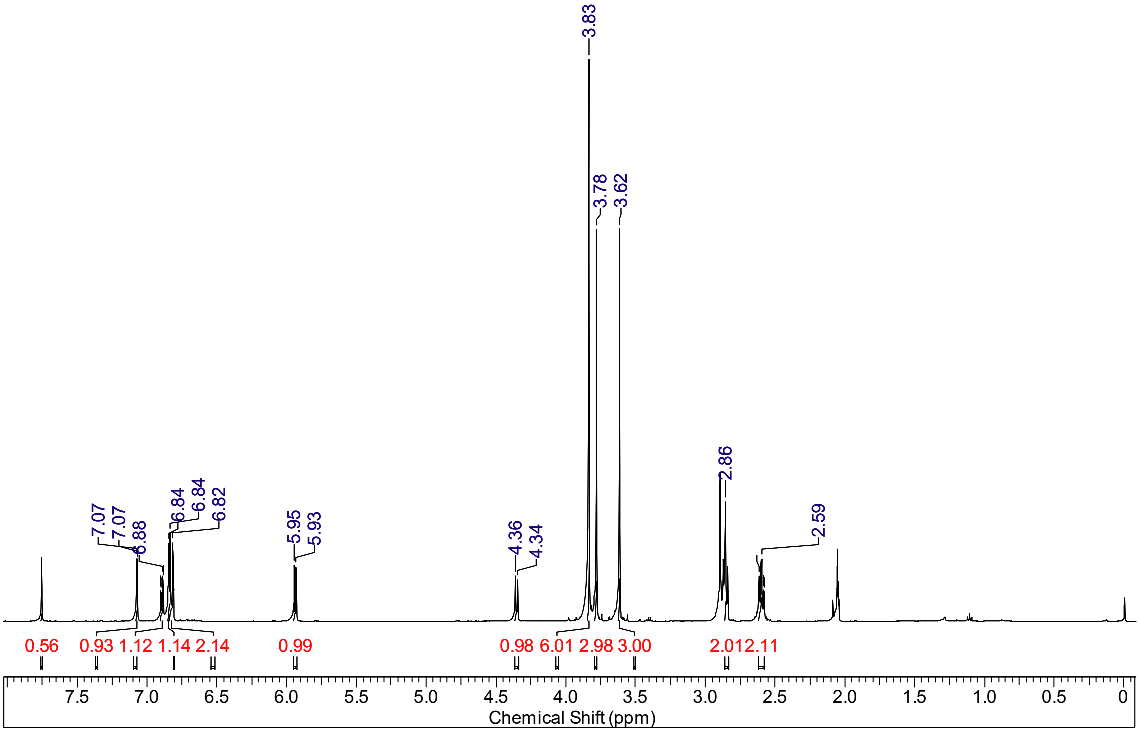

Figure S25. ^{1}H NMR spectra of compound **13** (*Acetone-d$_6$*, 400 MHz, TMS).

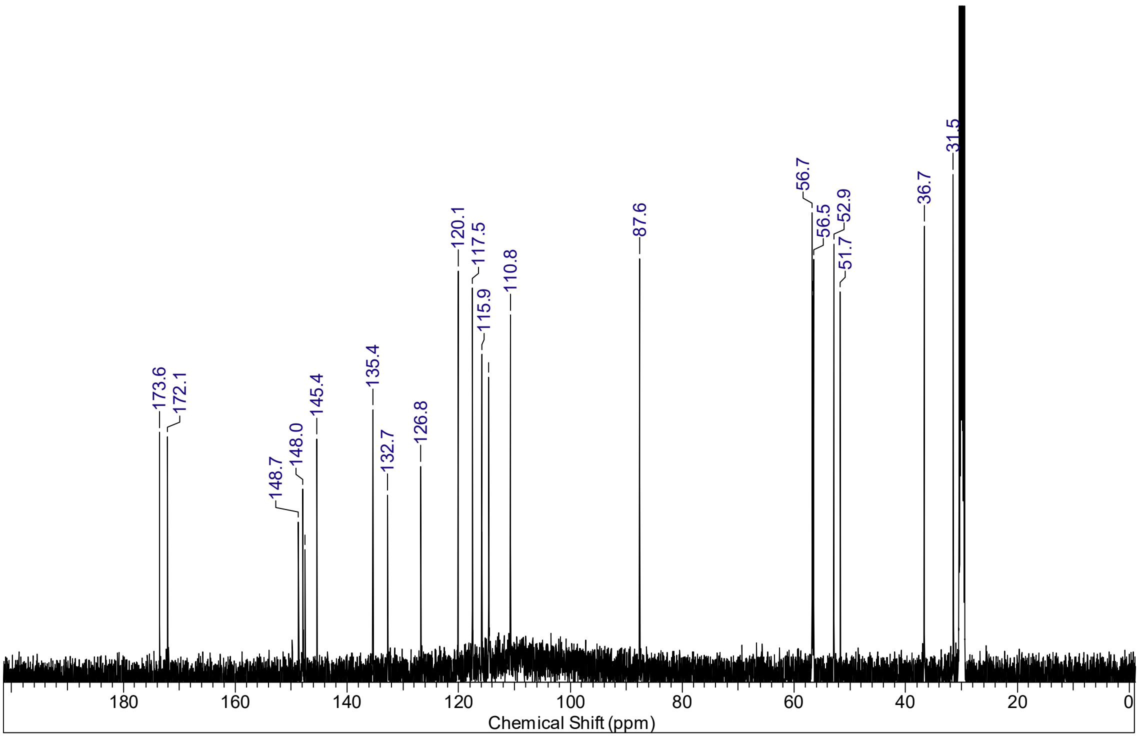

Figure S26. ^{13}C NMR spectra of compound **13** (*Acetone-d$_6$*, 400 MHz, TMS).

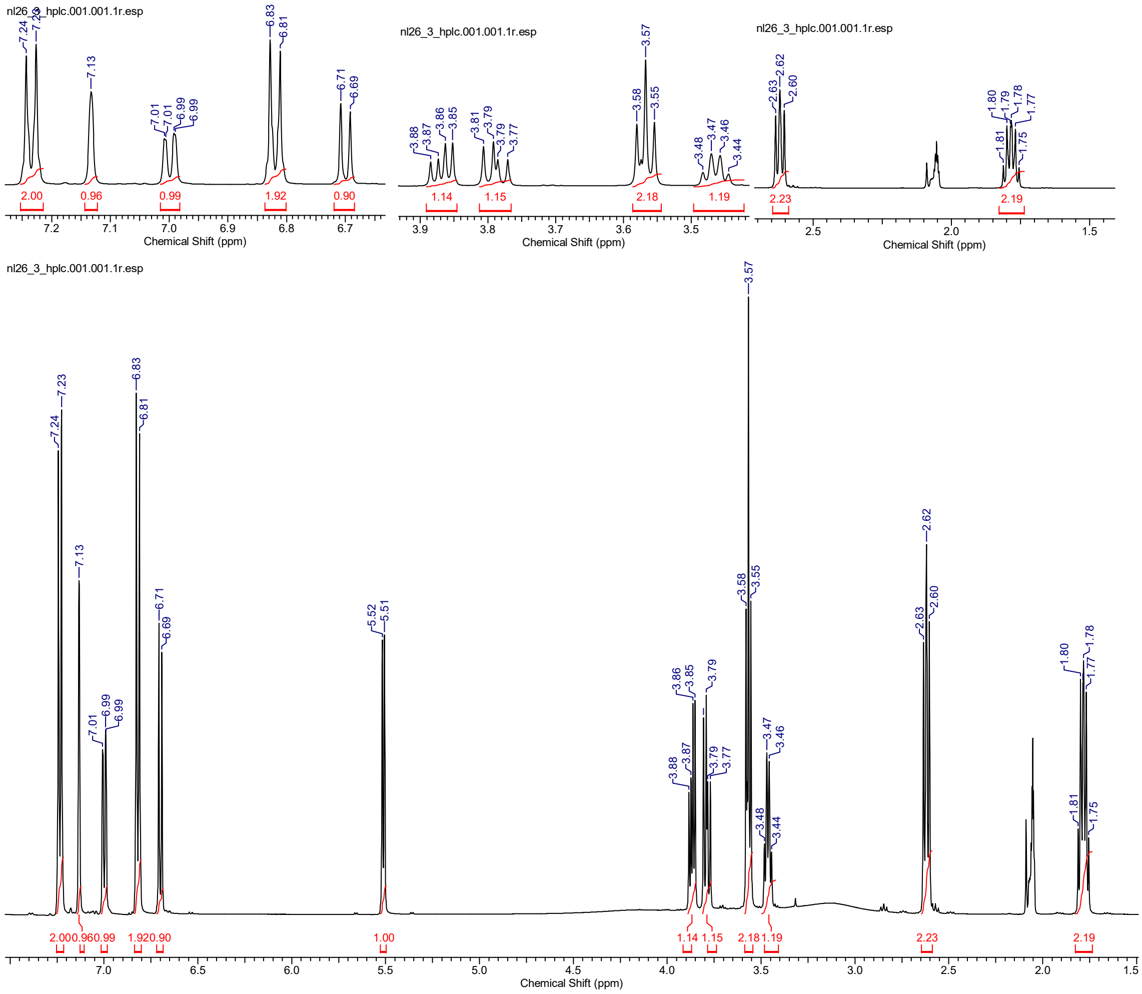

Figure S27. ^{1}H NMR spectra of compound **14** (*Acetone-d*$_6$, 400 MHz, TMS).

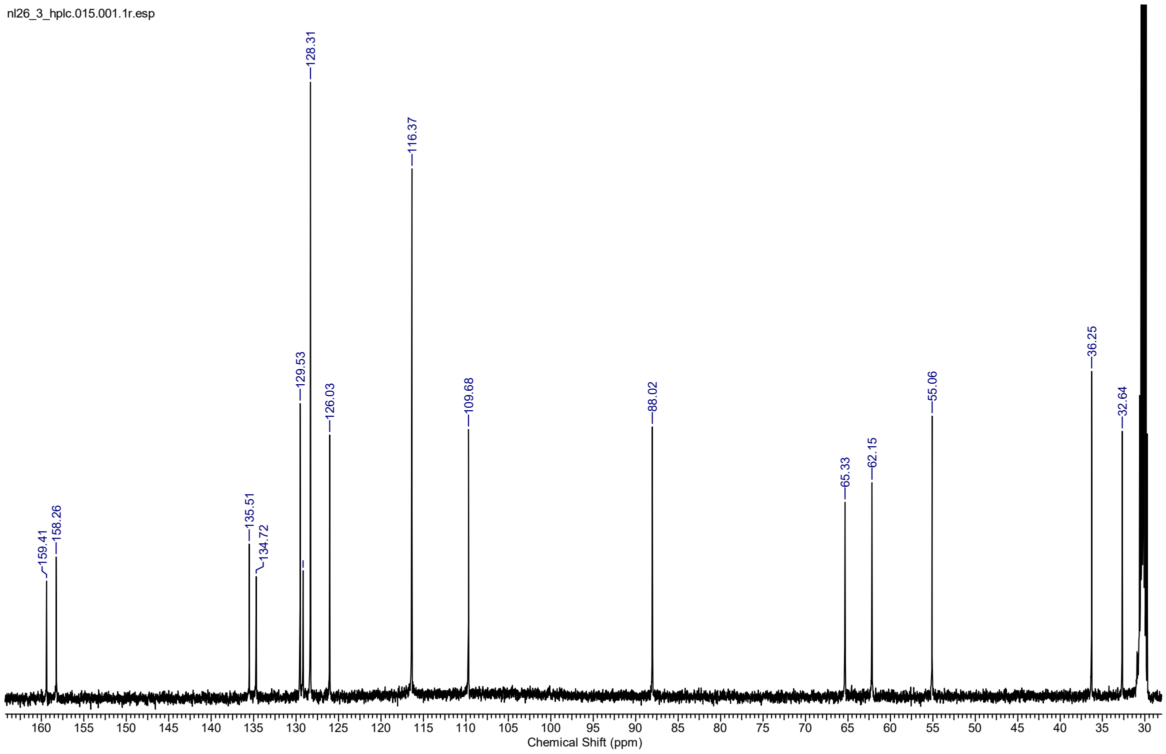

Figure S28. ^{13}C NMR spectra of compound **14** (*Acetone-d*$_6$, 400 MHz, TMS).

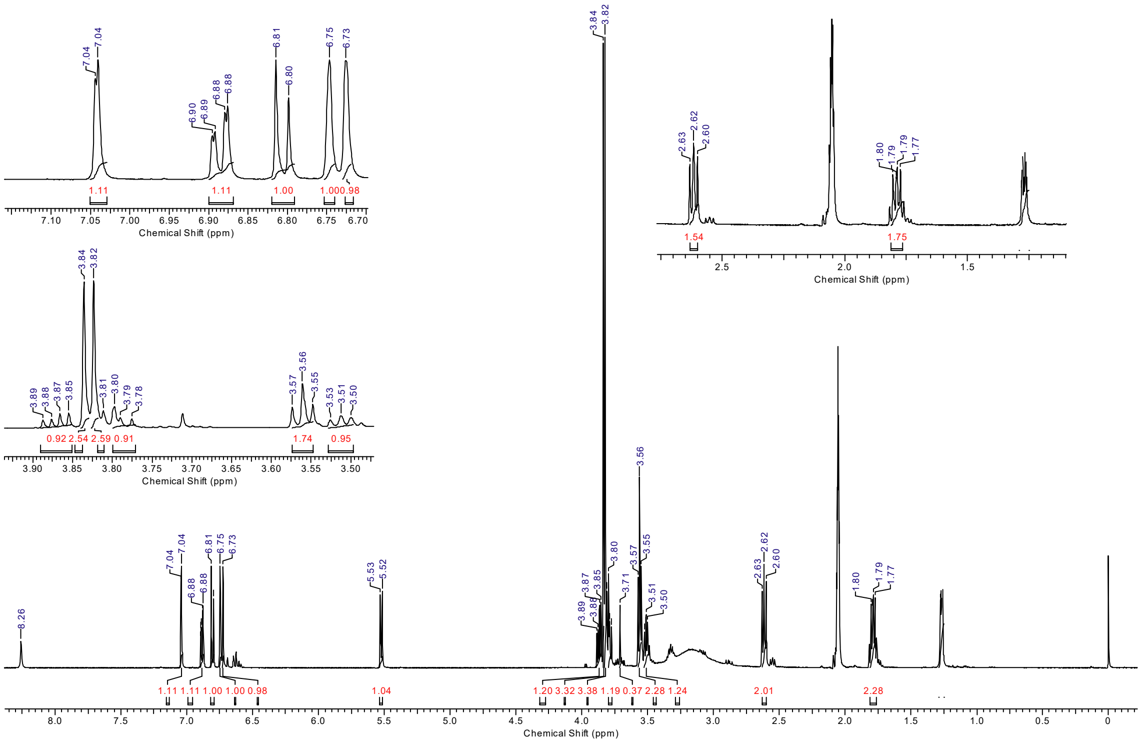

Figure S29. ^{1}H NMR spectra of compound **15** (*Acetone-d_6*, 400 MHz, TMS).

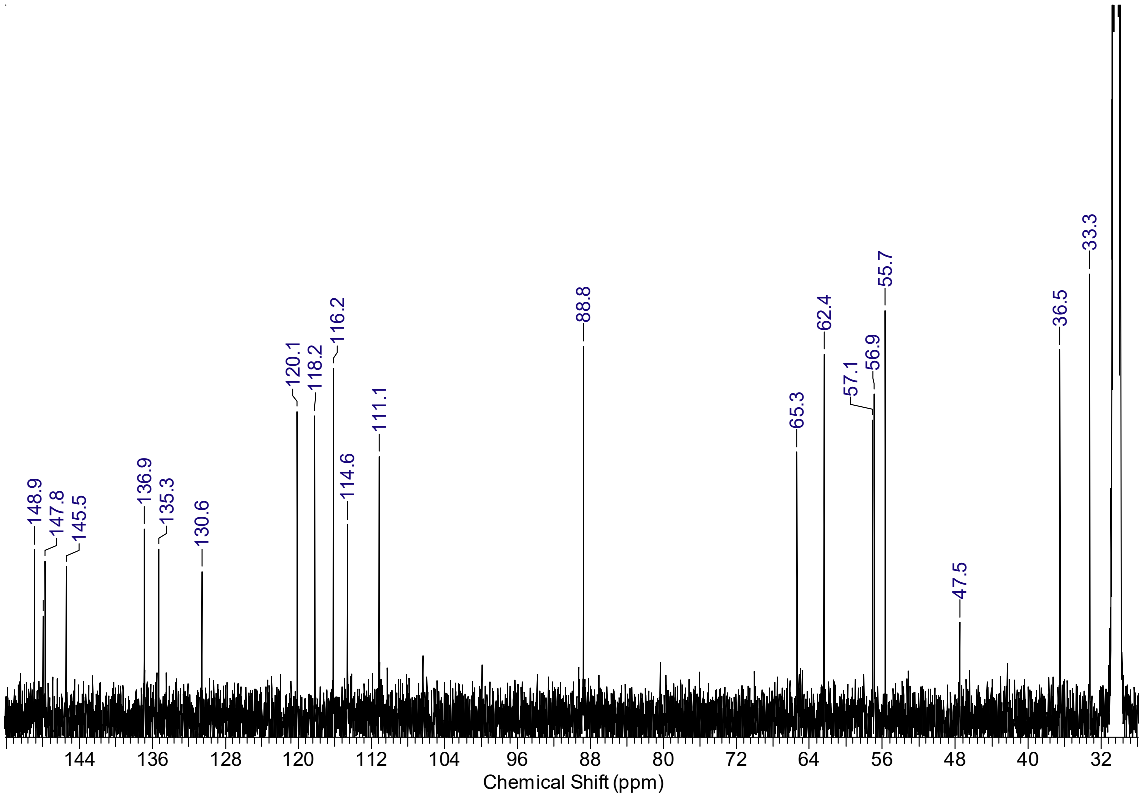

Figure S30. ^{13}C NMR spectra of compound **15** (*Acetone-d_6*, 400 MHz, TMS).

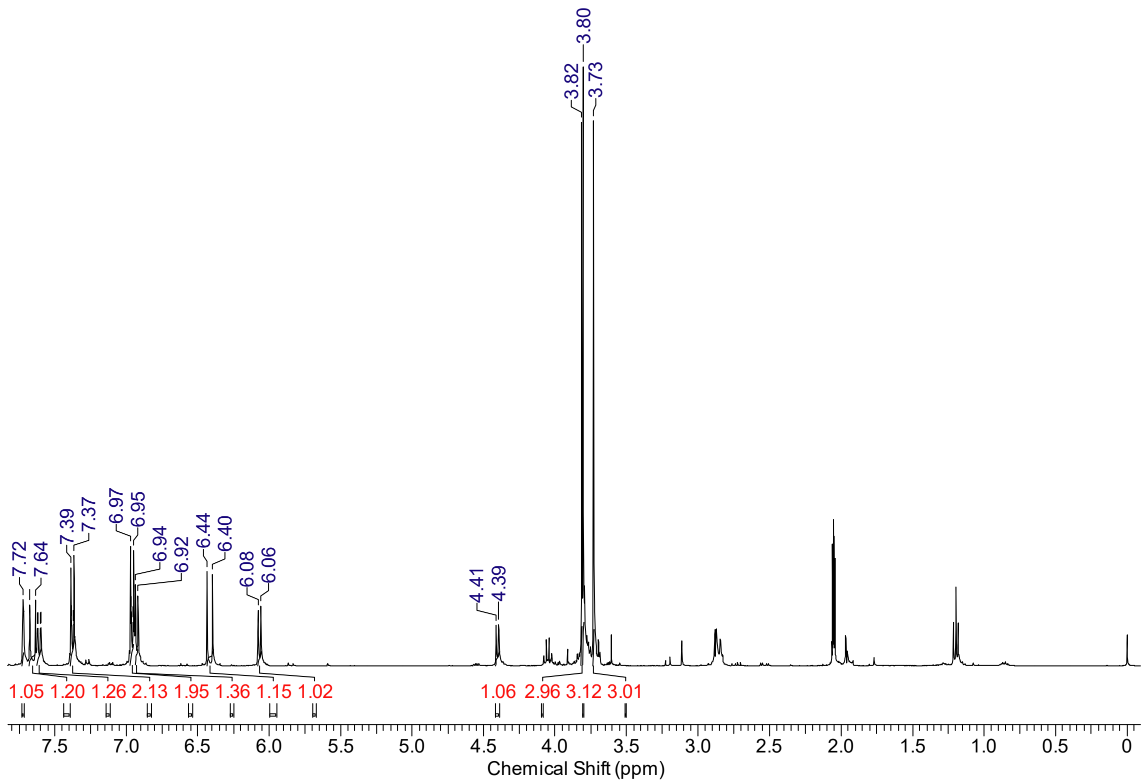

Figure S31. ^{1}H NMR spectra of compound **16** (*Acetone-d_6*, 400 MHz, TMS).

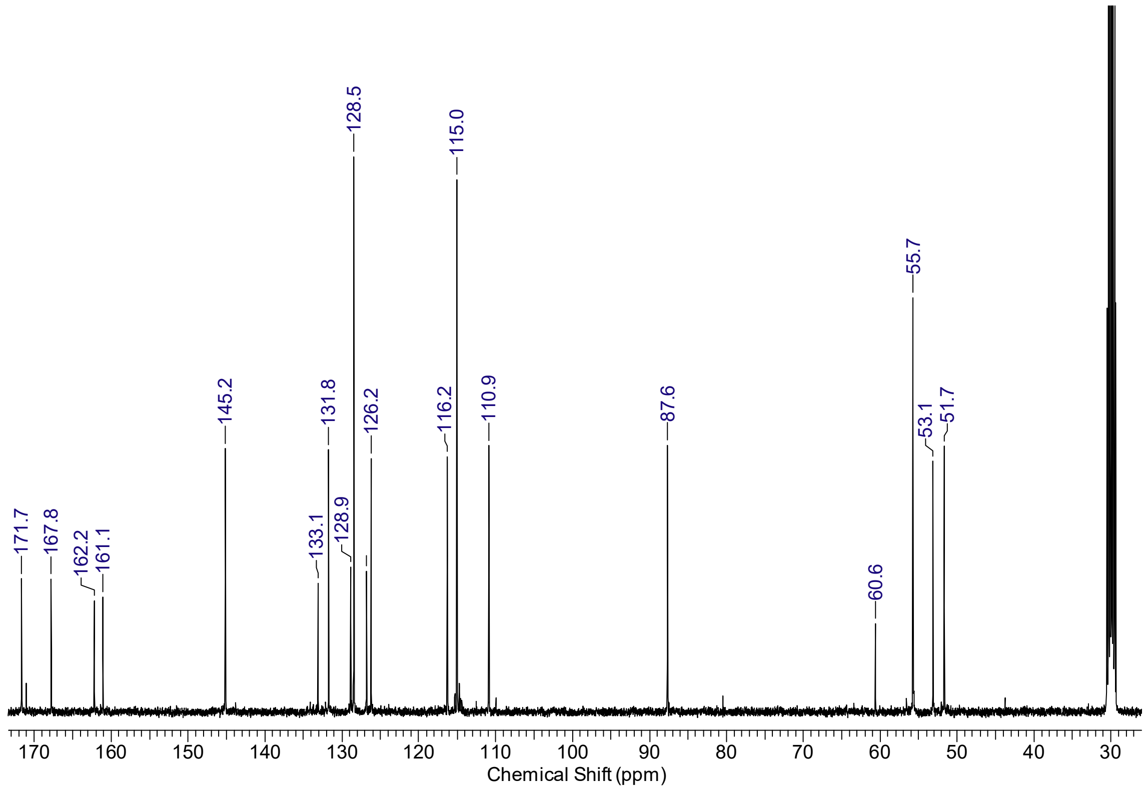

Figure S32. ^{13}C NMR spectra of compound **16** (*Acetone-d_6*, 400 MHz, TMS).

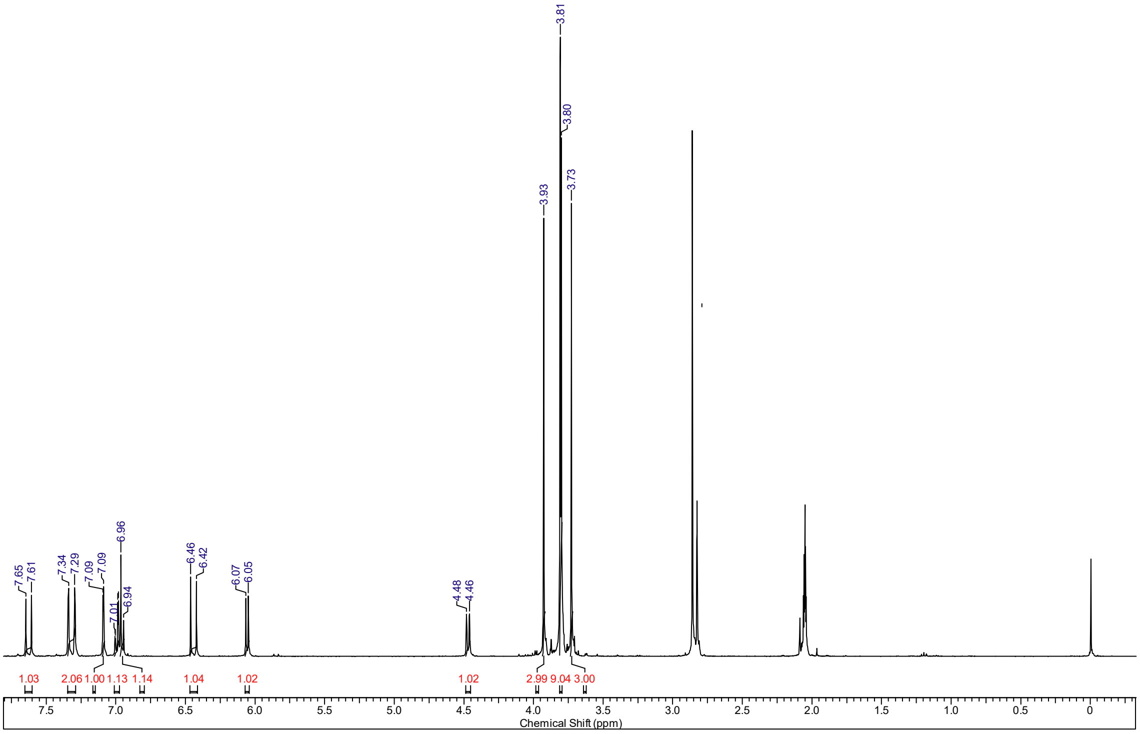

Figure S33. ^{1}H NMR spectra of compound **17** (*Acetone-d$_6$*, 400 MHz, TMS).

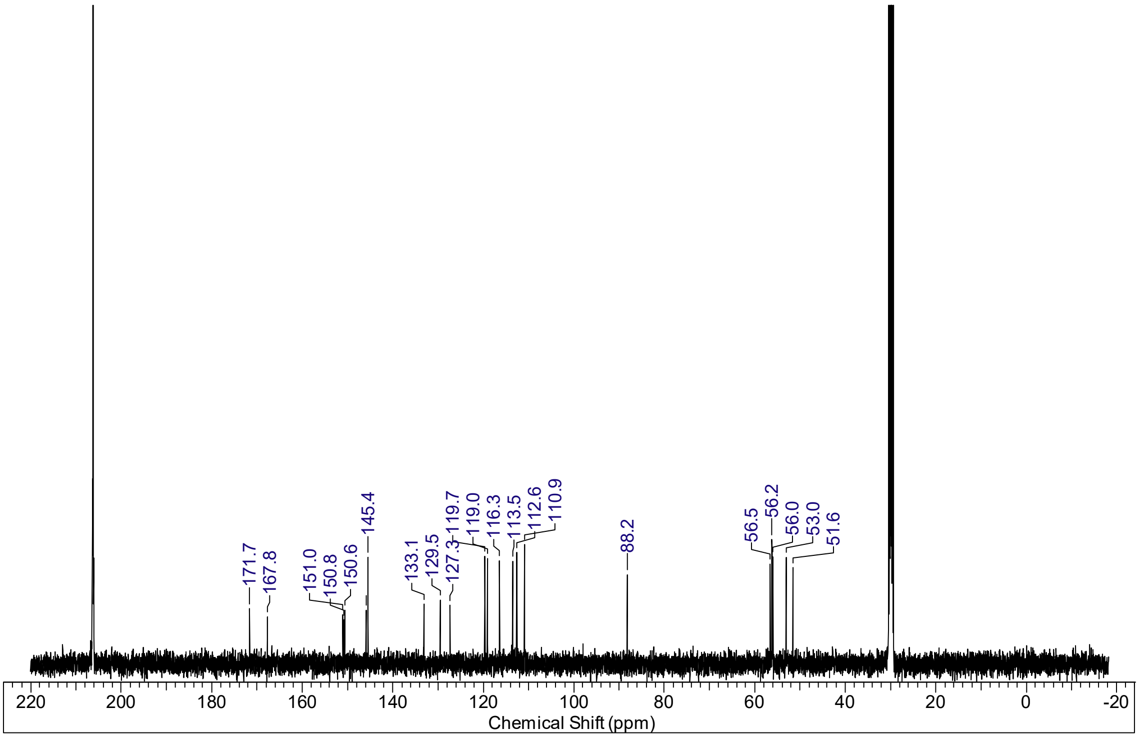

Figure S34. ^{13}C NMR spectra of compound **17** (*Acetone-d$_6$*, 400 MHz, TMS).

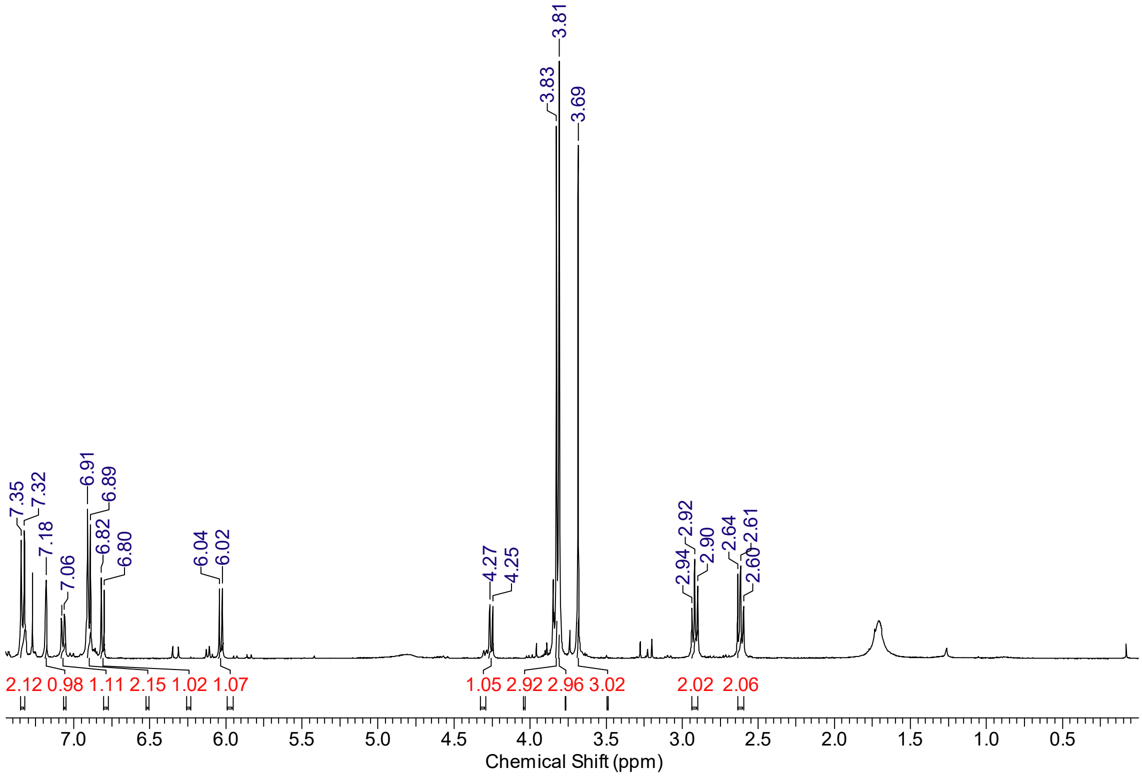

Figure S35. ^{1}H NMR spectra of compound **18** ($CDCl_3$, 400 MHz, TMS).

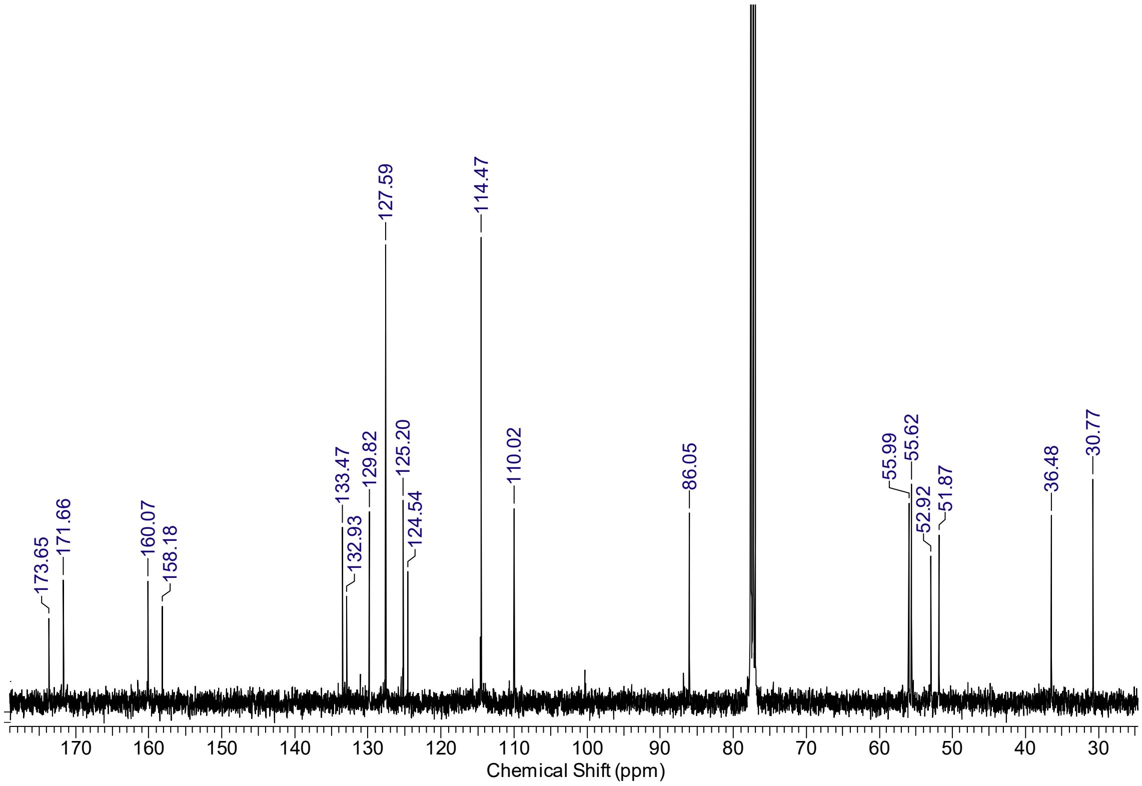

Figure S36. ^{13}C NMR spectra of compound **18** ($CDCl_3$, 400 MHz, TMS).

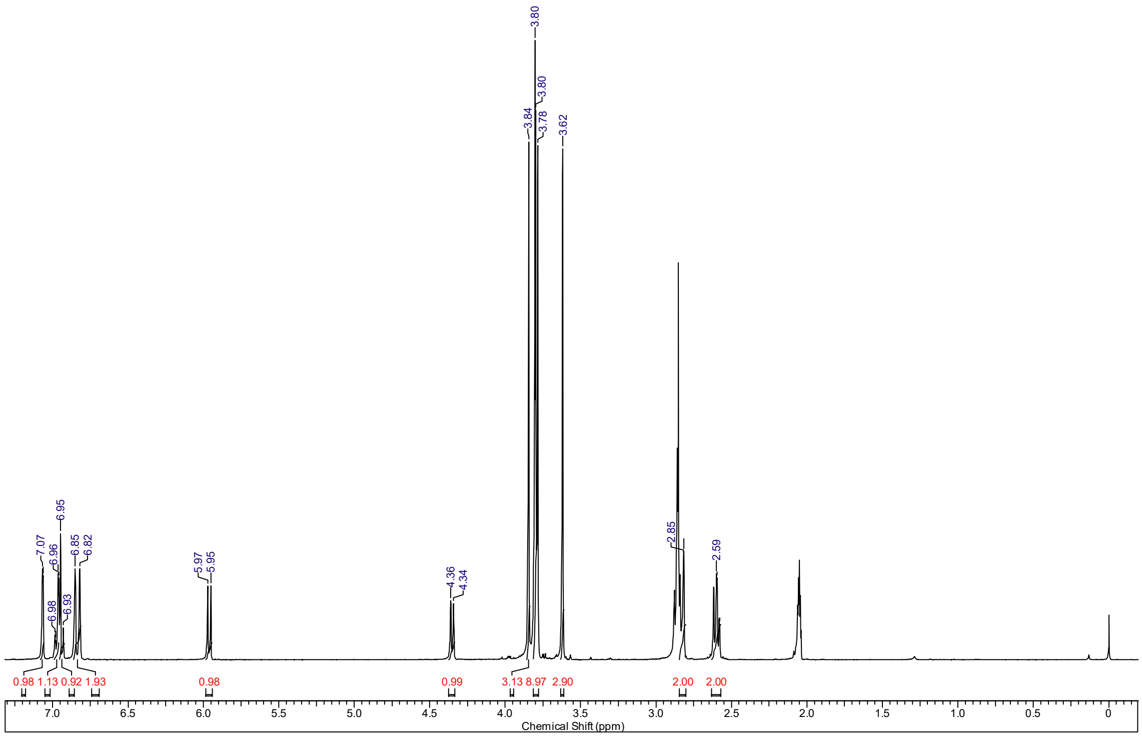

Figure S37. ^{1}H NMR spectra of compound **19** (*Acetone-d$_6$*, 400 MHz, TMS).

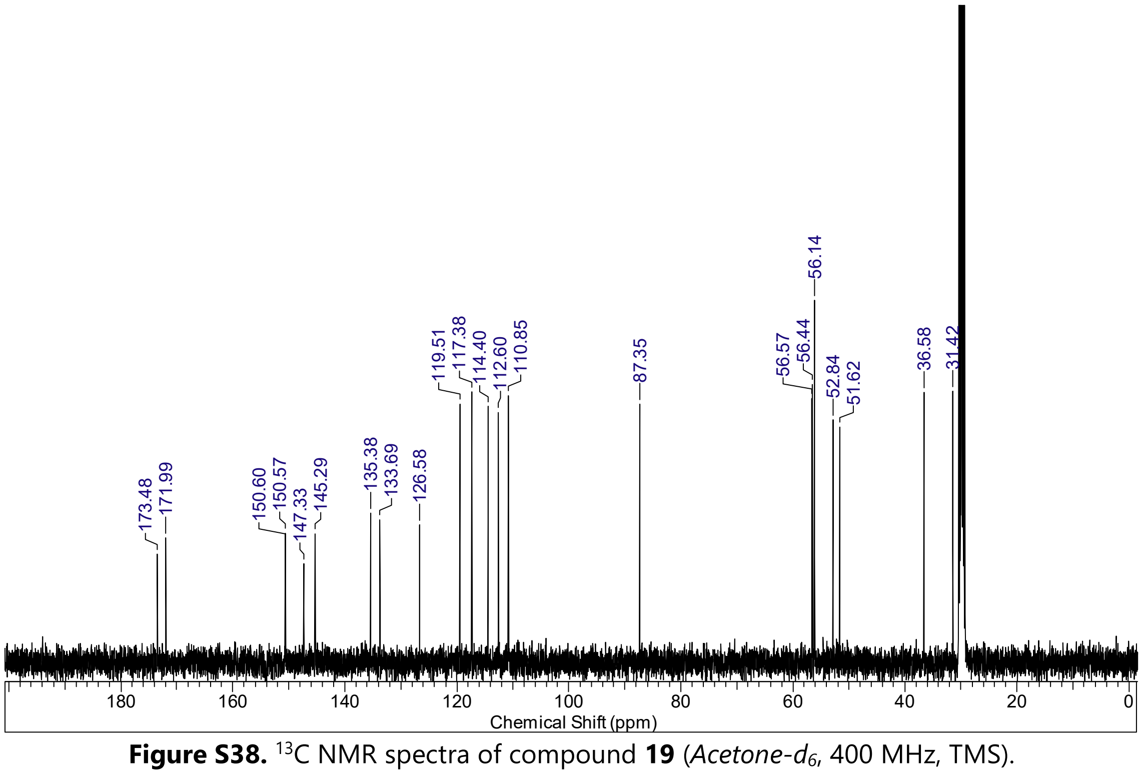

Figure S38. ^{13}C NMR spectra of compound **19** (*Acetone-d$_6$*, 400 MHz, TMS).

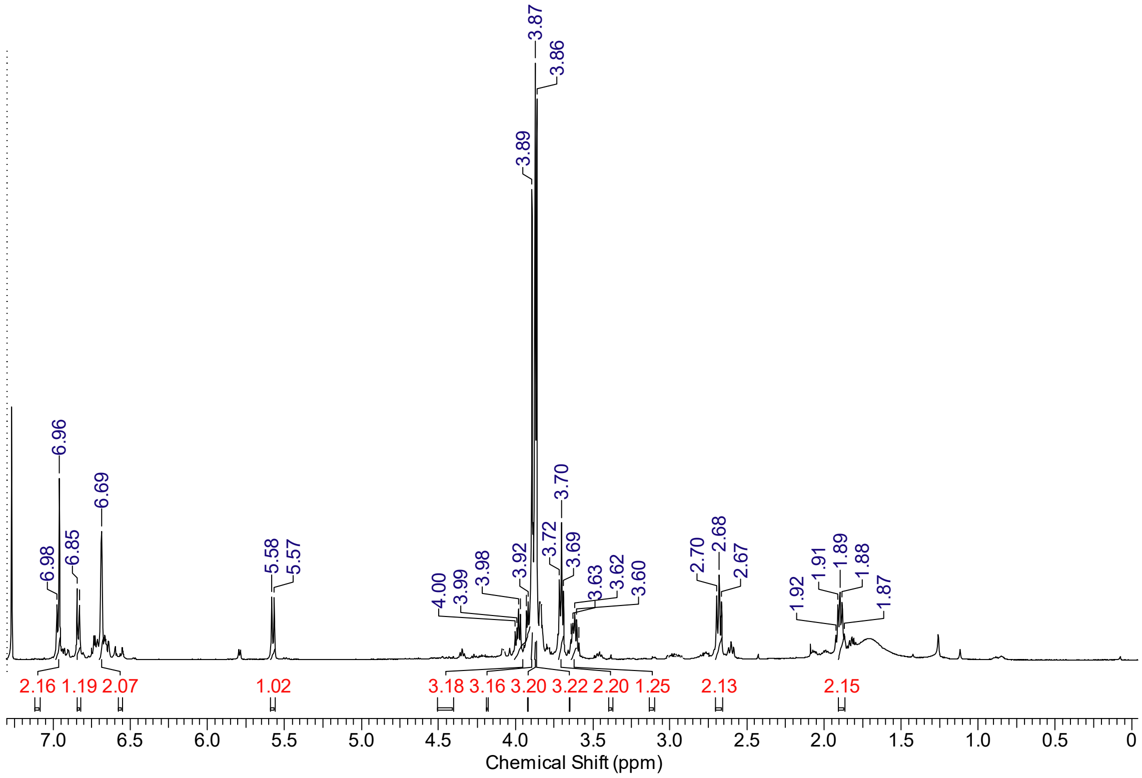

Figure S39. ^{1}H NMR spectra of compound **20** (*Acetone-d_6*, 400 MHz, TMS).

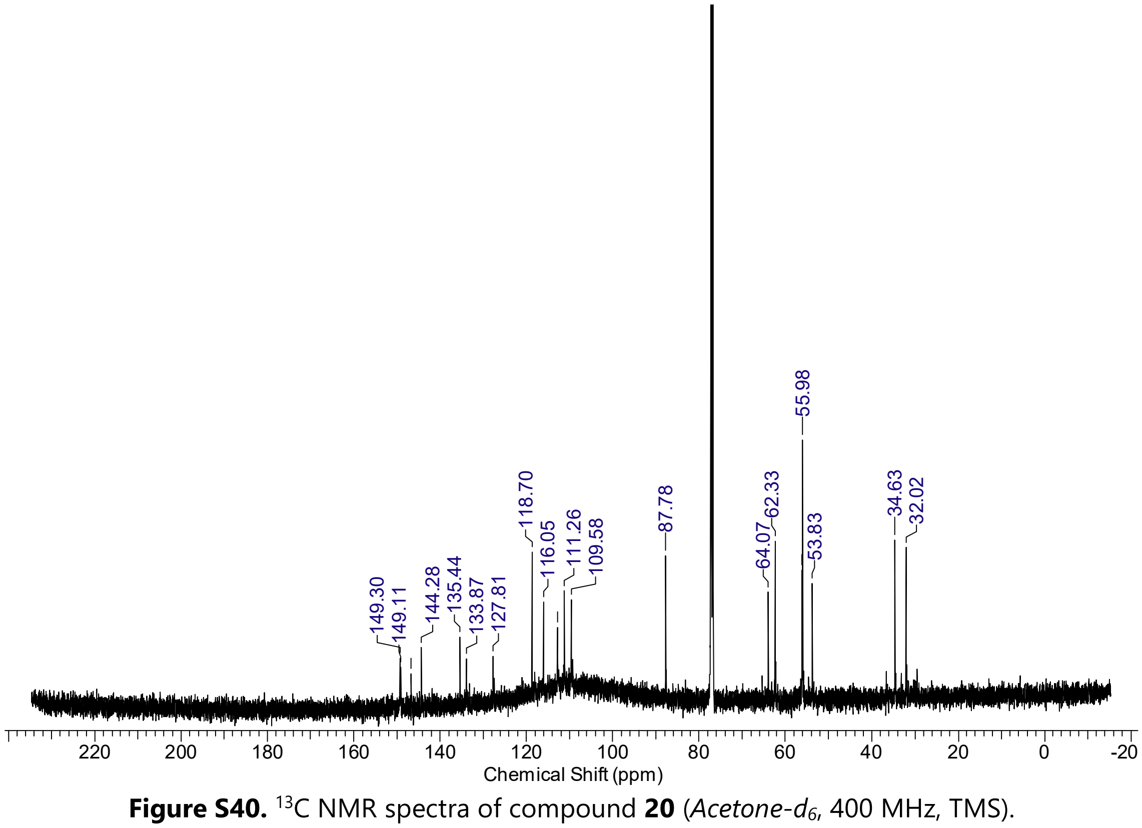

Figure S40. ^{13}C NMR spectra of compound **20** (*Acetone-d_6*, 400 MHz, TMS).

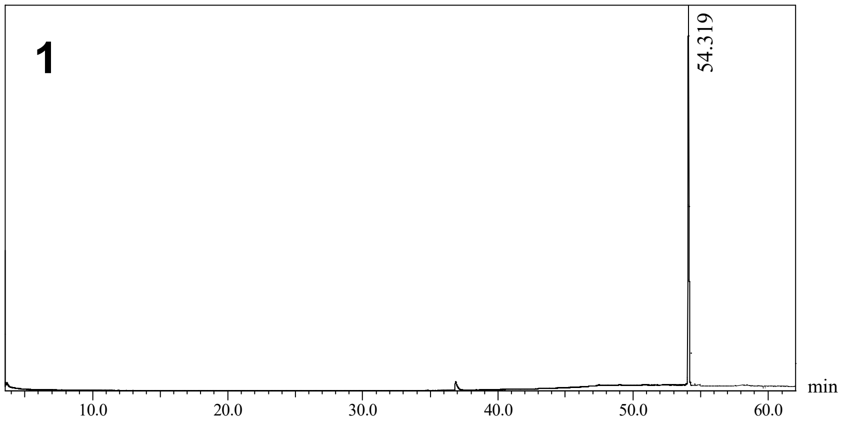

Figure S41. GC-FID chromatogram of compound **1**.

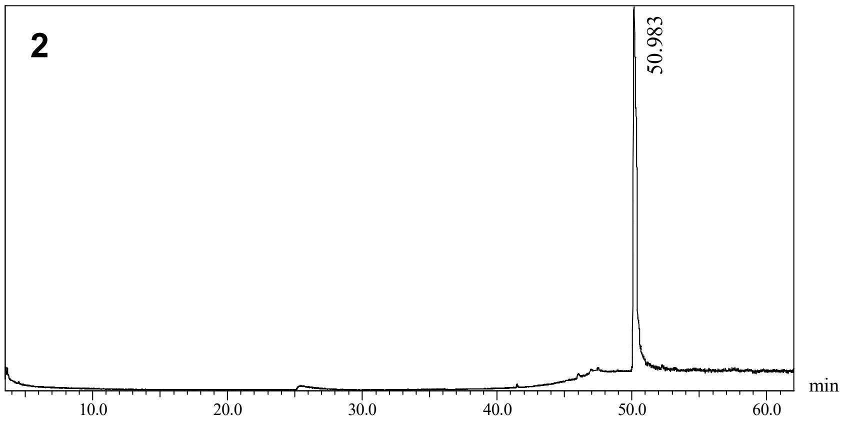

Figure S42. GC-FID chromatogram of compound **2**.

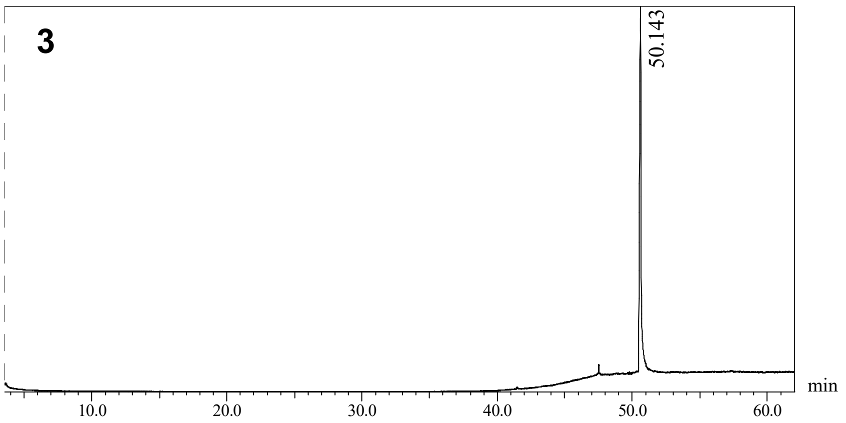

Figure S43. GC-FID chromatogram of compound **3**.

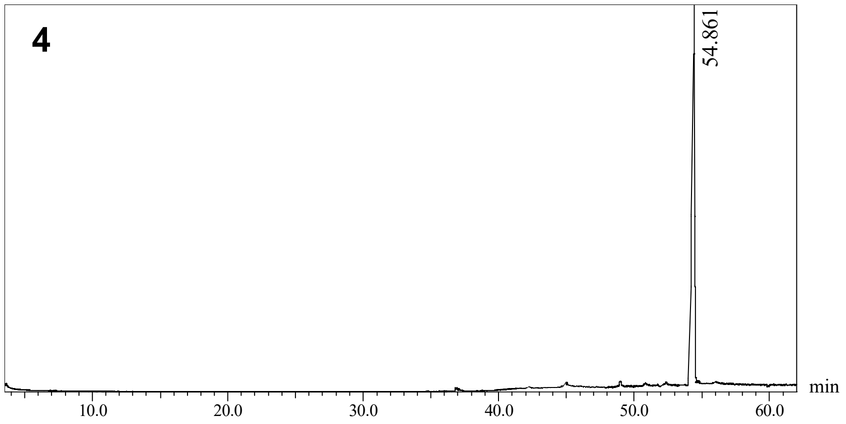

Figure S44. GC-FID chromatogram of compound **4**.

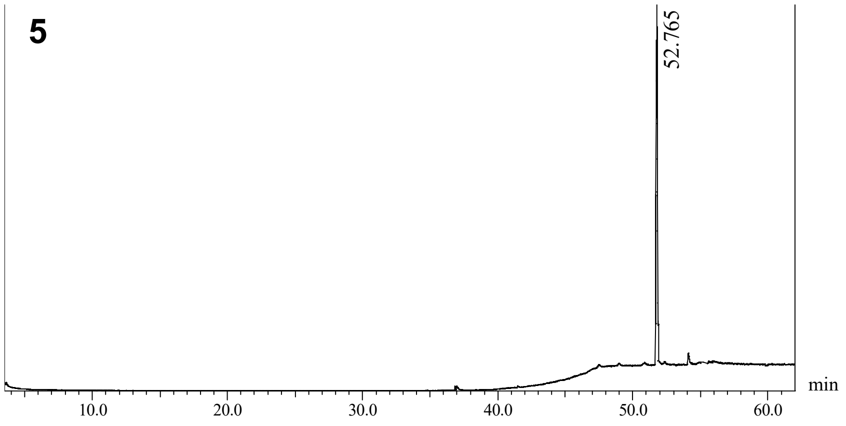

Figure S45. GC-FID chromatogram of compound **5**.

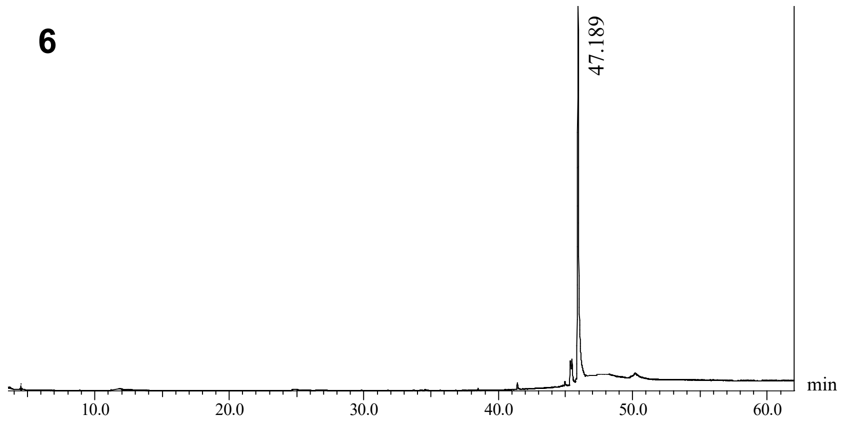

Figure S46. GC-FID chromatogram of compound **6**.

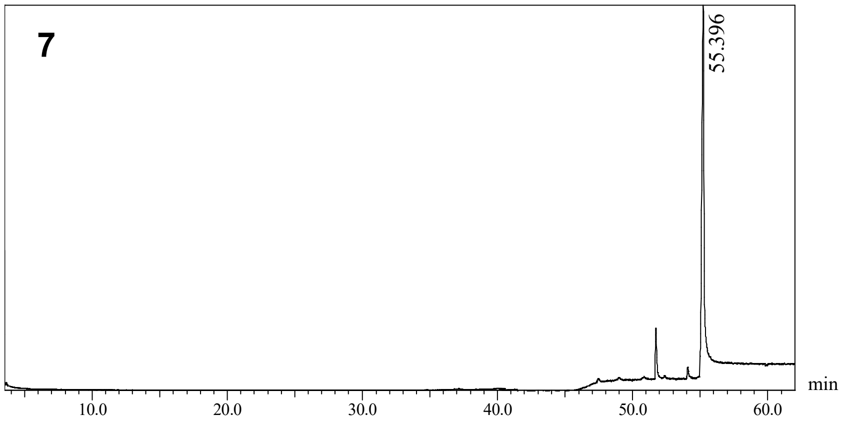

Figure S47. GC-FID chromatogram of compound **7**.

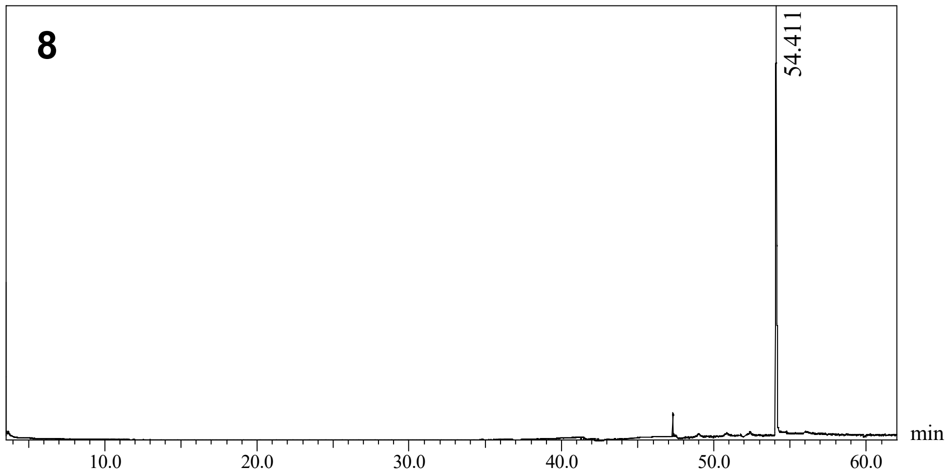

Figure S48. GC-FID chromatogram of compound **8**.

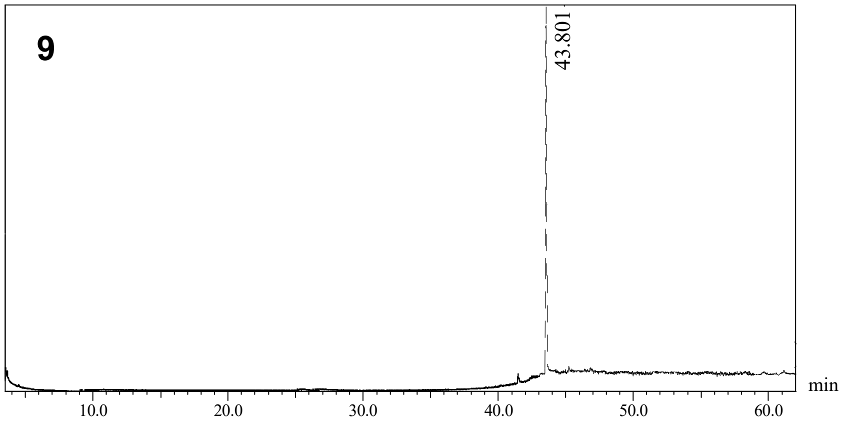

Figure S49. GC-FID chromatogram of compound **9**.

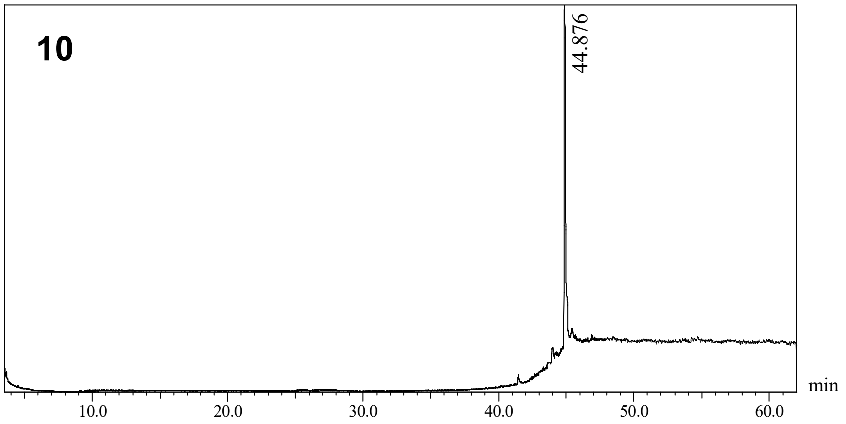

Figure S50. GC-FID chromatogram of compound **10**.

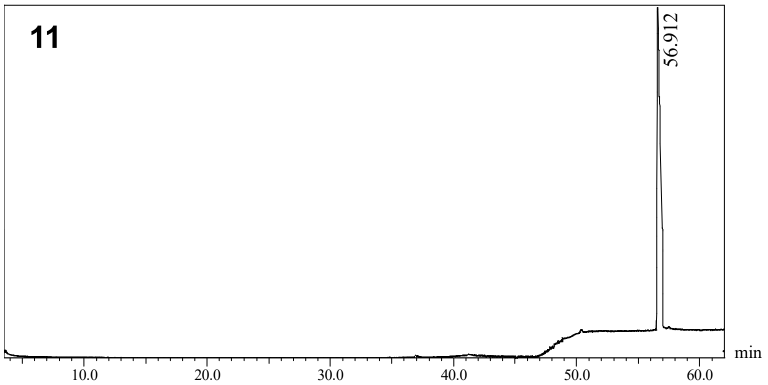

Figure S51. GC-FID chromatogram of compound **11**.

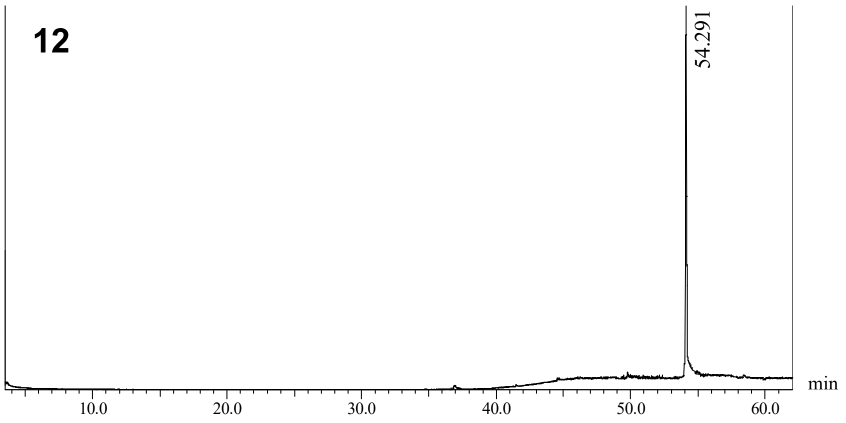

Figure S52. GC-FID chromatogram of compound **12**.

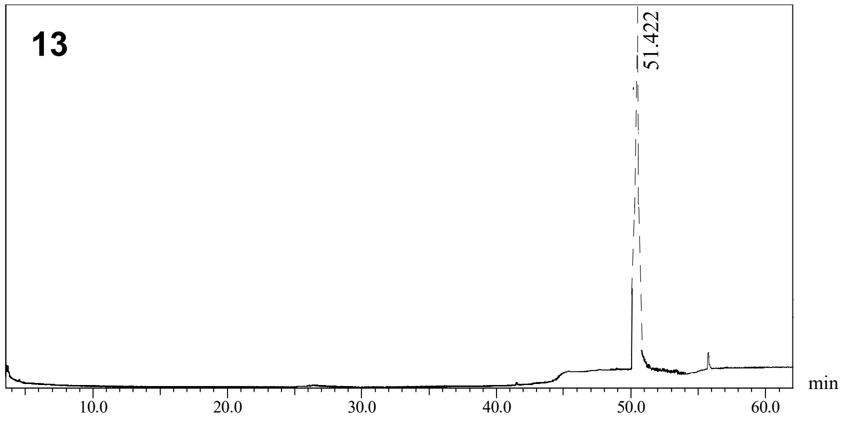

Figure S53. GC-FID chromatogram of compound **13**.

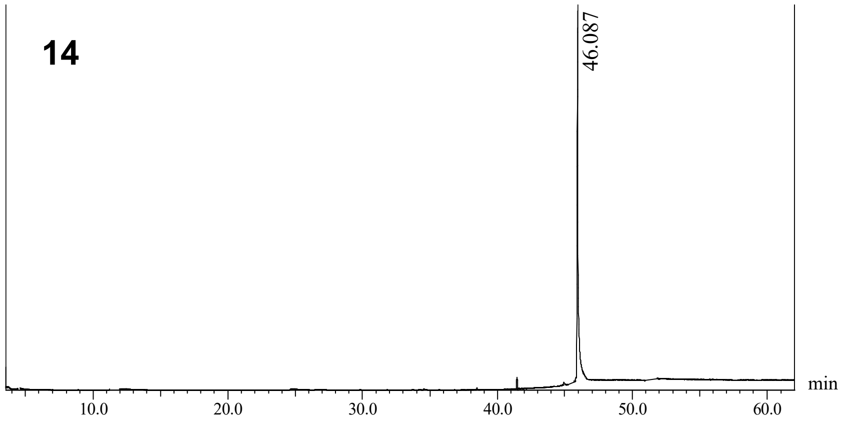

Figure S54. GC-FID chromatogram of compound **14**.

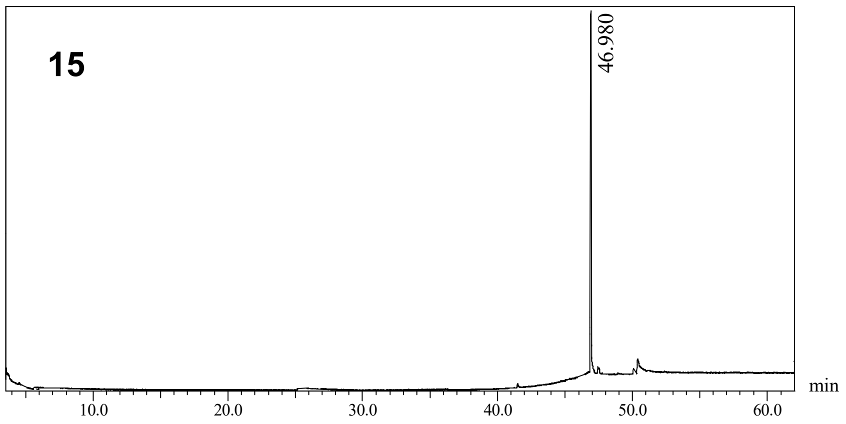

Figure S55. GC-FID chromatogram of compound **15**.

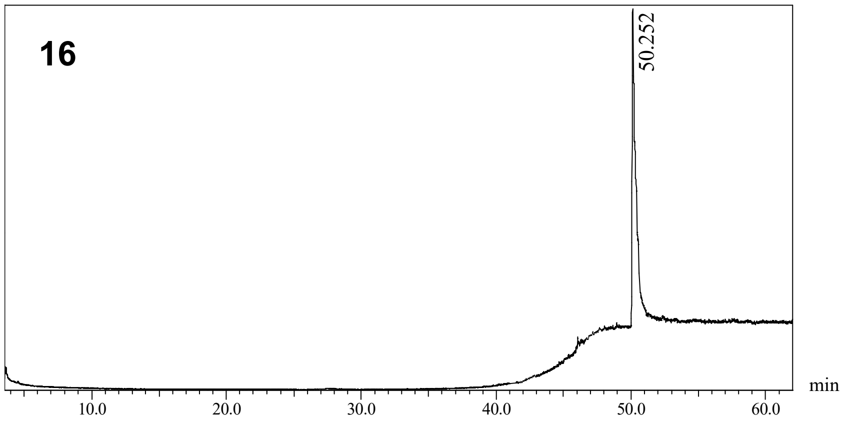

Figure S56. GC-FID chromatogram of compound **16**.

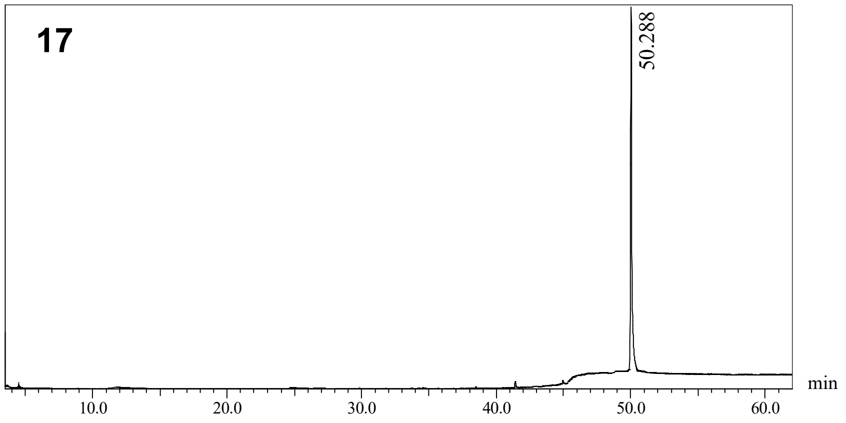

Figure S57. GC-FID chromatogram of compound **17**.

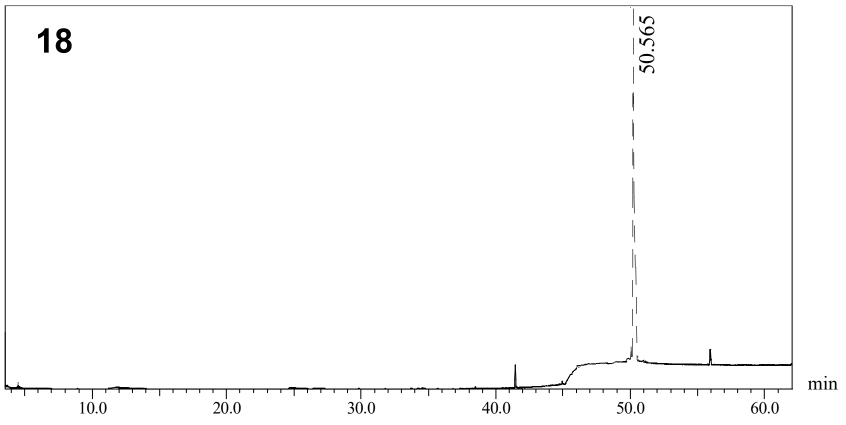

Figure S58. GC-FID chromatogram of compound **18**.

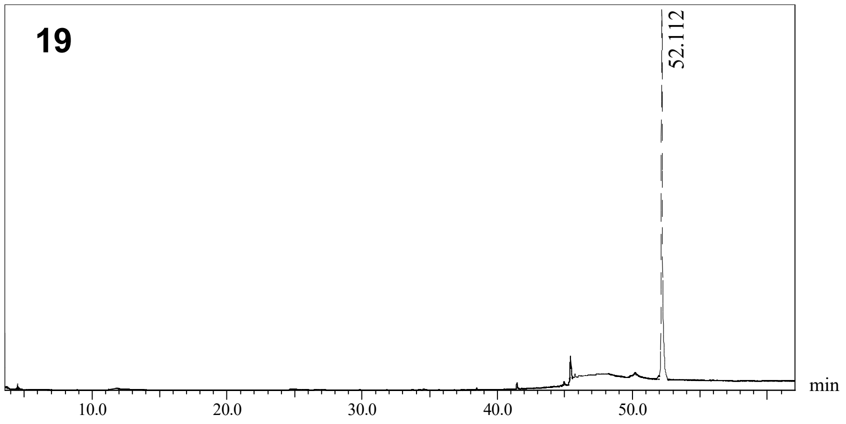

Figure S59. GC-FID chromatogram of compound **19**.

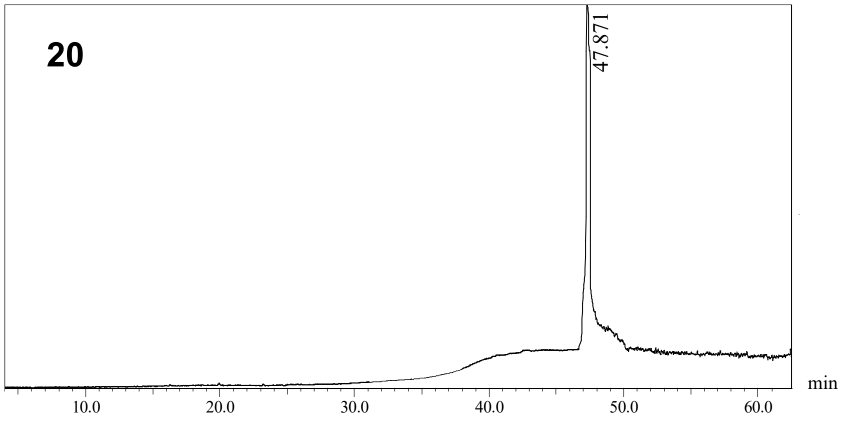

Figure S60. GC-FID chromatogram of compound **20**.

Experimental Information

Compounds **1-20** were identified on the basis of their nuclear magnetic resonance (NMR) data. The ^{1}H and ^{13}C NMR analysis of compounds **1-5** were achieved in a spectrometer Bruker DRX-400, operating in 400 MHz for ^{1}H and 100 MHz for ^{13}C. The samples were dissolved in *Acetone-d_6* (99.8 atom % D, Sigma-Aldrich), or $CDCl_3$ (99.8 atom % D, Sigma-Aldrich) with TMS (0.01%) as internal standard. The chemical shifts (δ) were expressed in parts per million (ppm) relative the residual solvent peak, and the multiplicity of signals was deduced according to the signals obtained in the spectrum. The coupling constants (*J*, Hz) were calculated in comparison to the same signal peaks, as well as the relative integral, from which was deduced the number of hydrogens.

Gas chromatography with flame ionization detection (GC-FID) analyses was performed on a GC-2010 Plus (Shimadzu Corp, Kyoto, Japan) system. Compounds **1-20** were dissolved in ethyl acetate and injected with the ad of an OAC-20s autosampler. The injector temperature was set at 250 ºC. Nitrogen (N_2, 99.999 %) was used as carrier gas. An Rtx-5 (30 m x 0.25 mm x 0.25 μm) capillary column (Restek Co., Bellefonte, PA, USA) was used. The column temperature was programmed to increase from 70 ºC to 290 ºC at 3 ºC/min, then kept at 290 ºC for 15 min. The purity of compounds **1-20** was estimated based on the relative peak area in the GC-FID chromatogram.
